# Supplementary material for: Serratus Anterior Plane Block Remote Learning Curriculum
Source: MedEdPORTAL. 2024 Oct 25;20:11454. doi: 10.15766/mep_2374-8265.11454 (PMC11502517; doi:10.15766/mep_2374-8265.11454)
Supplement: Supplementary file 1 — Serratus Anterior Block Presentation.pptxSAPB Kahoot Quiz.pptxSAPB Proctor Instructions.docxQualtrics Presession Survey.docxQualtrics Postsession Survey.docx [file mep_2374-8265.11454-s001.zip › C. SAPB Proctor Instructions.docx]

**Instructions for small group proctors for SAPB teach back session (15 minutes):**

Proctors, please assume the role of a knowledgeable sub-intern who has just completed their US elective. We will do PRECISELY what we are told, no more and no less. If there is insufficient detail, prompt the team for precision/detail.

You will share your screen on the device with the ultrasound image and make sure the computer camera of another device is pointed at your hands performing the procedure.

Leave procedural materials out of view until they are called for by participating learners. Time the group so they can compete.

Each member of the small group must contribute instructions for at least one discrete step in the procedure.

Checklist:

- Obtain patient consent
- Set-up location (If learners do not verbalize specifics, ask prompting questions such as “patient is in a hallway bed, is that ok?” or “Do we need telemetry?”)
- Materials: monitor, consent forms, 18G blunt tipped needle > 9cm, needle to draw up anesthetic, tubing to connect anesthetic to needle, anesthetic (we will use a flush to represent), sterile probe covers, sterile gel, sterile gloves, sterilizing solution, ultrasound machine, linear/curvilinear transducer, gauze, table. Start with these items under the table and place them in the field of view learners call for them.
- Anatomy and ultrasound identification (must ID the pleural line, the rib, the serratus, NOT the latissimus dorsi given animal rib variation. Must ID site to administer anesthetic. Proctor should recommend deep plane adjacent to rib given animal rib anatomic variation)
- Sterilize
- Subcutaneous anesthesia
- Must orient probe and needle tip in plane (for best visualization, recommend probe perpendicular not parallel to rib given constraints of animal ribs)
- Eyes on needle-tip!
- Trace needle tip to fascial layer
- Hydrodissect SLOWLY
- Administer anesthesia
- RECORD TIME! Rejoin the large group.
