# Supplementary material for: Serratus Anterior Plane Block Remote Learning Curriculum
Source: MedEdPORTAL. 2024 Oct 25;20:11454. doi: 10.15766/mep_2374-8265.11454 (PMC11502517; doi:10.15766/mep_2374-8265.11454)
Supplement: Supplementary file 1 — Serratus Anterior Block Presentation.pptxSAPB Kahoot Quiz.pptxSAPB Proctor Instructions.docxQualtrics Presession Survey.docxQualtrics Postsession Survey.docx [file mep_2374-8265.11454-s001.zip › D. Qualtrics Presession Survey.docx]

Serratus Anterior Block Pre-Session Survey

Start of Block: Please answer each questions with the scales below

Pre-survey Q1 What is your current level of medical training?

- Medical Student (1)
- EM PGY1 (2)
- EM PGY2 (3)
- EM PGY3 (4)
- EM PGY4 (5)
- Non-EM resident (6)
- Attending (7)
- PA/NP (8)
- PA fellow/resident (9)
- Other (10) __________________________________________________

Pre-survey Q2 How confident do you feel in performing a serratus anterior block on a patient?

- Not at all confident (1)
- Slightly confident (2)
- Moderately confident (3)
- Very confident (4)
- Extremely confident (5)

Pre-Survey Q3 How often do you perform a serratus anterior block on a patient?

- Less than once a year (1)
- 1-10 (2)
- Once a month (3)
- 1-3 times a month (4)
- More than once a week (5)

Pre-survey Q4 Which of the following are barriers to you including ultrasound-guided regional anesthesia (nerve and plane block) procedures in your clinical practice? You may select any number.

- Time (1)
- Space (2)
- Staffing (3)
- Materials not stocked/accessible (4)
- Lack of expertise with anatomy (5)
- Lack of expertise with ultrasound (6)
- Lack of experience with procedure (7)
- Lack of supervision (8)
- Lack of attending comfort with procedure (9)
- Other (10) __________________________________________________

End of Block: Please answer each questions with the scales below
