# Supplementary material for: Serratus Anterior Plane Block Remote Learning Curriculum
Source: MedEdPORTAL. 2024 Oct 25;20:11454. doi: 10.15766/mep_2374-8265.11454 (PMC11502517; doi:10.15766/mep_2374-8265.11454)
Supplement: Supplementary file 1 — Serratus Anterior Block Presentation.pptxSAPB Kahoot Quiz.pptxSAPB Proctor Instructions.docxQualtrics Presession Survey.docxQualtrics Postsession Survey.docx [file mep_2374-8265.11454-s001.zip › E. Qualtrics Postsession Survey.docx]

Serratus Anterior Block Post-Session Evaluation

Start of Block: Please answer each questions with the scales below

Post-survey Q1 What is your current level of medical training?

- Medical Student (1)
- EM PGY1 (2)
- EM PGY2 (3)
- EM PGY3 (4)
- EM PGY4 (5)
- Non-EM resident (6)
- Attending (7)
- PA/NP (8)
- PA fellow/resident (9)
- Other (10) __________________________________________________

Post-survey Q2 How satisfied were you with the session?

- Not at all satisfied (1)
- Slightly dissatisfied (2)
- Moderately satisfied (3)
- Very satisfied (4)
- Extremely satisfied (5)

Post-survey Q3 How confident do you feel in performing a serratus anterior block on a patient?

- Not at all confident (1)
- Slightly confident (2)
- Moderately confident (3)
- Very confident (4)
- Extremely confident (5)

Post-survey Q4 How relevant was the session to your clinical practice? 

- Not relevant (1)
- Slightly relevant (2)
- Moderately relevant (3)
- Very relevant (4)
- Extremely relevant (5)

Post-survey Q5 How much will you incorporate the content of this session into your own work?

- None at all (1)
- A little (2)
- A moderate amount (3)
- A lot (4)
- A great deal (5)

Post-survey Q6 Would you be interested in future sessions similar to this one during future conferences?

- Yes! (1)
- No! (2)
- Maybe (3)

Post-survey Q7 Please provide any feedback you may have to help us improve our session:

________________________________________________________________

________________________________________________________________

________________________________________________________________

________________________________________________________________

________________________________________________________________

End of Block: Please answer each questions with the scales below
