# Supplementary material for: Serratus Anterior Plane Block Remote Learning Curriculum
Source: MedEdPORTAL. 2024 Oct 25;20:11454. doi: 10.15766/mep_2374-8265.11454 (PMC11502517; doi:10.15766/mep_2374-8265.11454)
Supplement: Supplementary file 1 — Serratus Anterior Block Presentation.pptxSAPB Kahoot Quiz.pptxSAPB Proctor Instructions.docxQualtrics Presession Survey.docxQualtrics Postsession Survey.docx [file mep_2374-8265.11454-s001.zip › A. Serratus Anterior Block Presentation.pptx]

## Slide 1
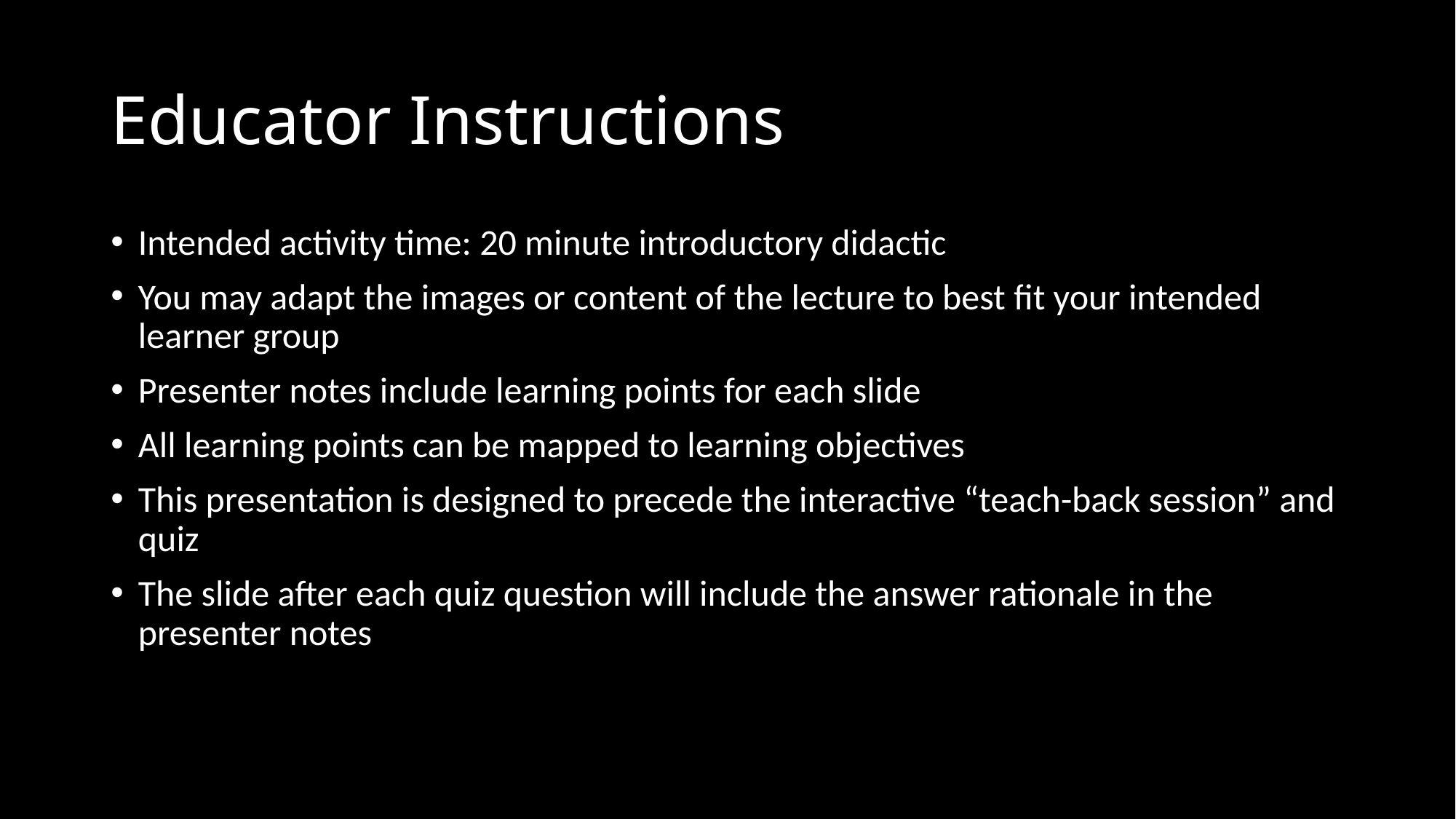

# Educator Instructions
Intended activity time: 20 minute introductory didactic
You may adapt the images or content of the lecture to best fit your intended learner group
Presenter notes include learning points for each slide
All learning points can be mapped to learning objectives
This presentation is designed to precede the interactive “teach-back session” and quiz
The slide after each quiz question will include the answer rationale in the presenter notes

## Slide 2
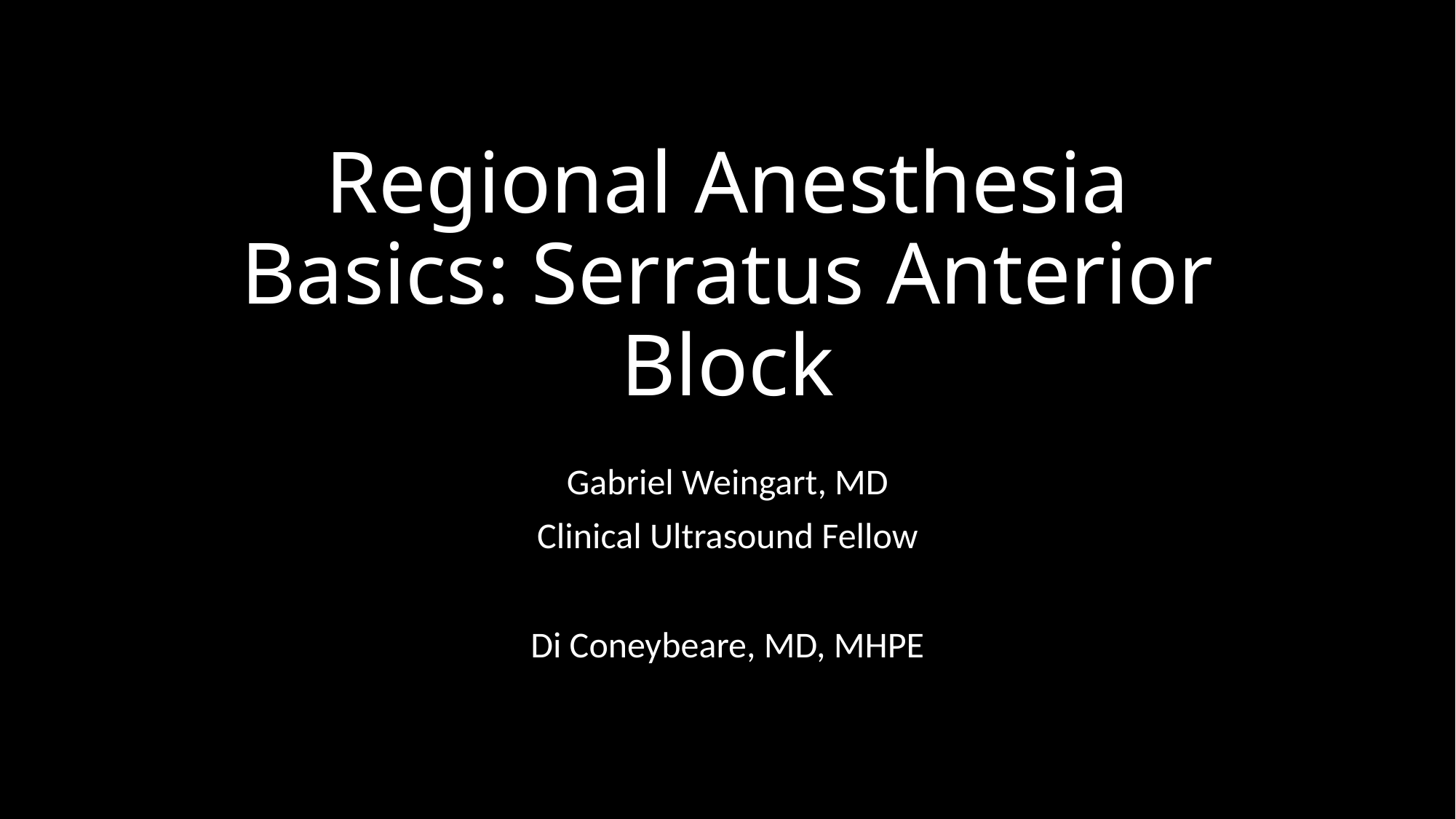

# Regional Anesthesia Basics: Serratus Anterior Block
Gabriel Weingart, MD
Clinical Ultrasound Fellow
Di Coneybeare, MD, MHPE

## Slide 3
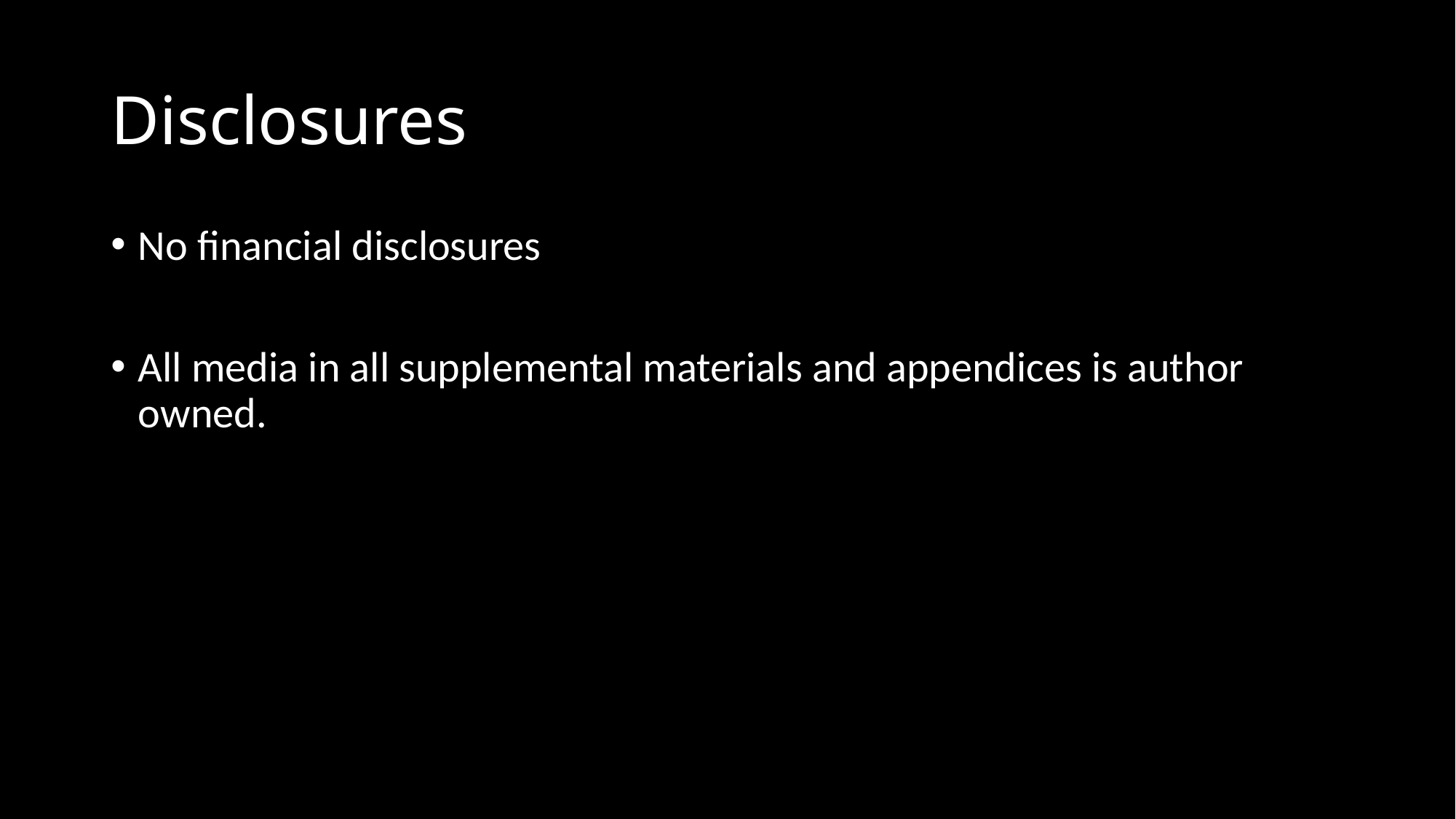

# Disclosures
No financial disclosures
All media in all supplemental materials and appendices is author owned.

## Slide 4
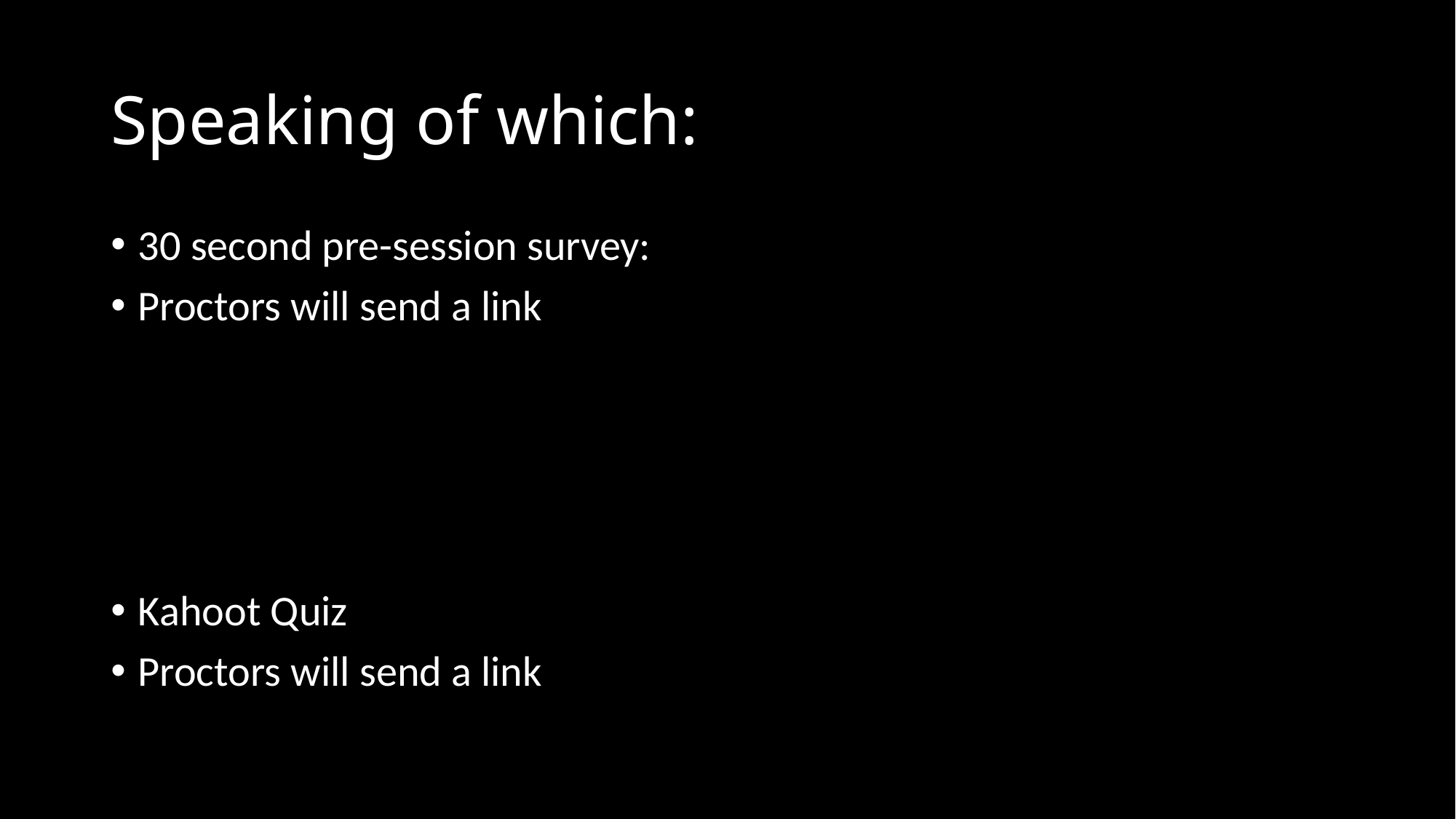

# Speaking of which:
30 second pre-session survey:
Proctors will send a link
Kahoot Quiz
Proctors will send a link

## Slide 5
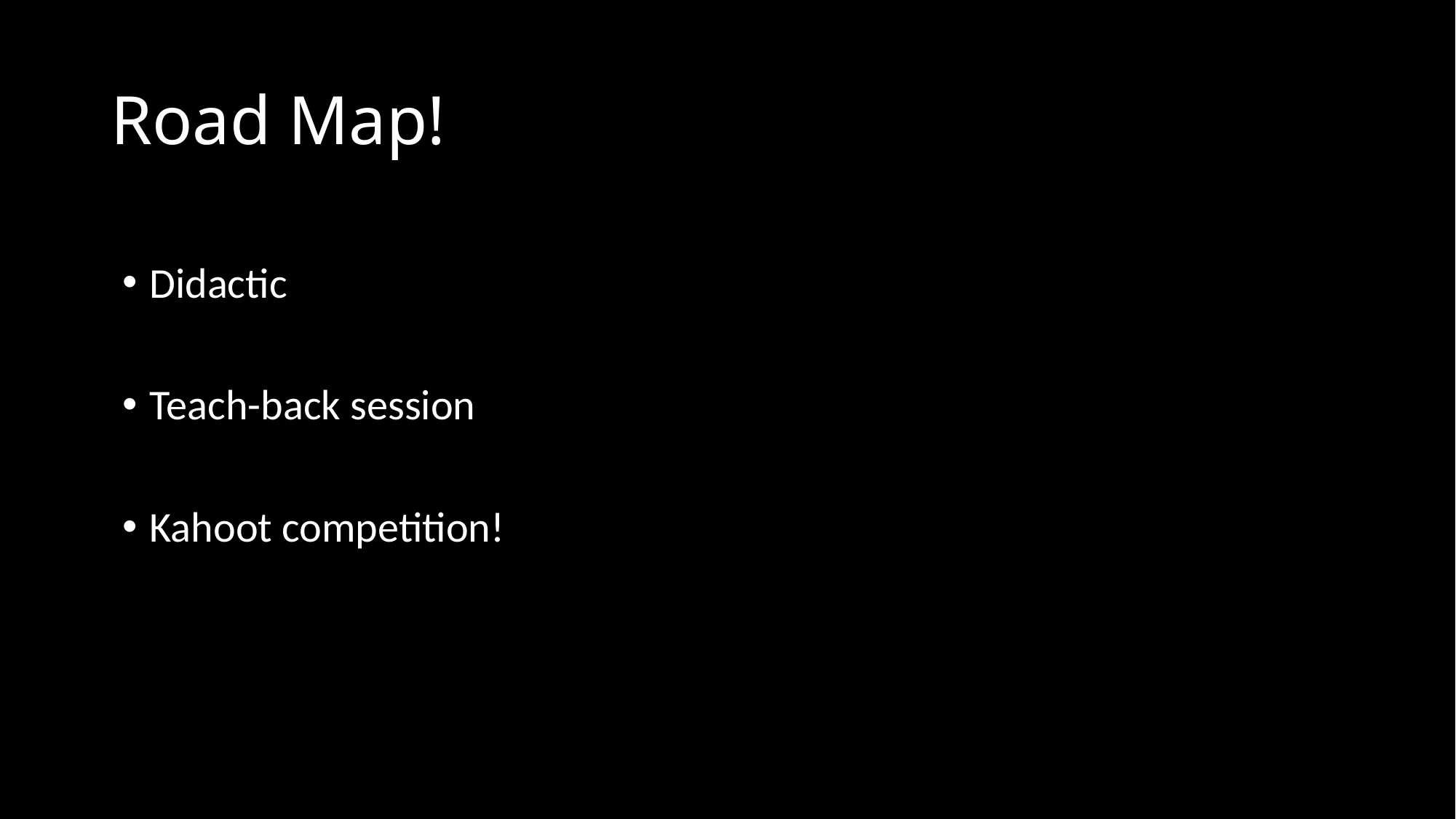

# Road Map!
Didactic
Teach-back session
Kahoot competition!

## Slide 6
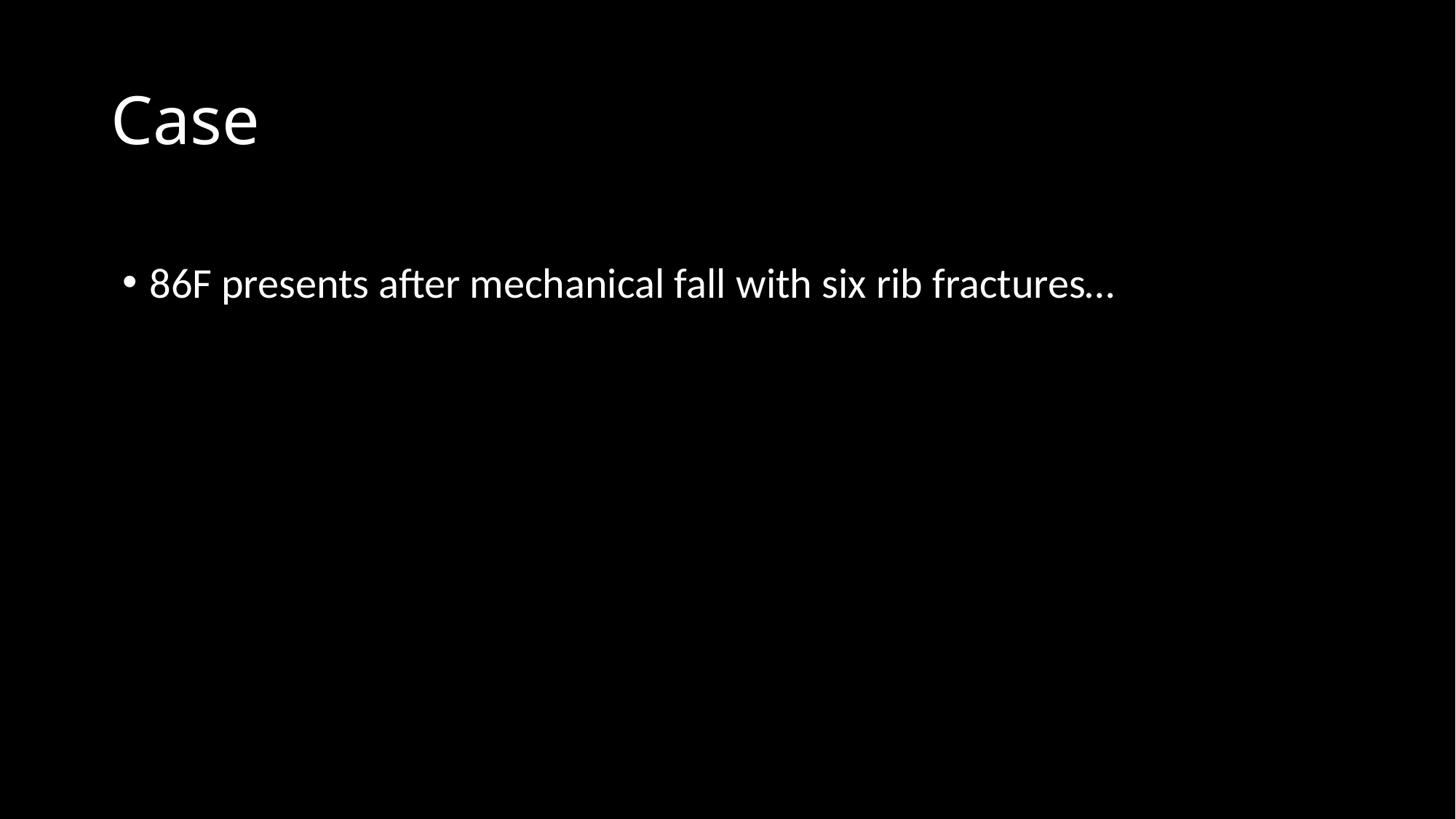

# Case
86F presents after mechanical fall with six rib fractures…

## Slide 7
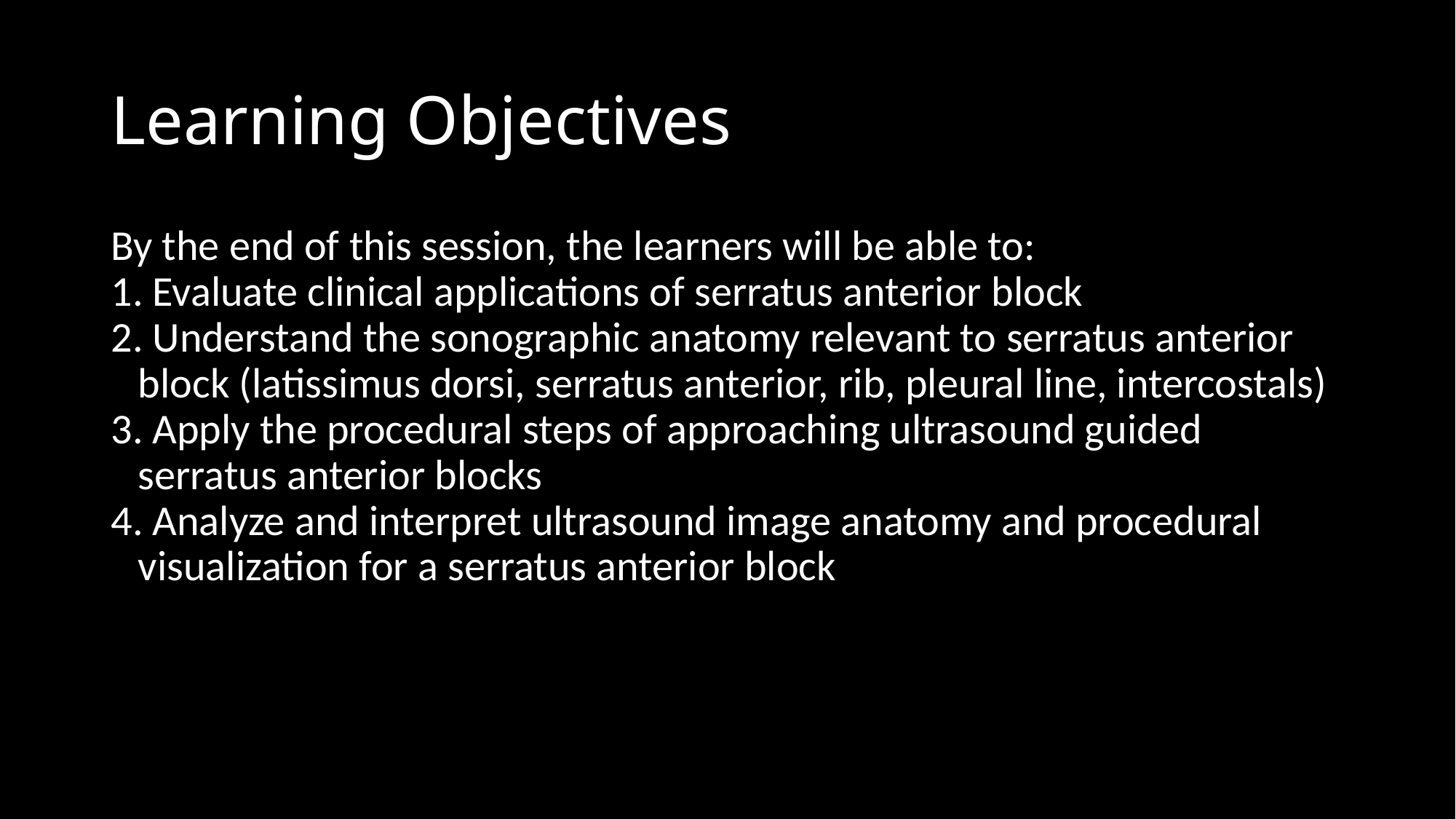

# Learning Objectives
By the end of this session, the learners will be able to:
 Evaluate clinical applications of serratus anterior block
 Understand the sonographic anatomy relevant to serratus anterior block (latissimus dorsi, serratus anterior, rib, pleural line, intercostals)
 Apply the procedural steps of approaching ultrasound guided serratus anterior blocks
 Analyze and interpret ultrasound image anatomy and procedural visualization for a serratus anterior block

## Slide 8
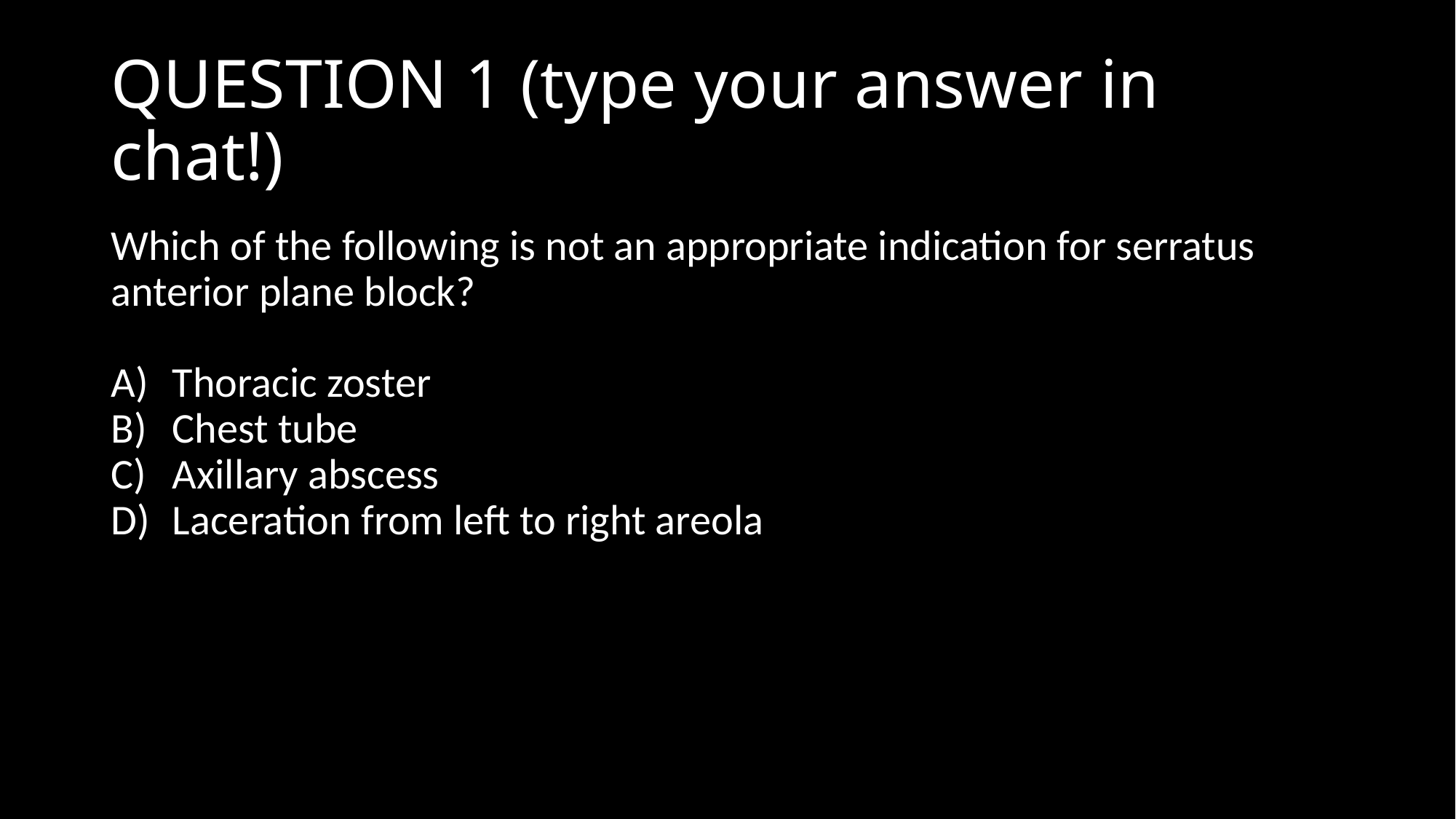

# QUESTION 1 (type your answer in chat!)
Which of the following is not an appropriate indication for serratus anterior plane block?
Thoracic zoster
Chest tube
Axillary abscess
Laceration from left to right areola

## Slide 9
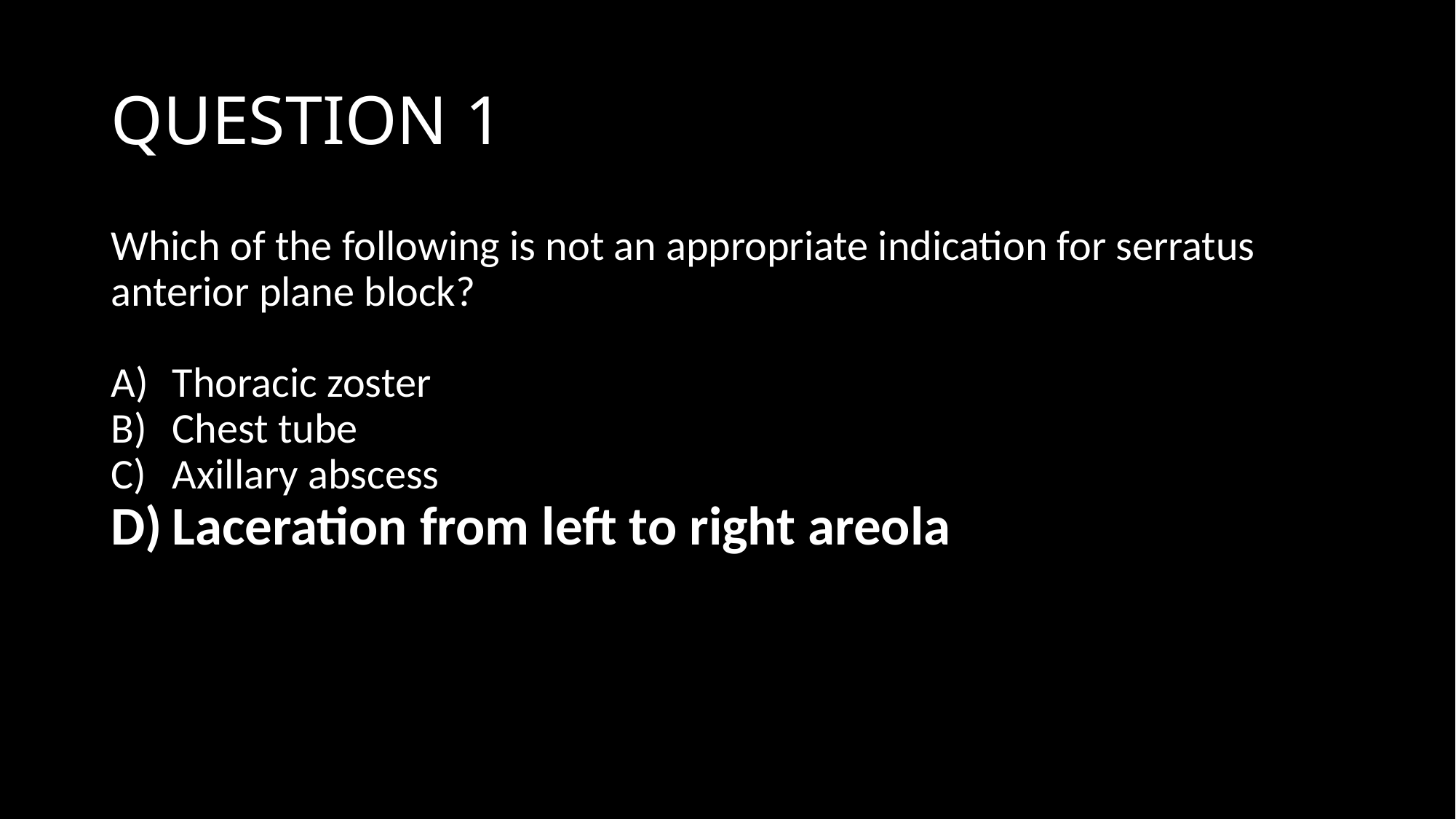

# QUESTION 1
Which of the following is not an appropriate indication for serratus anterior plane block?
Thoracic zoster
Chest tube
Axillary abscess
Laceration from left to right areola

## Slide 10
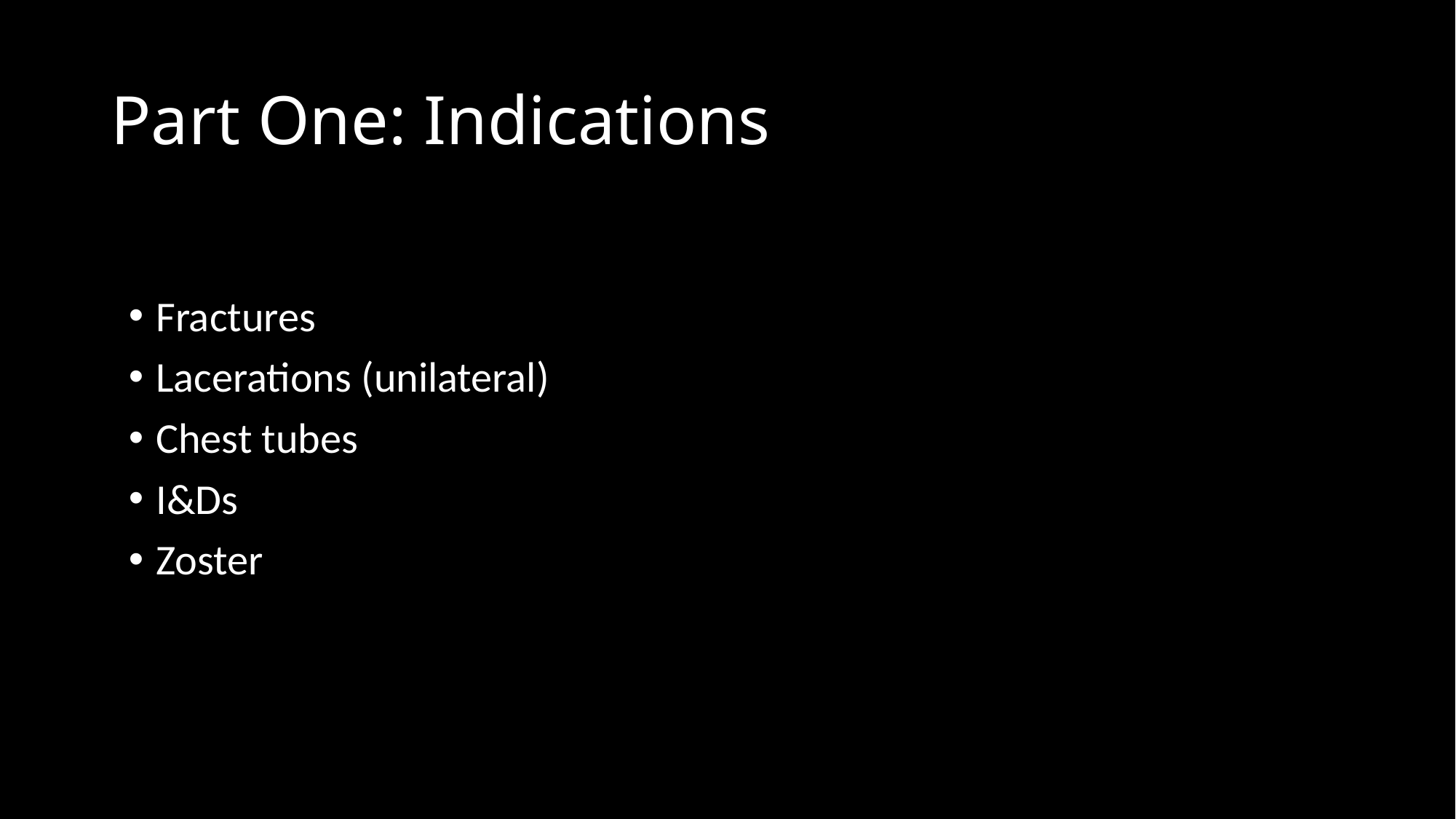

# Part One: Indications
Fractures
Lacerations (unilateral)
Chest tubes
I&Ds
Zoster

## Slide 11
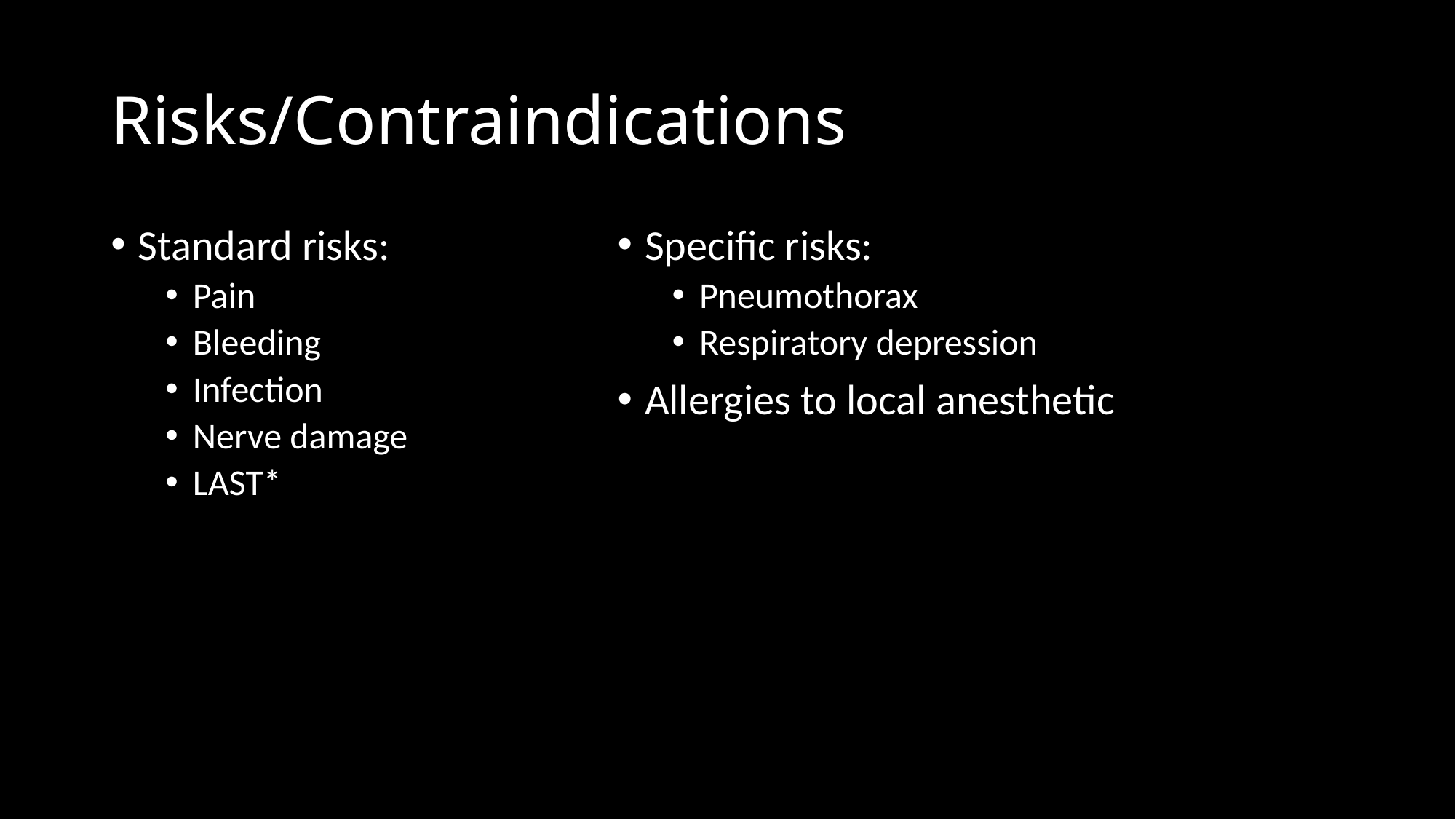

# Risks/Contraindications
Standard risks:
Pain
Bleeding
Infection
Nerve damage
LAST*
Specific risks:
Pneumothorax
Respiratory depression
Allergies to local anesthetic

## Slide 12
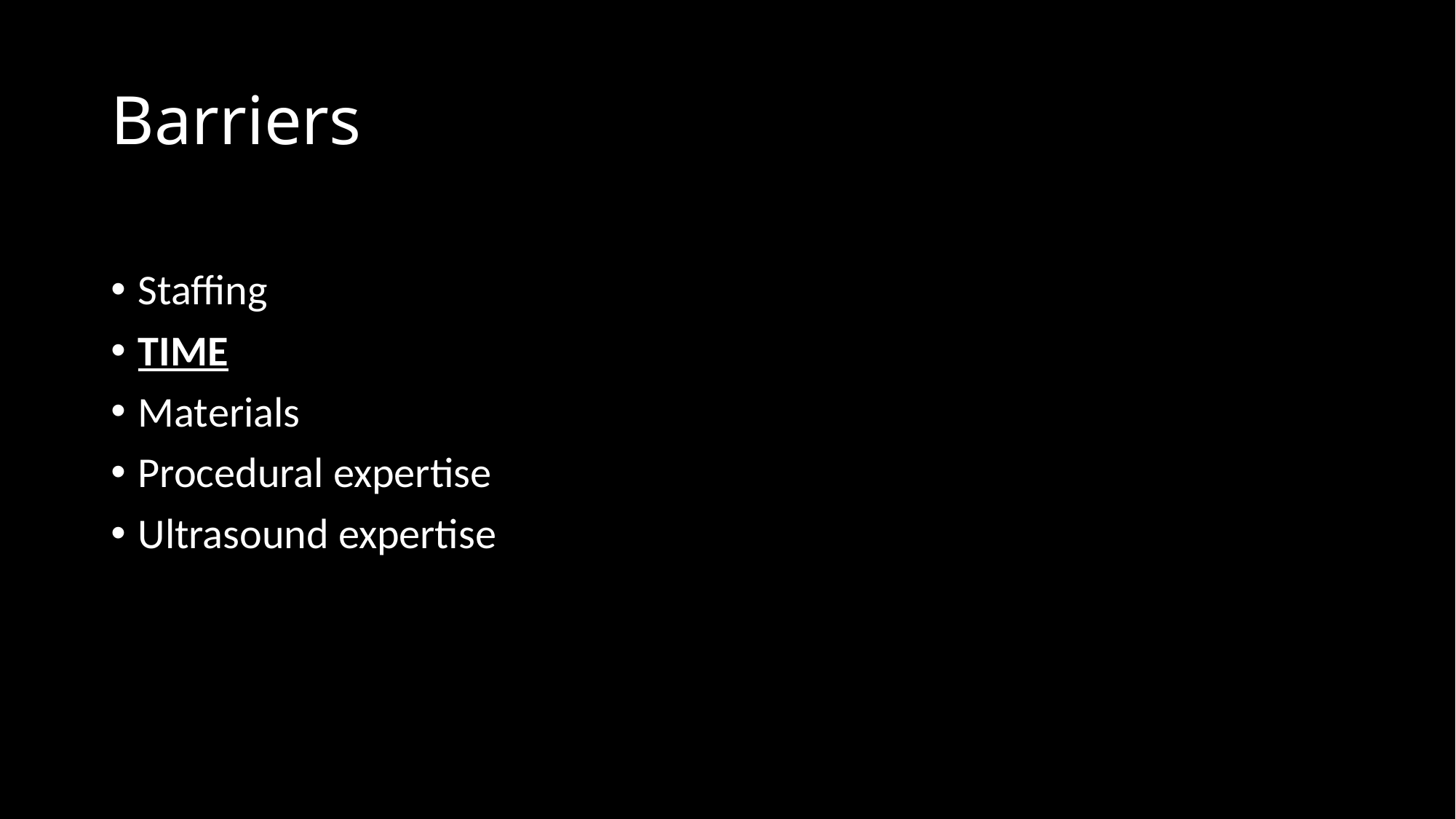

# Barriers
Staffing
TIME
Materials
Procedural expertise
Ultrasound expertise

## Slide 13
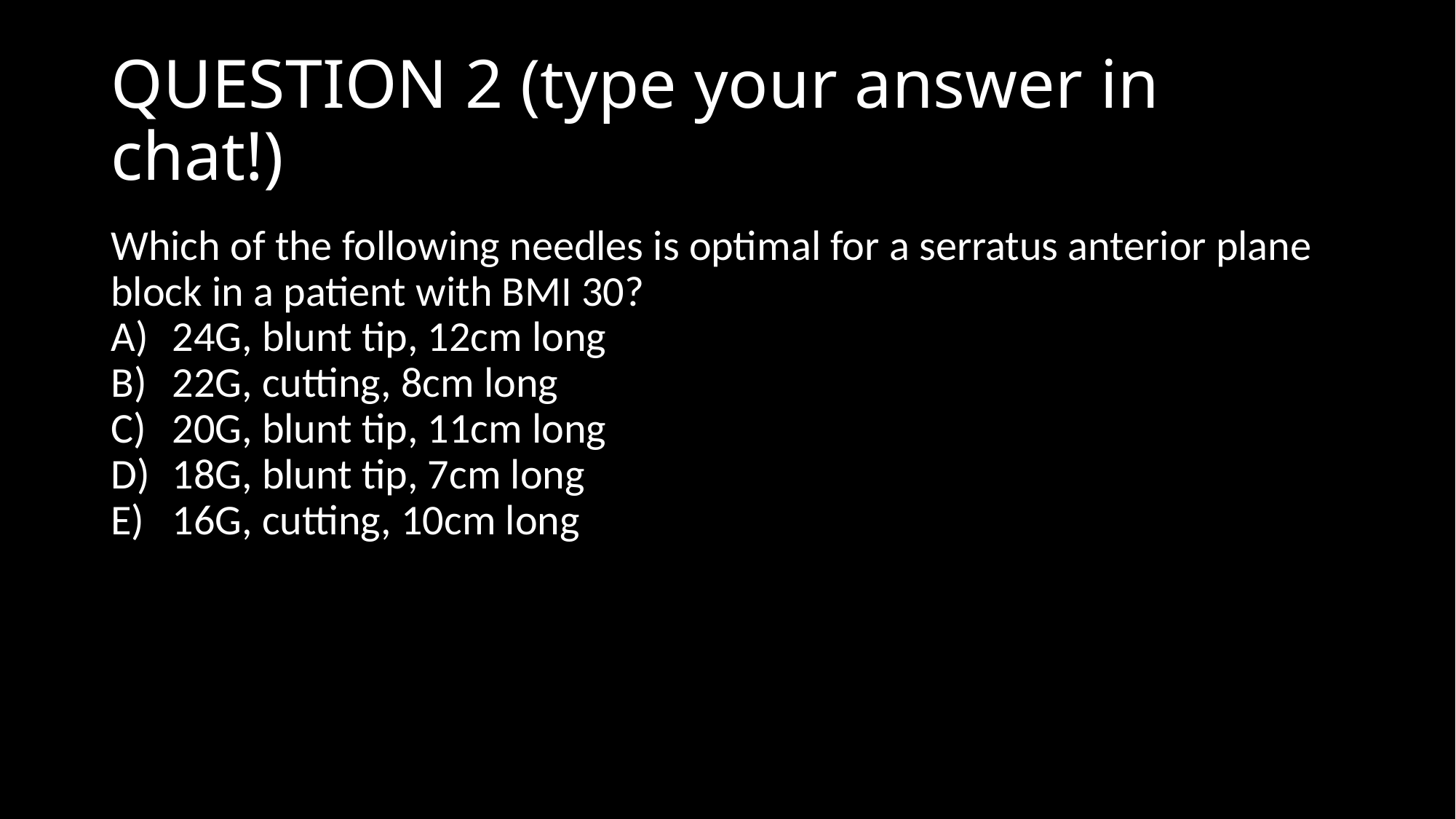

# QUESTION 2 (type your answer in chat!)
Which of the following needles is optimal for a serratus anterior plane block in a patient with BMI 30?
24G, blunt tip, 12cm long
22G, cutting, 8cm long
20G, blunt tip, 11cm long
18G, blunt tip, 7cm long
16G, cutting, 10cm long

## Slide 14
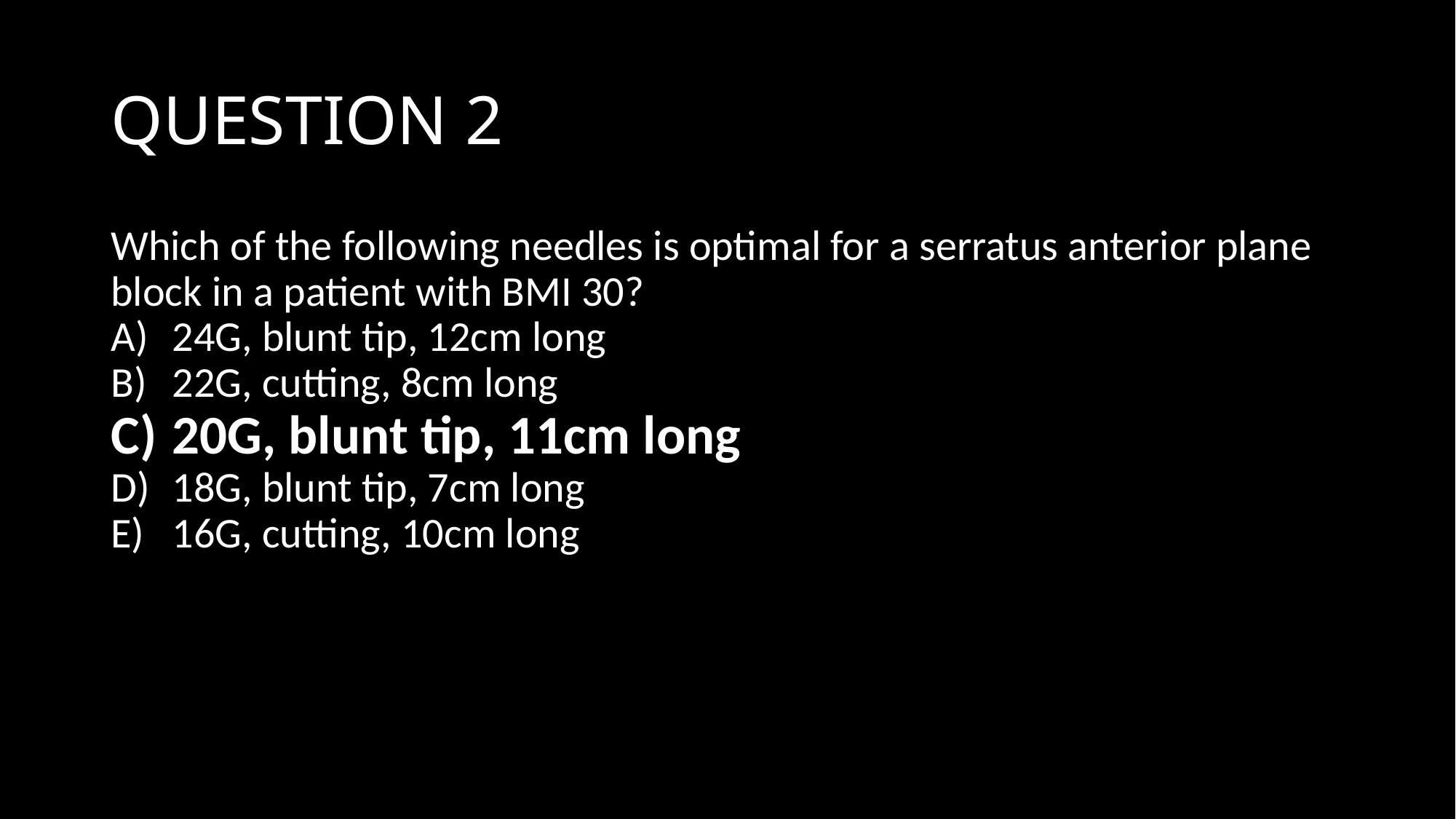

# QUESTION 2
Which of the following needles is optimal for a serratus anterior plane block in a patient with BMI 30?
24G, blunt tip, 12cm long
22G, cutting, 8cm long
20G, blunt tip, 11cm long
18G, blunt tip, 7cm long
16G, cutting, 10cm long

## Slide 15
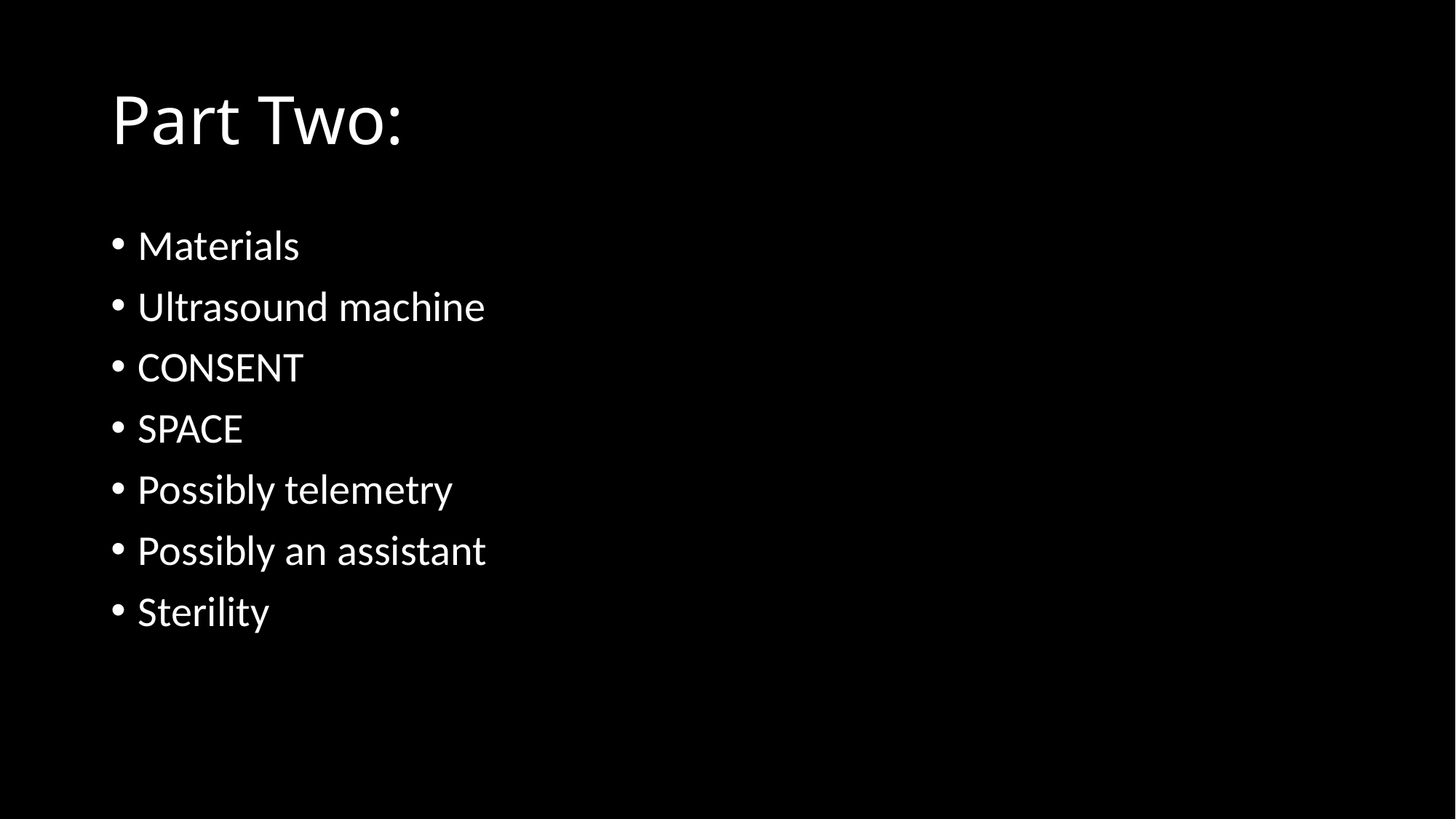

# Part Two:
Materials
Ultrasound machine
CONSENT
SPACE
Possibly telemetry
Possibly an assistant
Sterility

## Slide 16
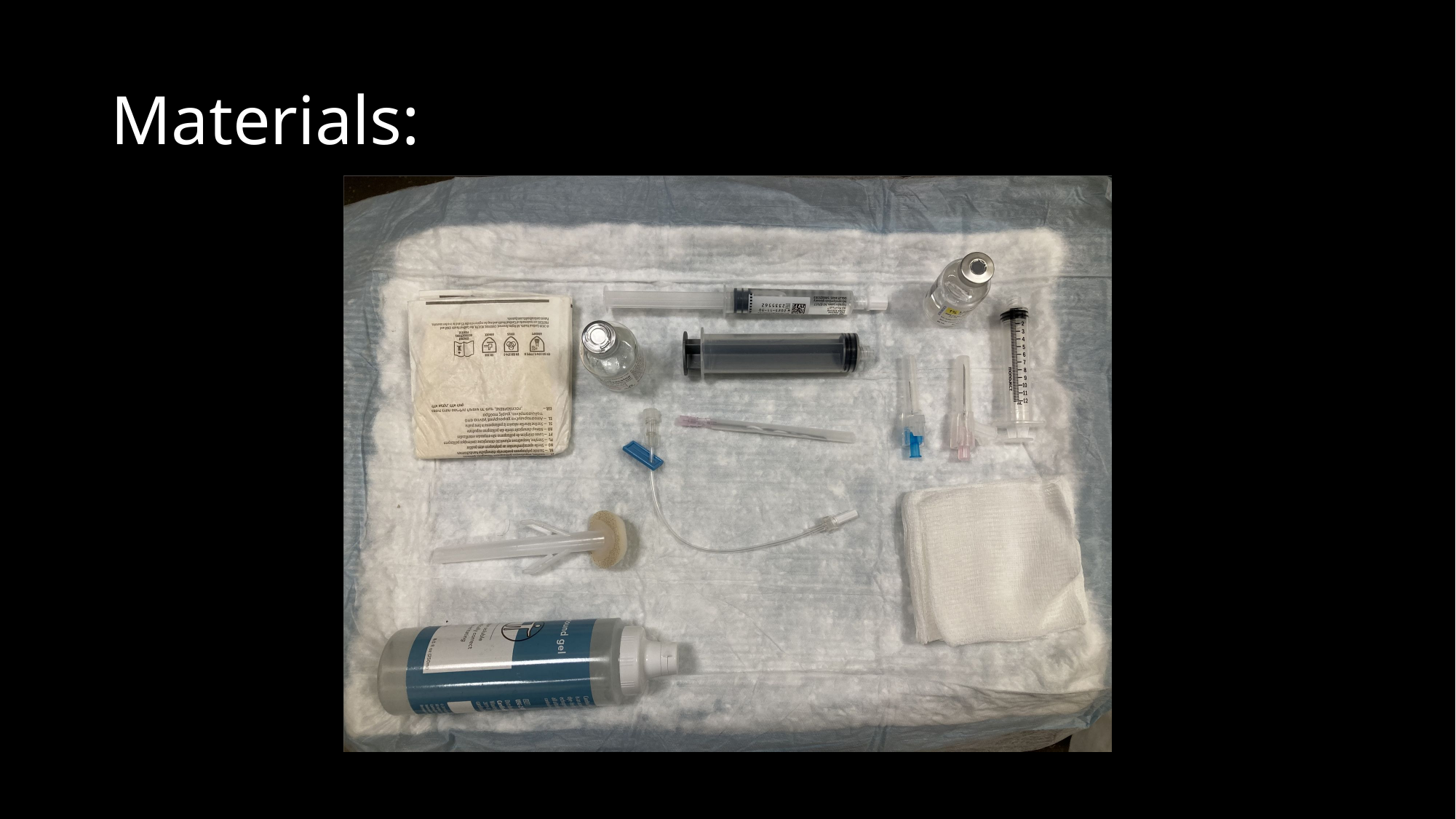

# Materials:

## Slide 17
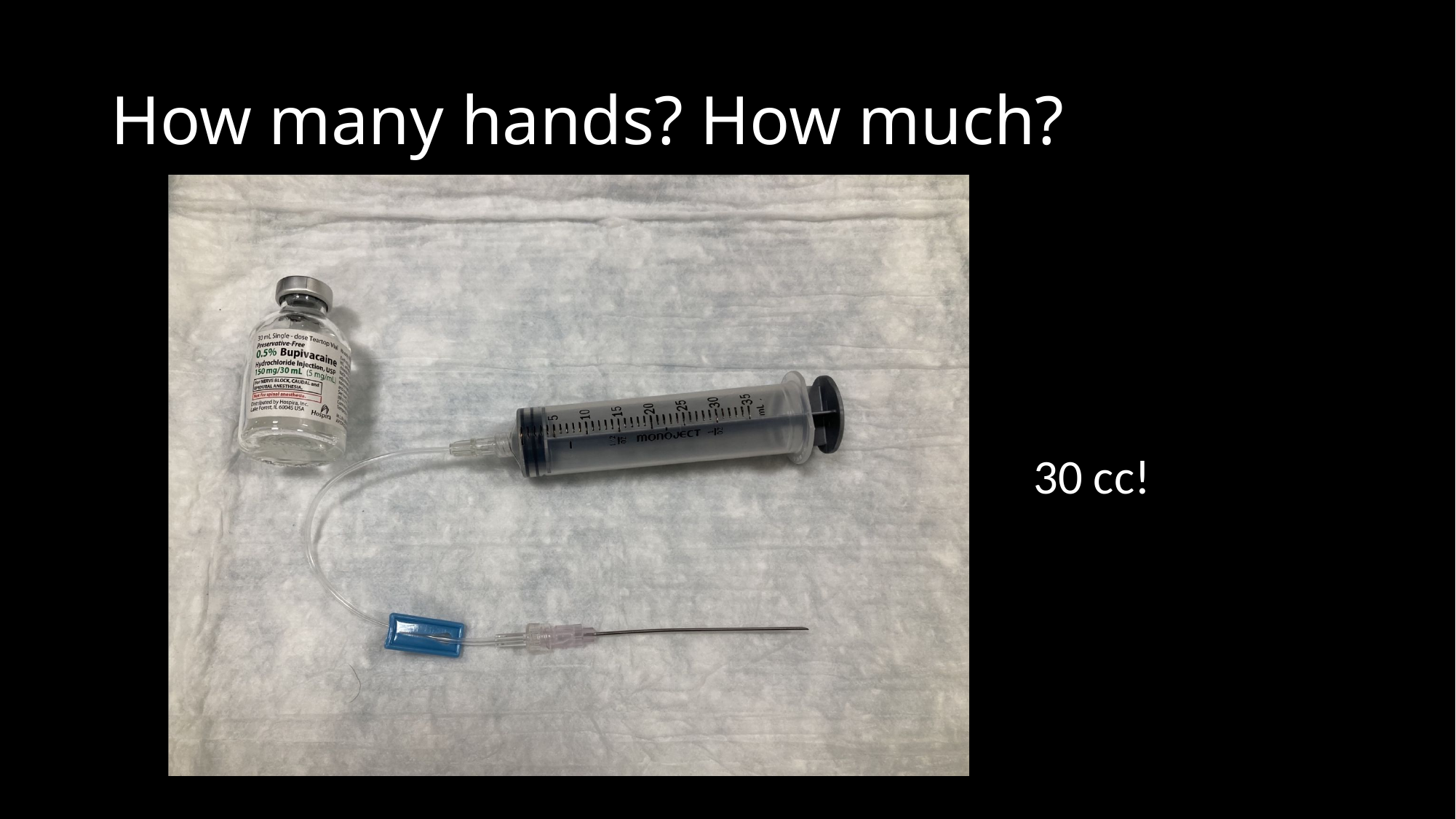

# How many hands? How much?
30 cc!

## Slide 18
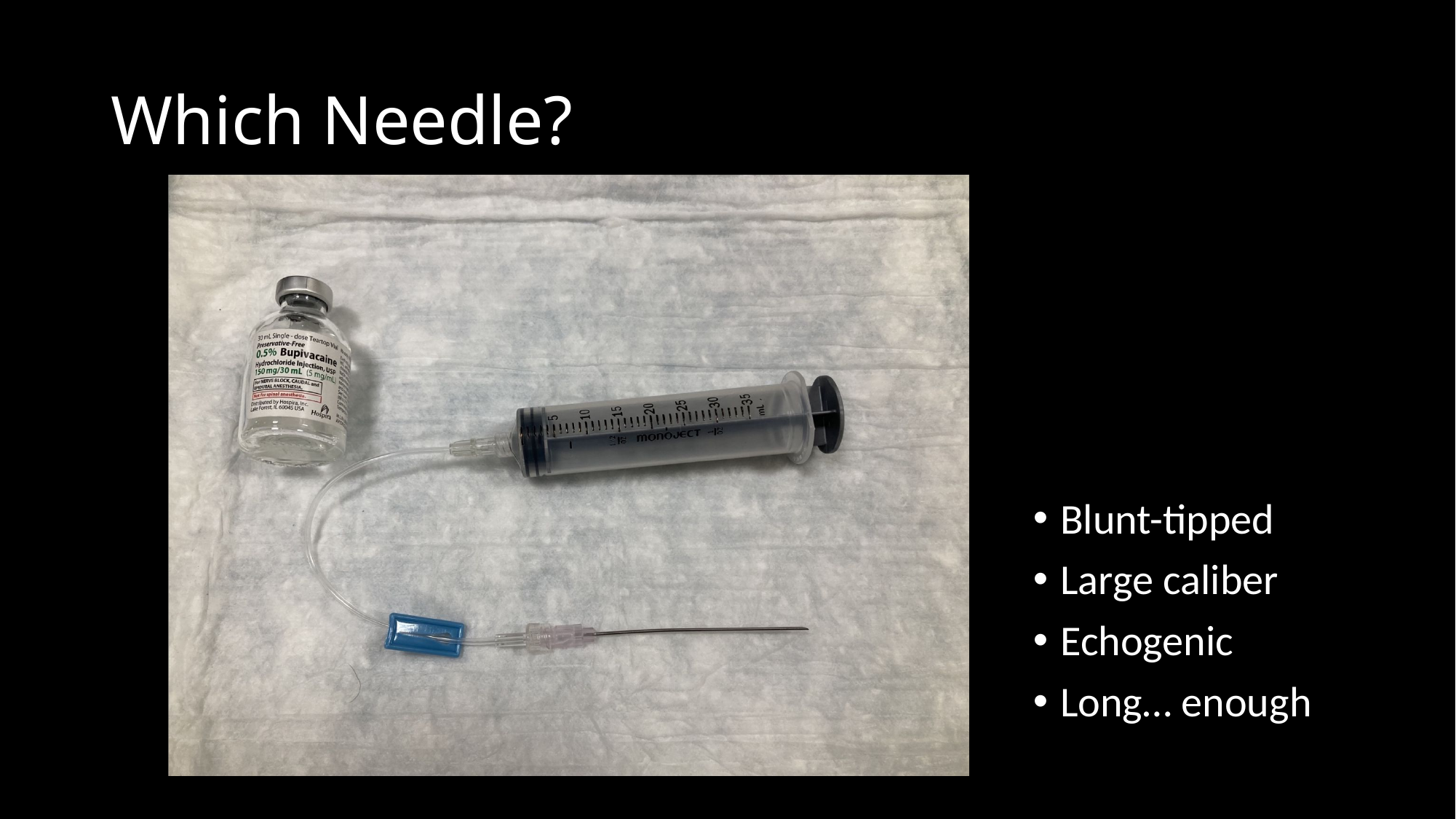

# Which Needle?
Blunt-tipped
Large caliber
Echogenic
Long… enough

## Slide 19
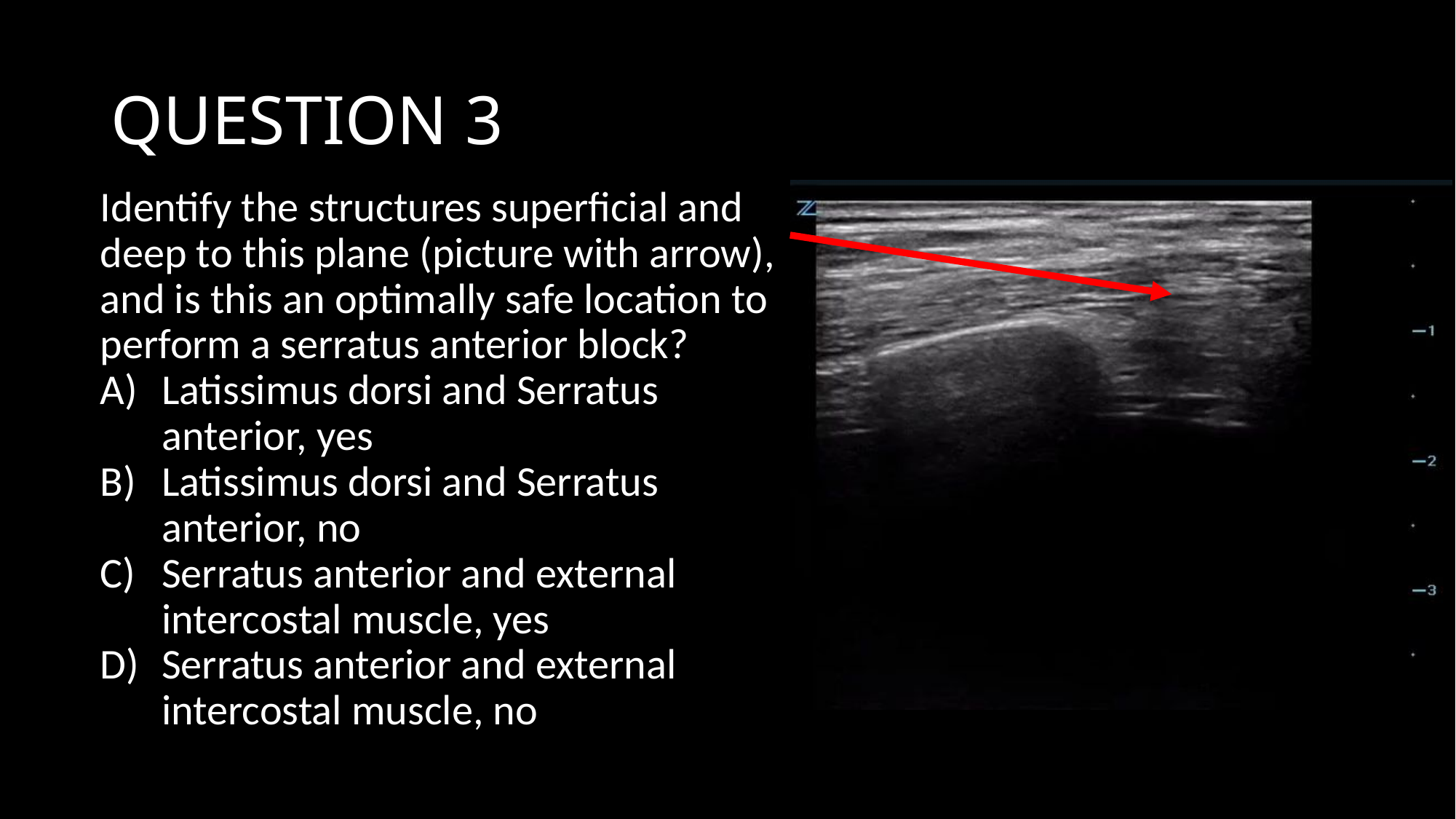

# QUESTION 3
Identify the structures superficial and deep to this plane (picture with arrow), and is this an optimally safe location to perform a serratus anterior block?
Latissimus dorsi and Serratus anterior, yes
Latissimus dorsi and Serratus anterior, no
Serratus anterior and external intercostal muscle, yes
Serratus anterior and external intercostal muscle, no

## Slide 20
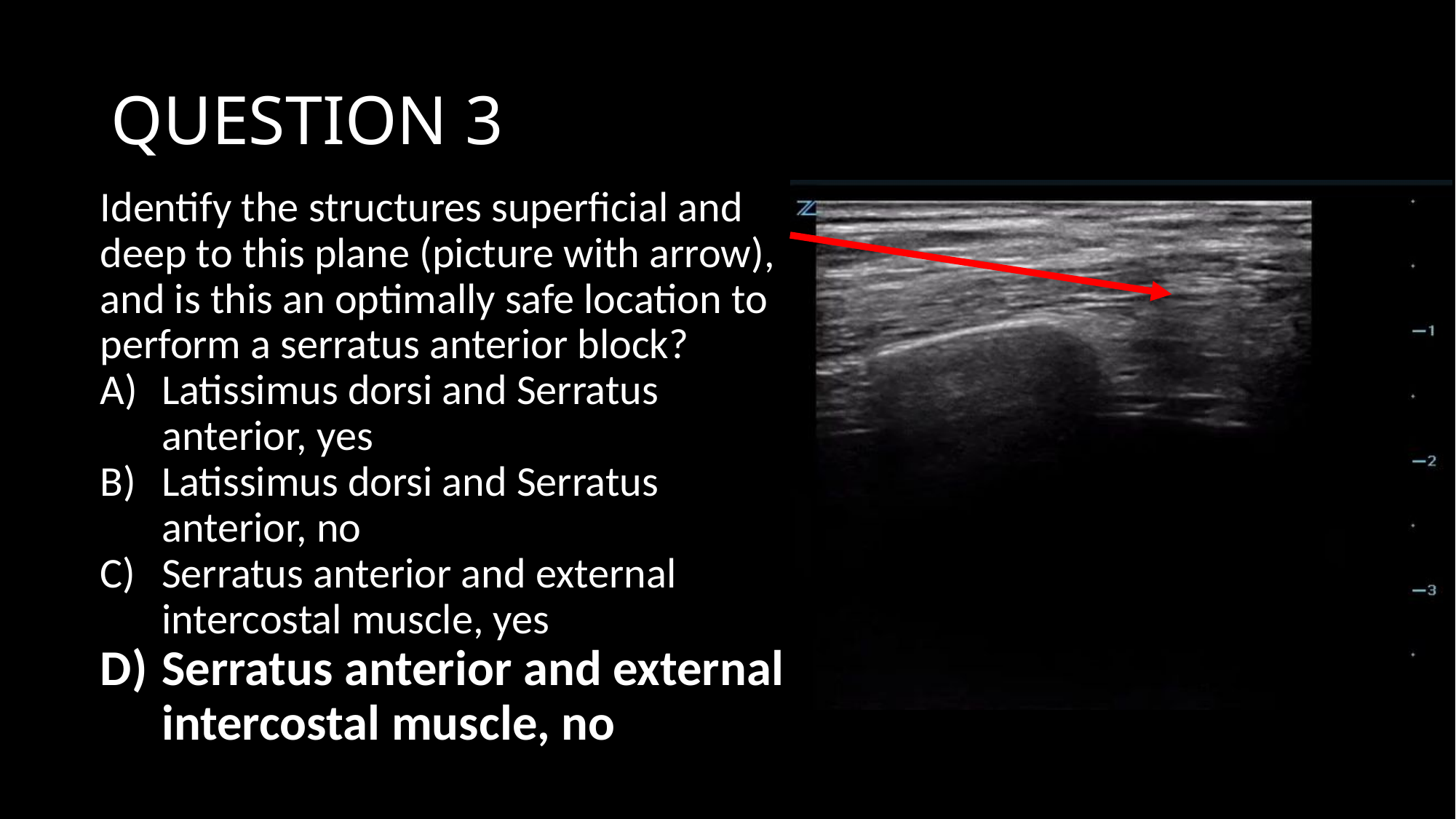

# QUESTION 3
Identify the structures superficial and deep to this plane (picture with arrow), and is this an optimally safe location to perform a serratus anterior block?
Latissimus dorsi and Serratus anterior, yes
Latissimus dorsi and Serratus anterior, no
Serratus anterior and external intercostal muscle, yes
Serratus anterior and external intercostal muscle, no

## Slide 21
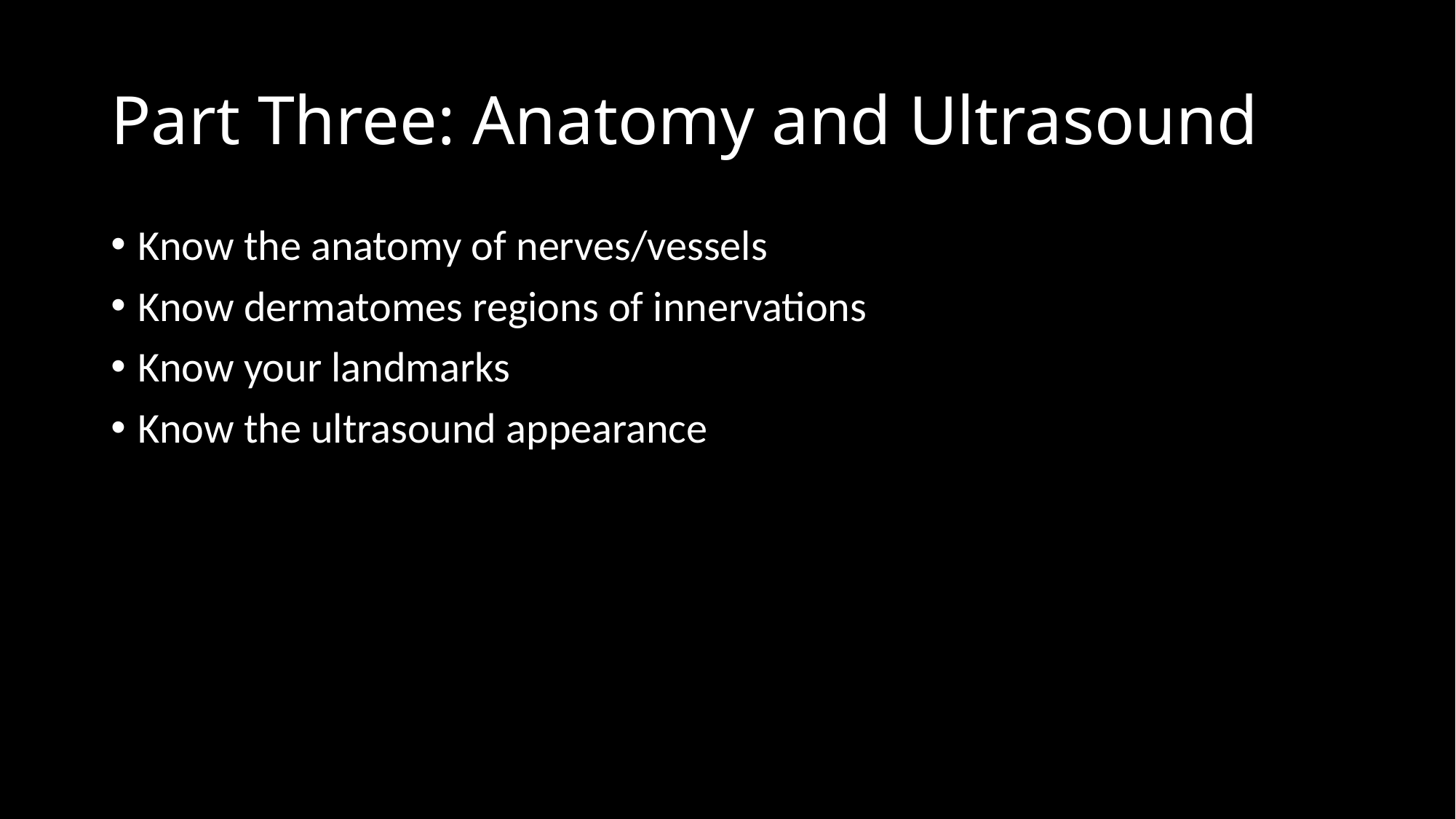

# Part Three: Anatomy and Ultrasound
Know the anatomy of nerves/vessels
Know dermatomes regions of innervations
Know your landmarks
Know the ultrasound appearance

## Slide 22
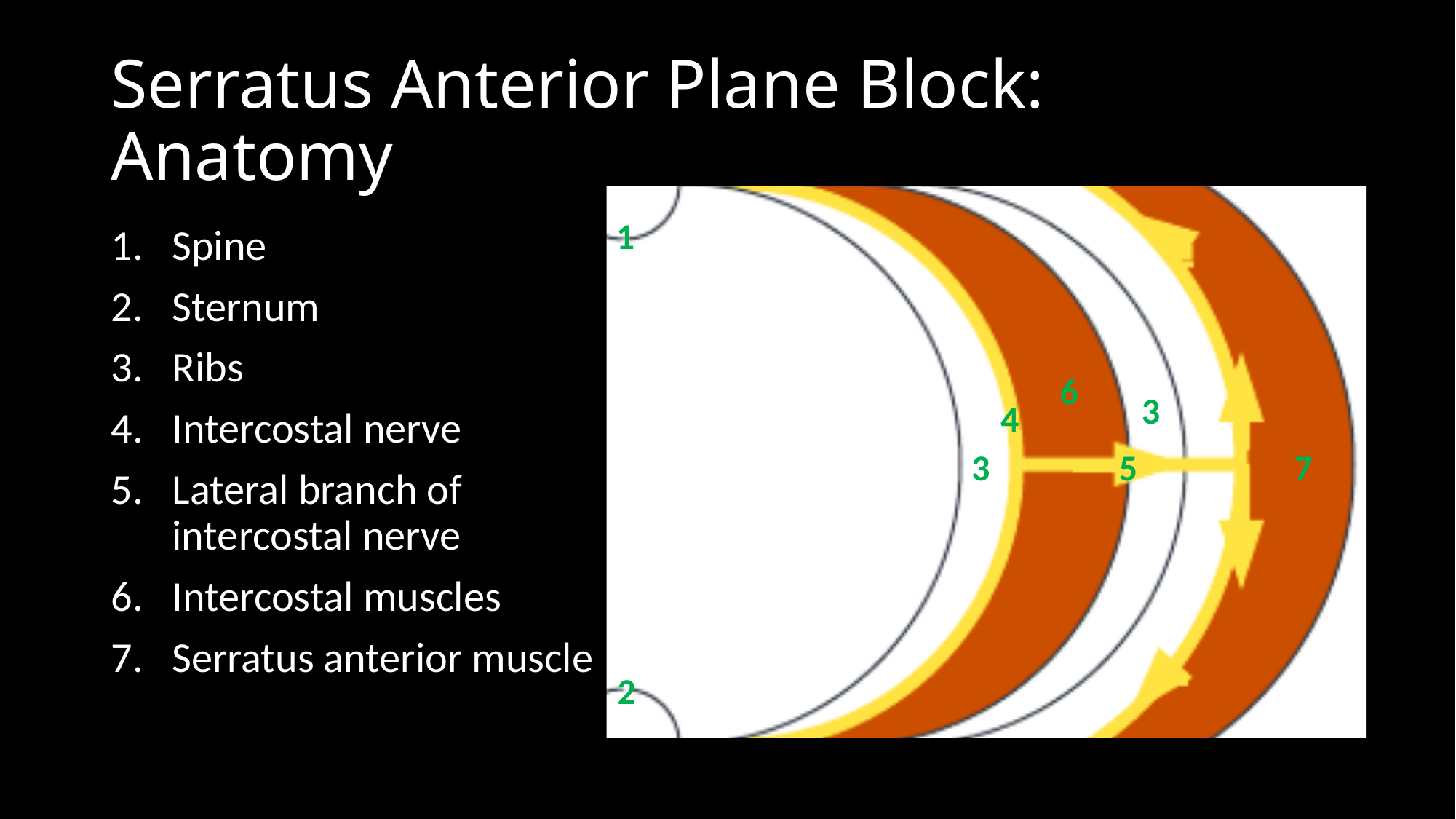

# Serratus Anterior Plane Block: Anatomy
1
Spine
Sternum
Ribs
Intercostal nerve
Lateral branch of intercostal nerve
Intercostal muscles
Serratus anterior muscle
6
3
4
3
5
7
2

## Slide 23
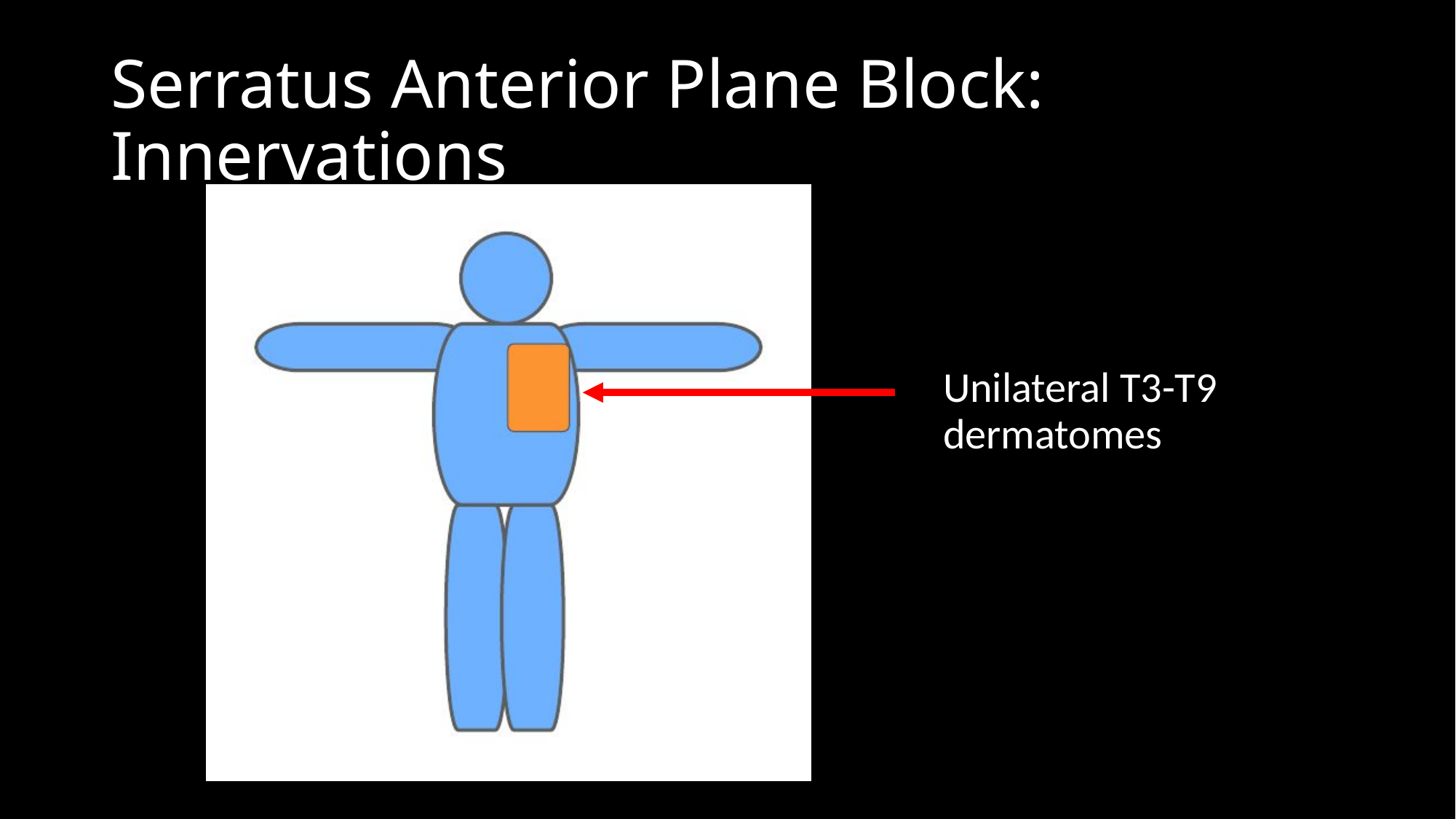

# Serratus Anterior Plane Block: Innervations
Unilateral T3-T9 dermatomes

## Slide 24
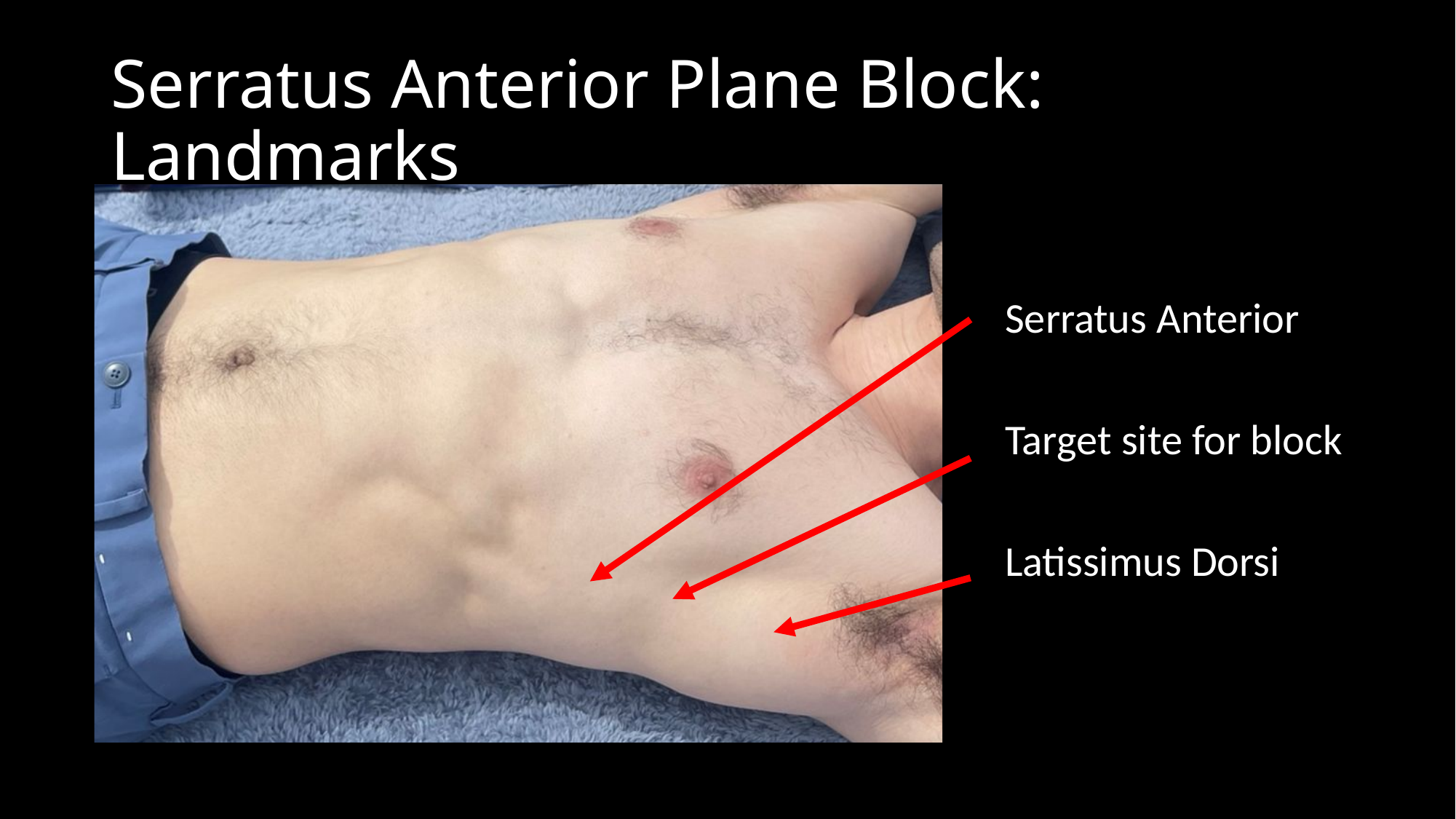

# Serratus Anterior Plane Block: Landmarks
Serratus Anterior
Target site for block
Latissimus Dorsi

## Slide 25
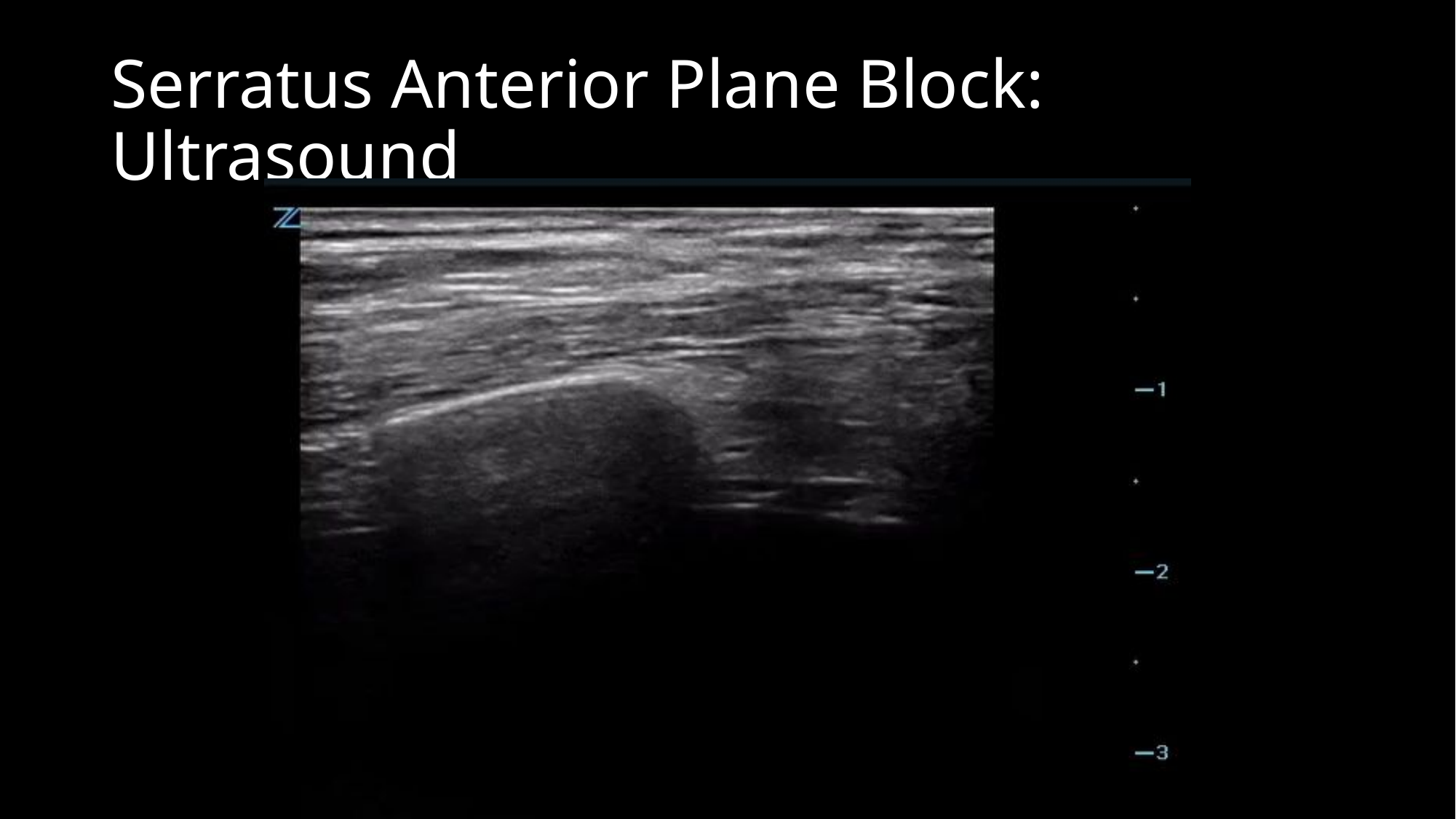

# Serratus Anterior Plane Block: Ultrasound

## Slide 26
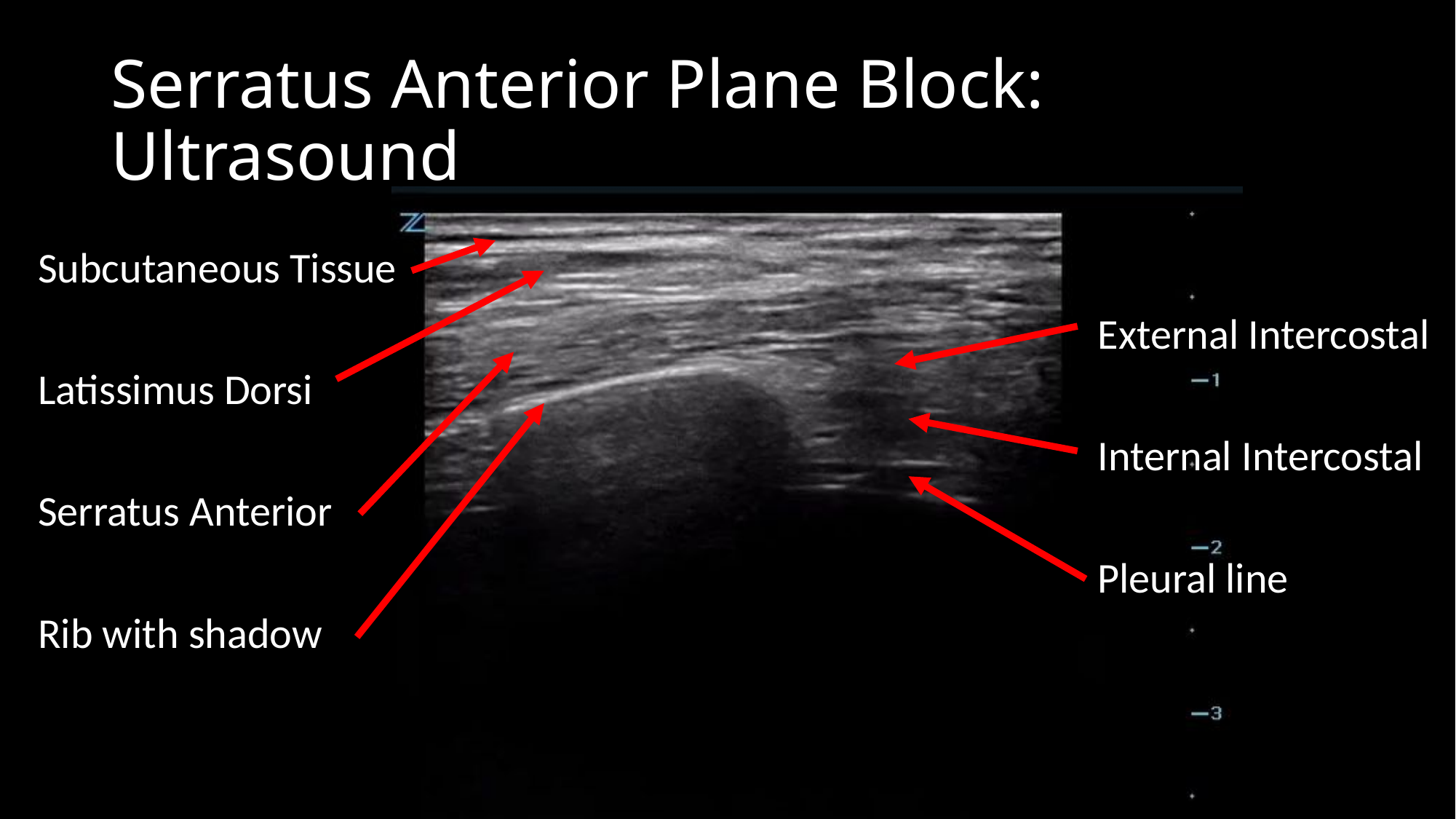

# Serratus Anterior Plane Block: Ultrasound
Subcutaneous Tissue
Latissimus Dorsi
Serratus Anterior
Rib with shadow
External Intercostal
Internal Intercostal
Pleural line

## Slide 27
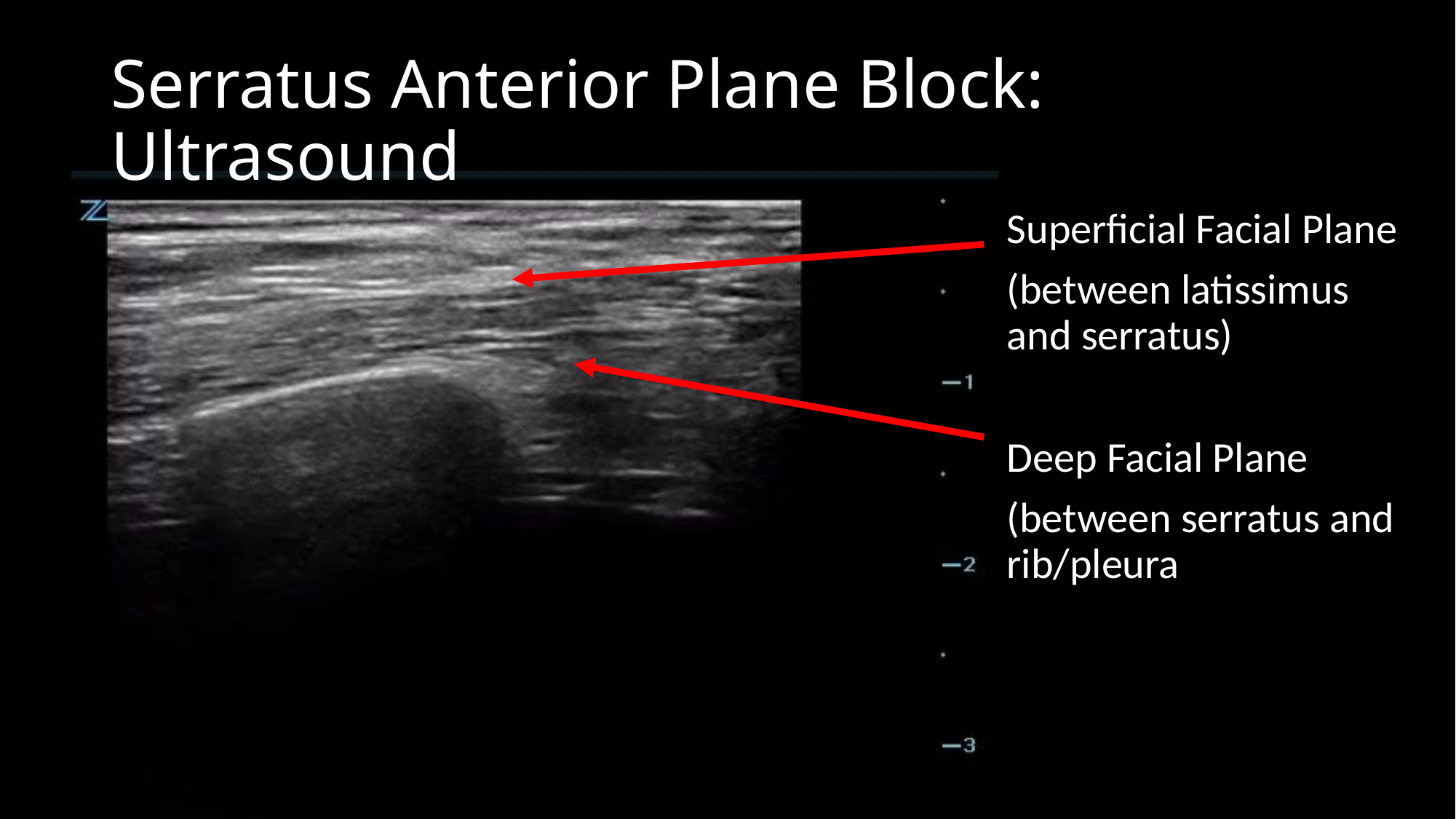

# Serratus Anterior Plane Block: Ultrasound
Superficial Facial Plane
(between latissimus and serratus)
Deep Facial Plane
(between serratus and rib/pleura

## Slide 28
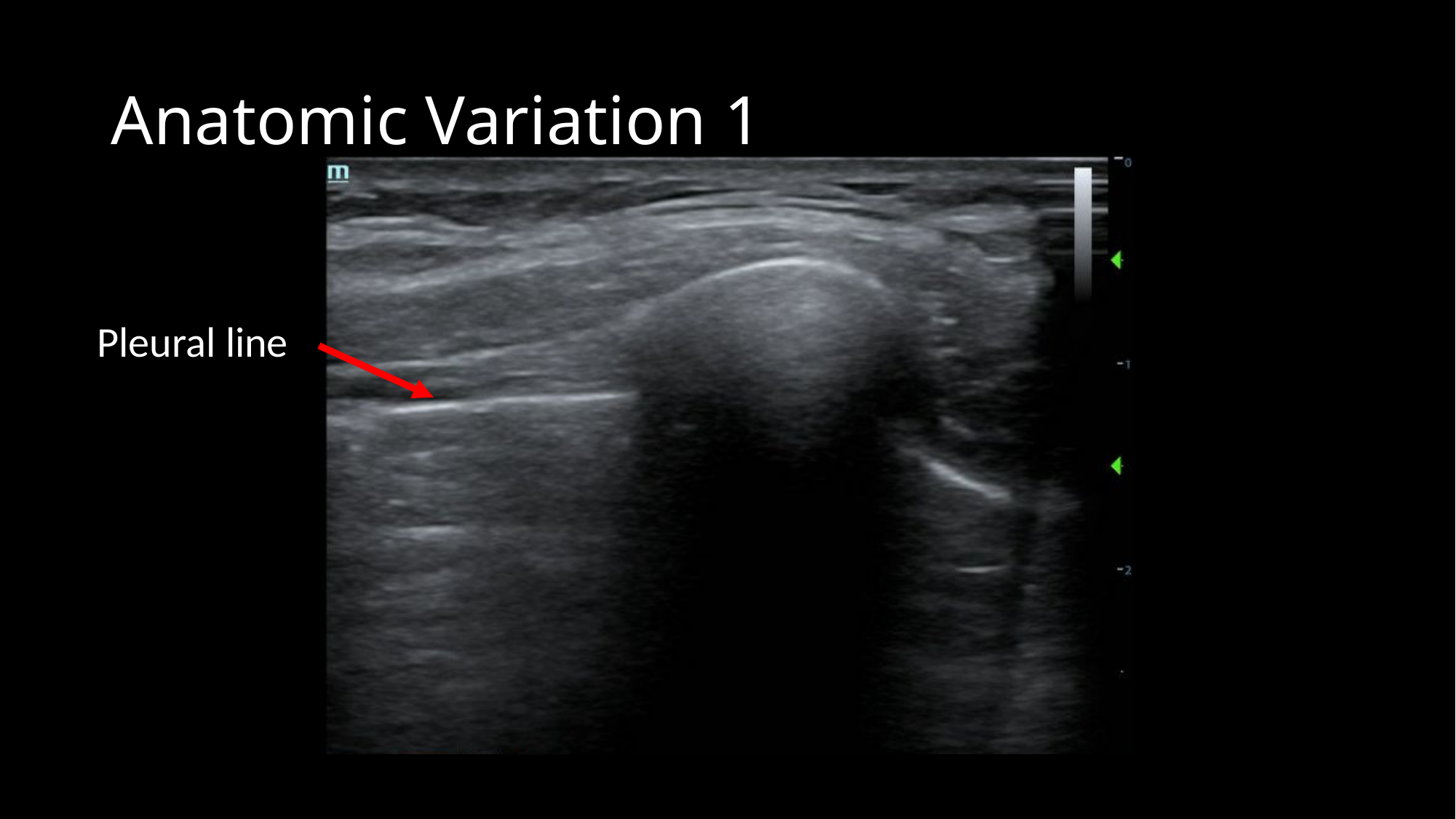

# Anatomic Variation 1
Pleural line

## Slide 29
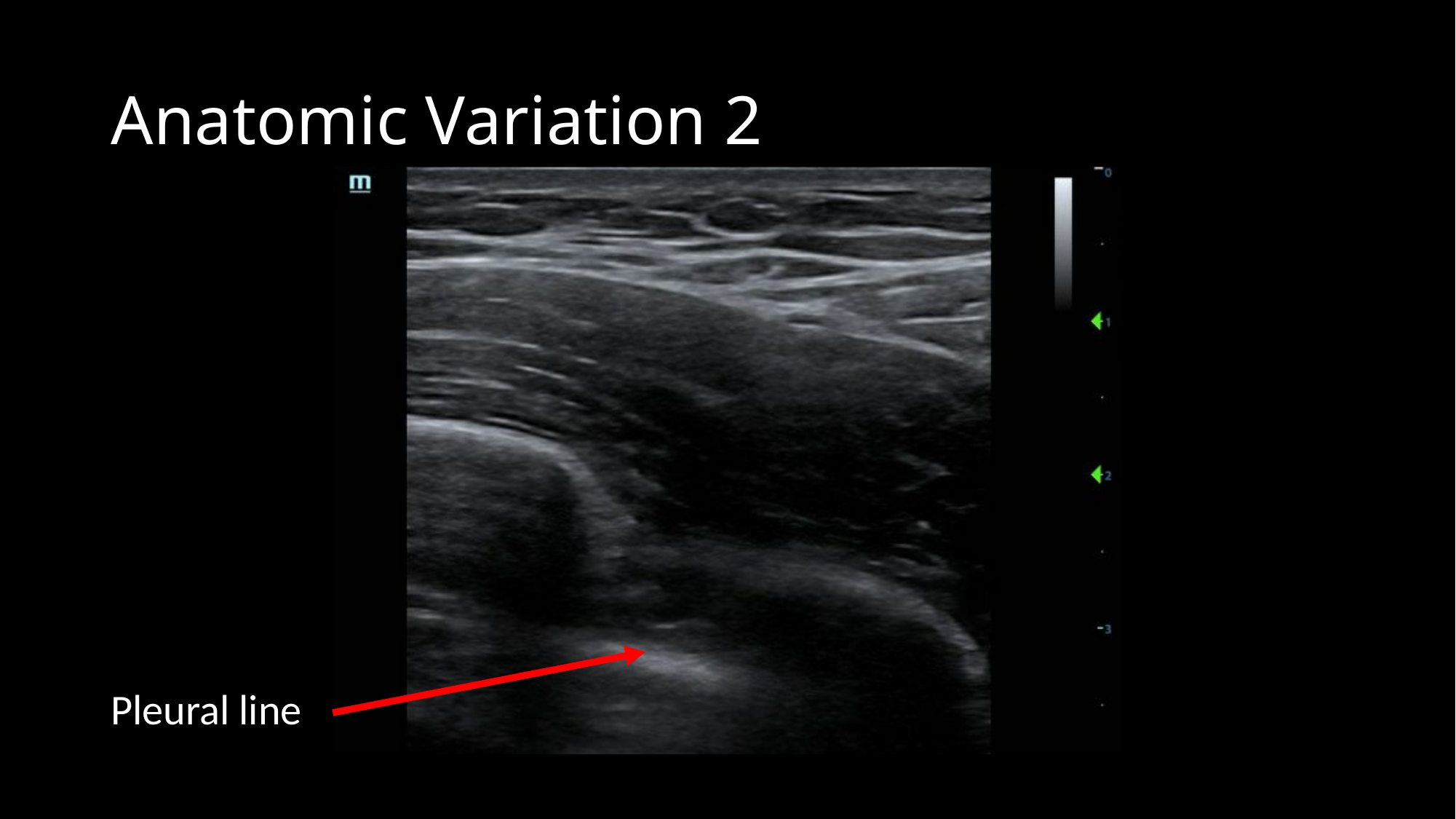

# Anatomic Variation 2
Pleural line

## Slide 30
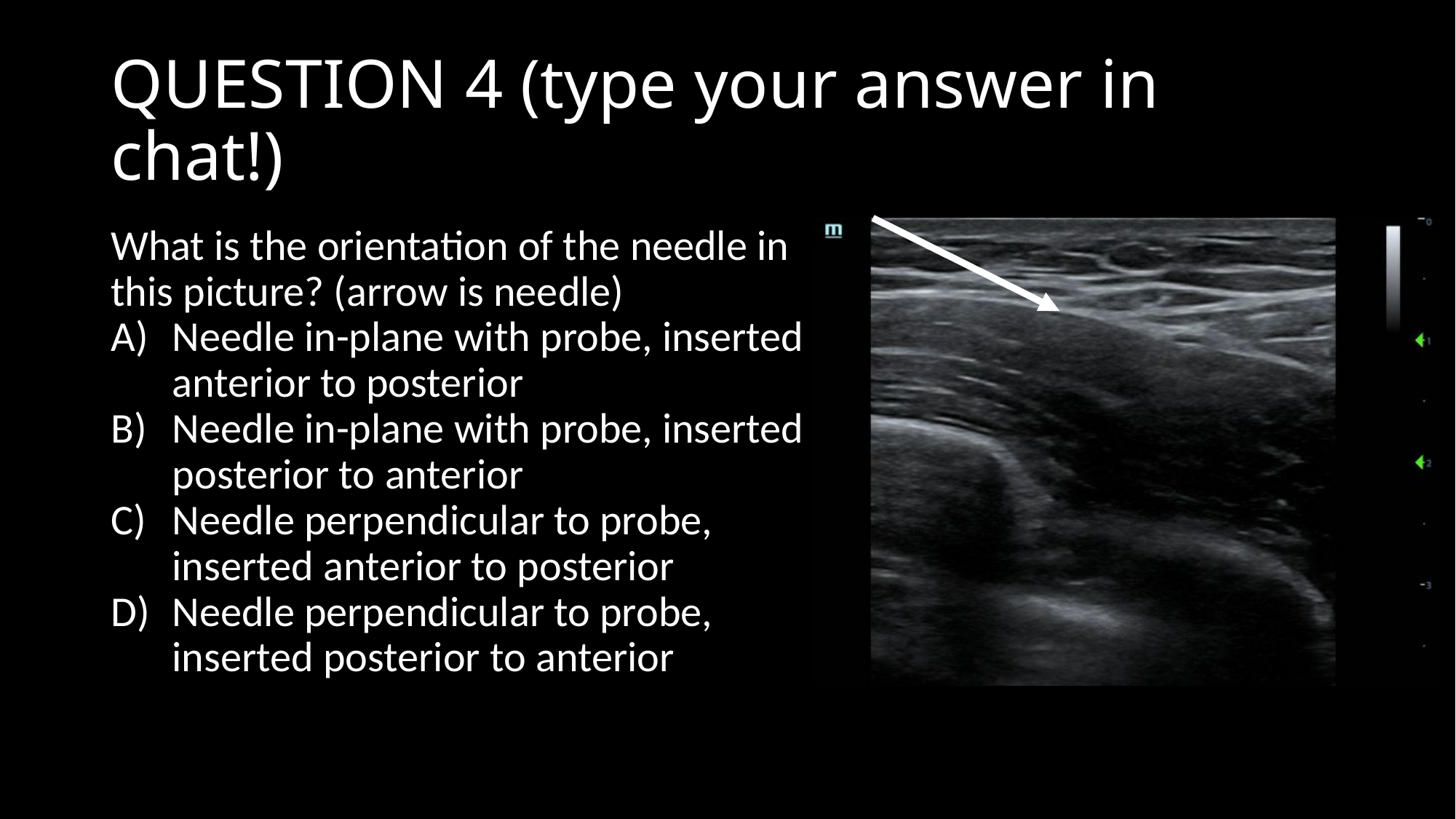

# QUESTION 4 (type your answer in chat!)
What is the orientation of the needle in this picture? (arrow is needle)
Needle in-plane with probe, inserted anterior to posterior
Needle in-plane with probe, inserted posterior to anterior
Needle perpendicular to probe, inserted anterior to posterior
Needle perpendicular to probe, inserted posterior to anterior

## Slide 31
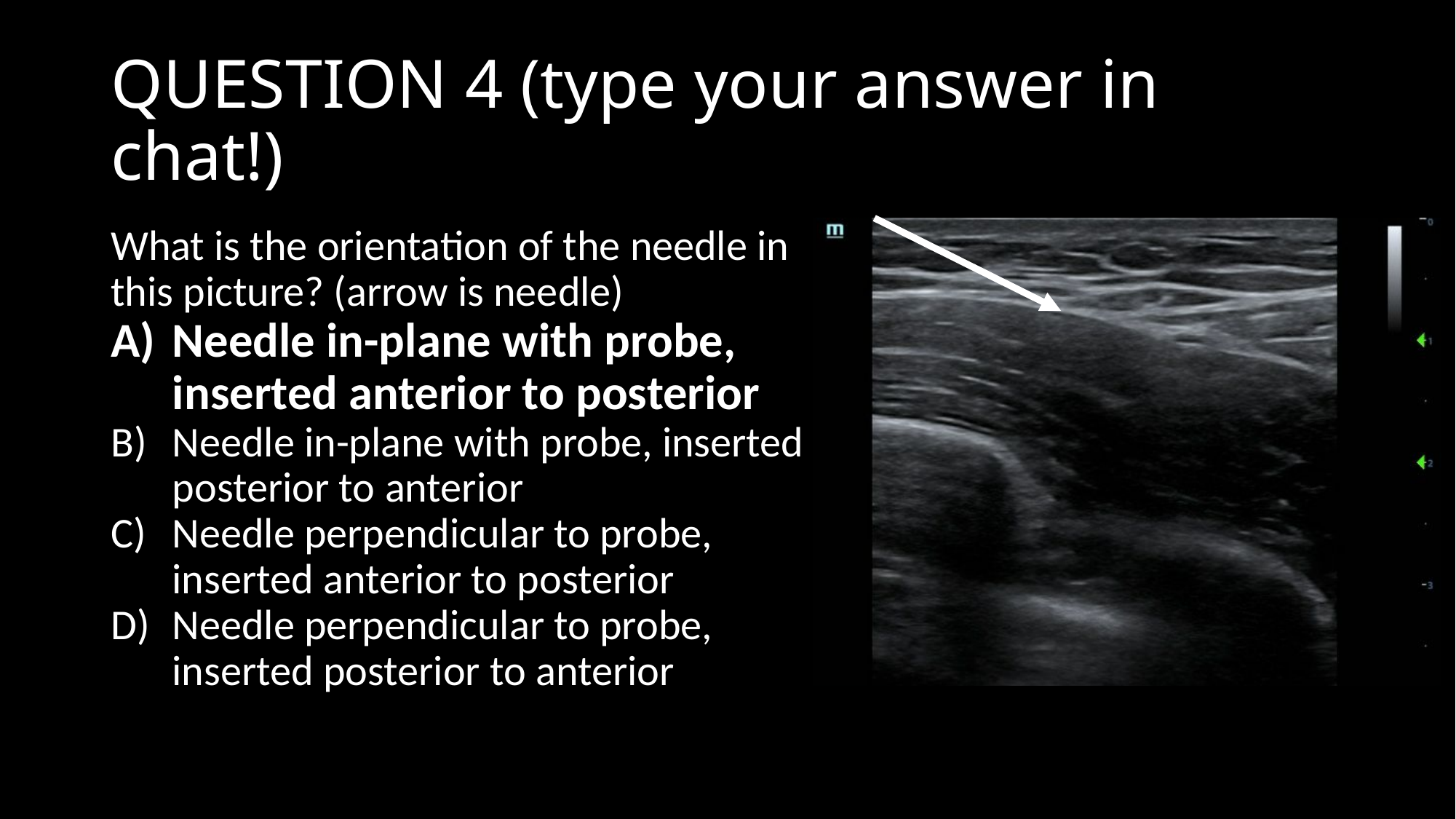

# QUESTION 4 (type your answer in chat!)
What is the orientation of the needle in this picture? (arrow is needle)
Needle in-plane with probe, inserted anterior to posterior
Needle in-plane with probe, inserted posterior to anterior
Needle perpendicular to probe, inserted anterior to posterior
Needle perpendicular to probe, inserted posterior to anterior

## Slide 32
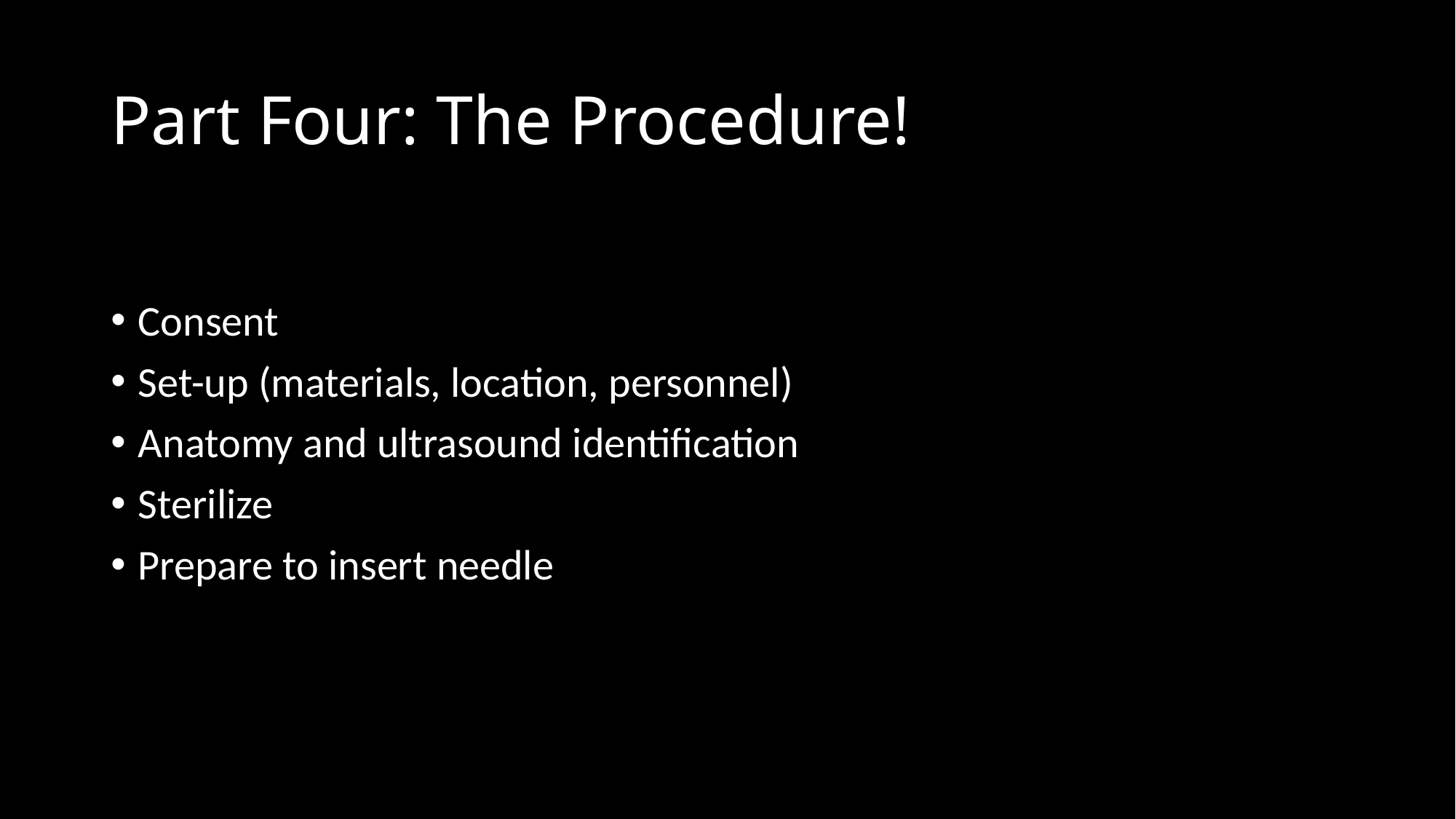

# Part Four: The Procedure!
Consent
Set-up (materials, location, personnel)
Anatomy and ultrasound identification
Sterilize
Prepare to insert needle

## Slide 33
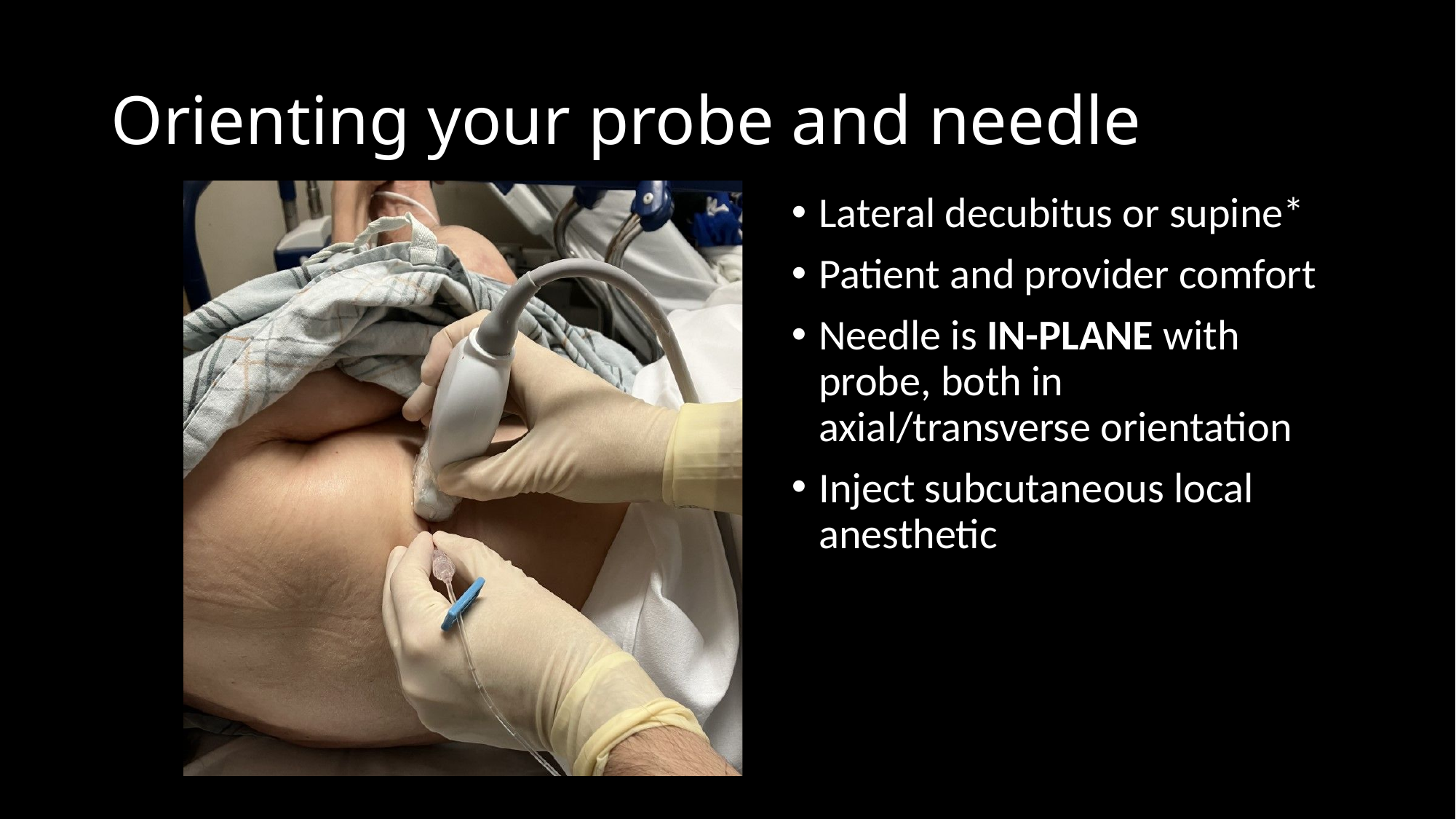

# Orienting your probe and needle
Lateral decubitus or supine*
Patient and provider comfort
Needle is IN-PLANE with probe, both in axial/transverse orientation
Inject subcutaneous local anesthetic

## Slide 34
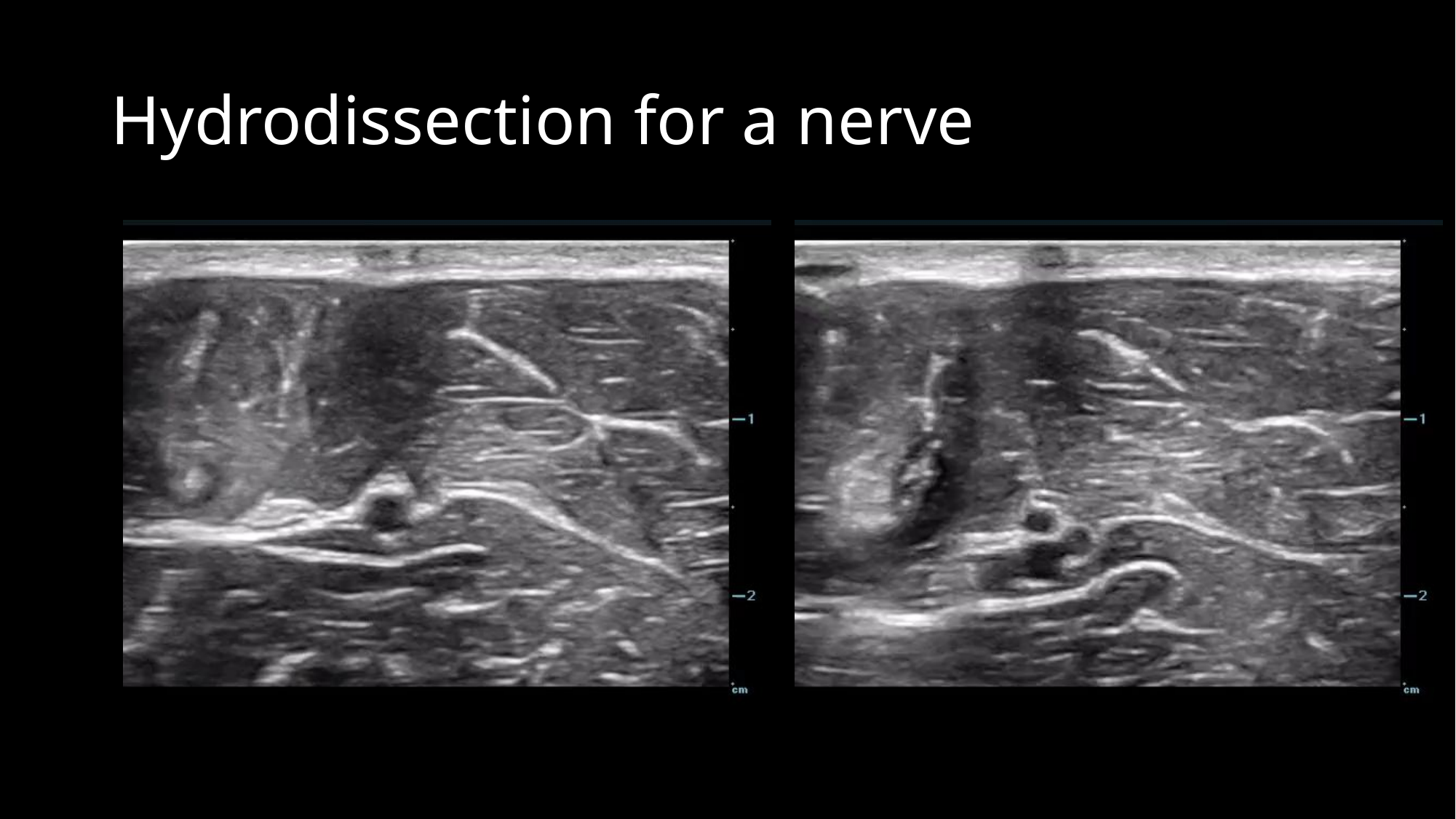

# Hydrodissection for a nerve

## Slide 35
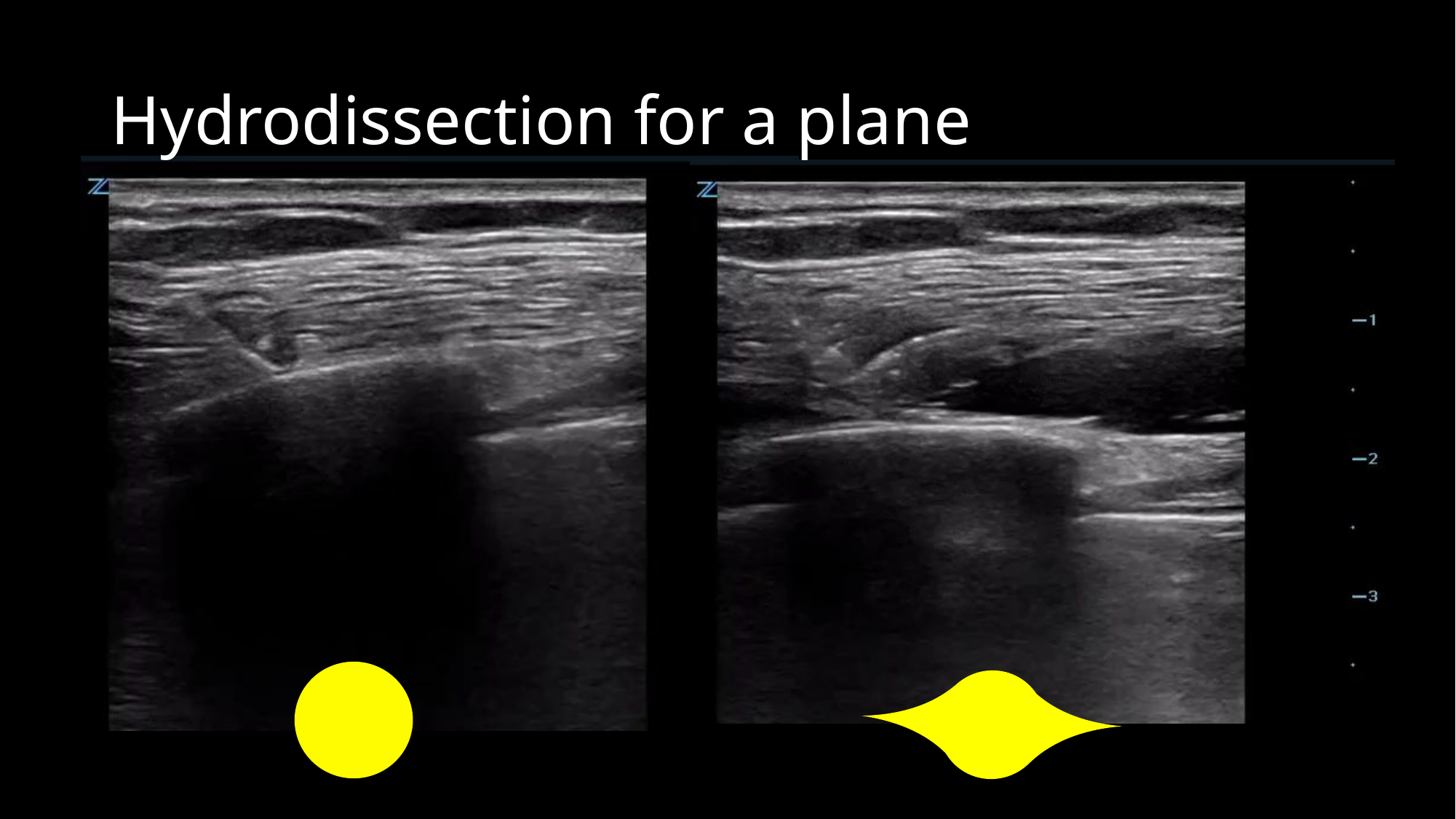

# Hydrodissection for a plane

## Slide 36
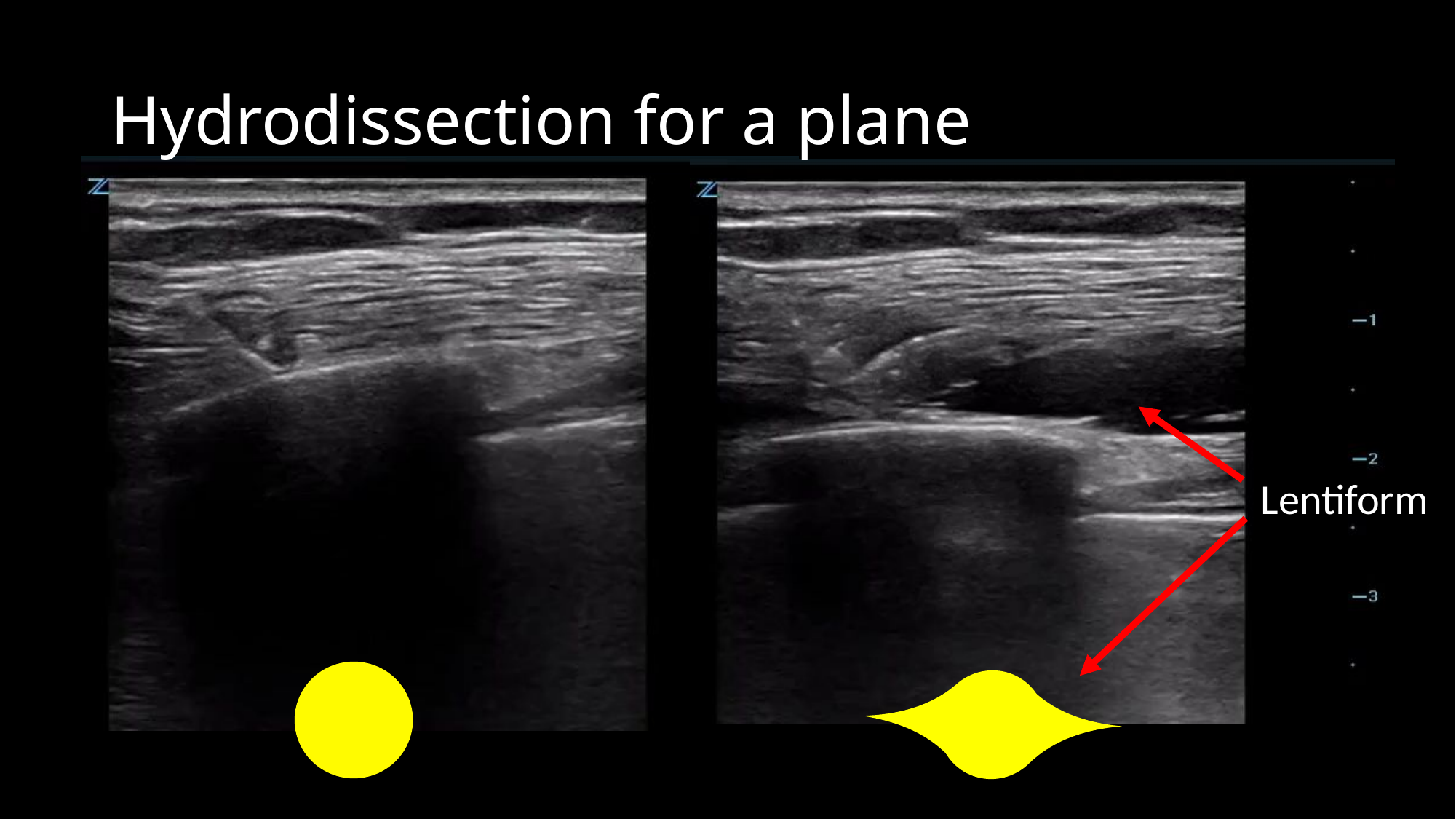

# Hydrodissection for a plane
Lentiform

## Slide 37
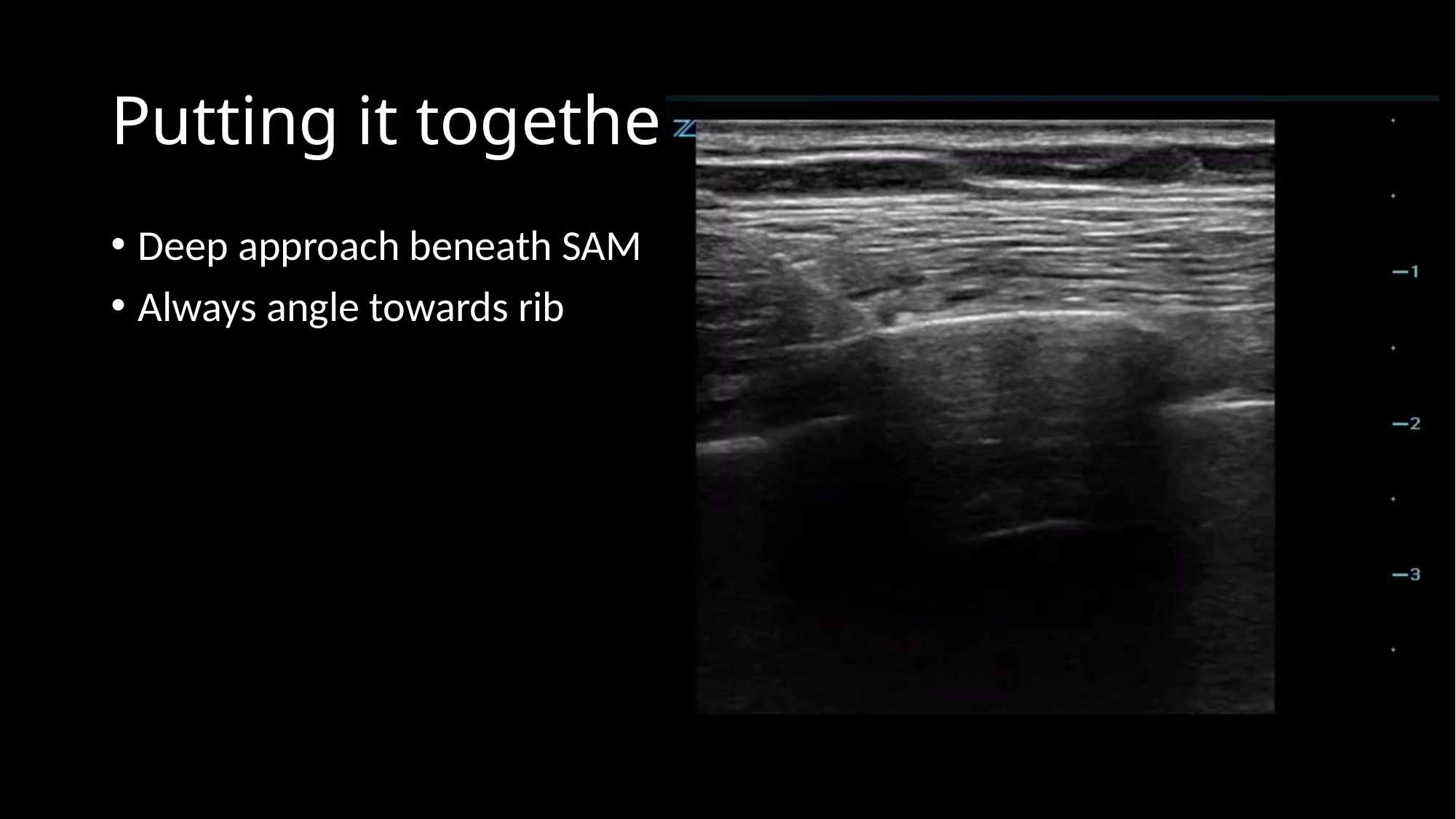

# Putting it together
Deep approach beneath SAM
Always angle towards rib

## Slide 38
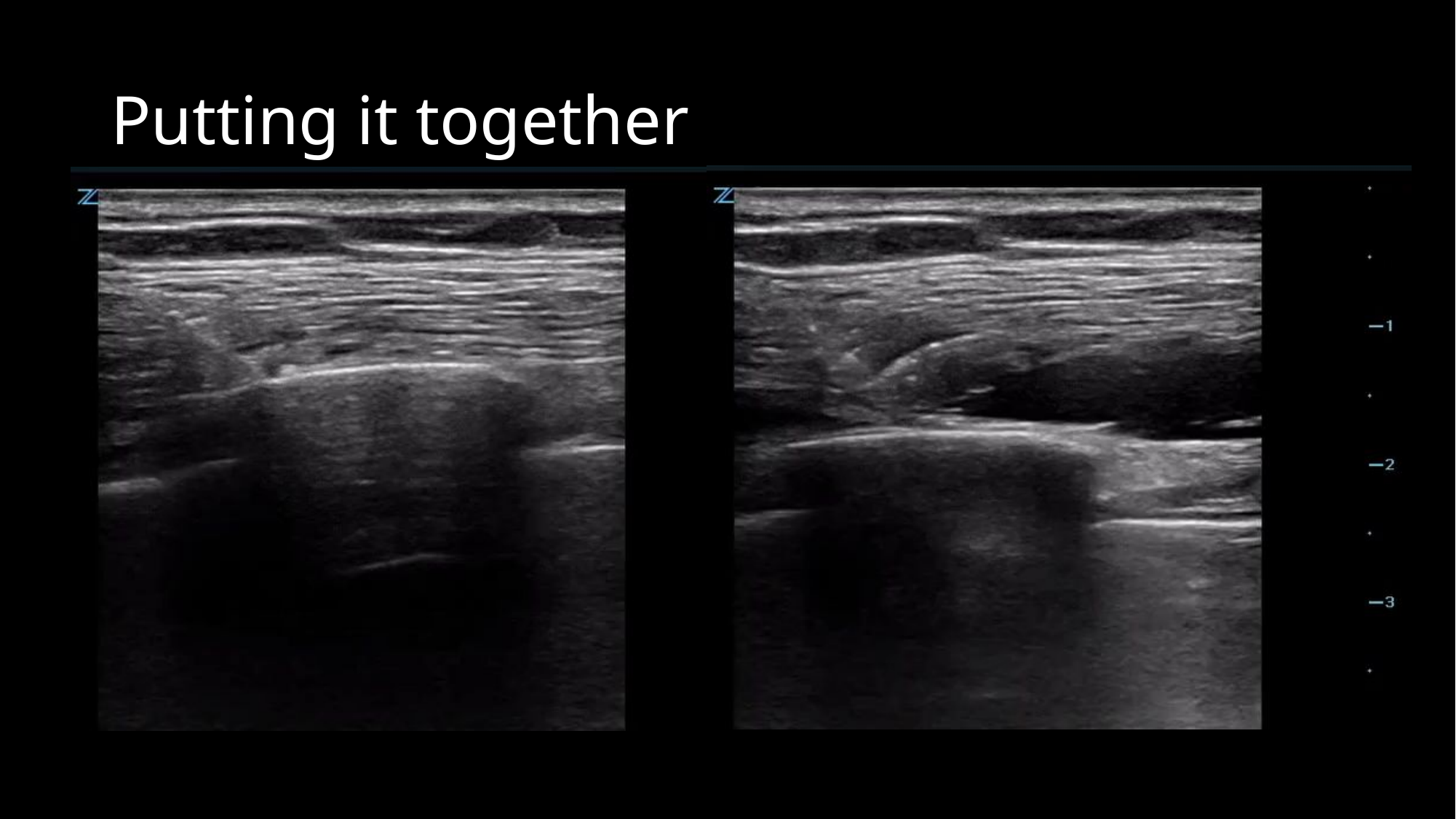

# Putting it together

## Slide 39
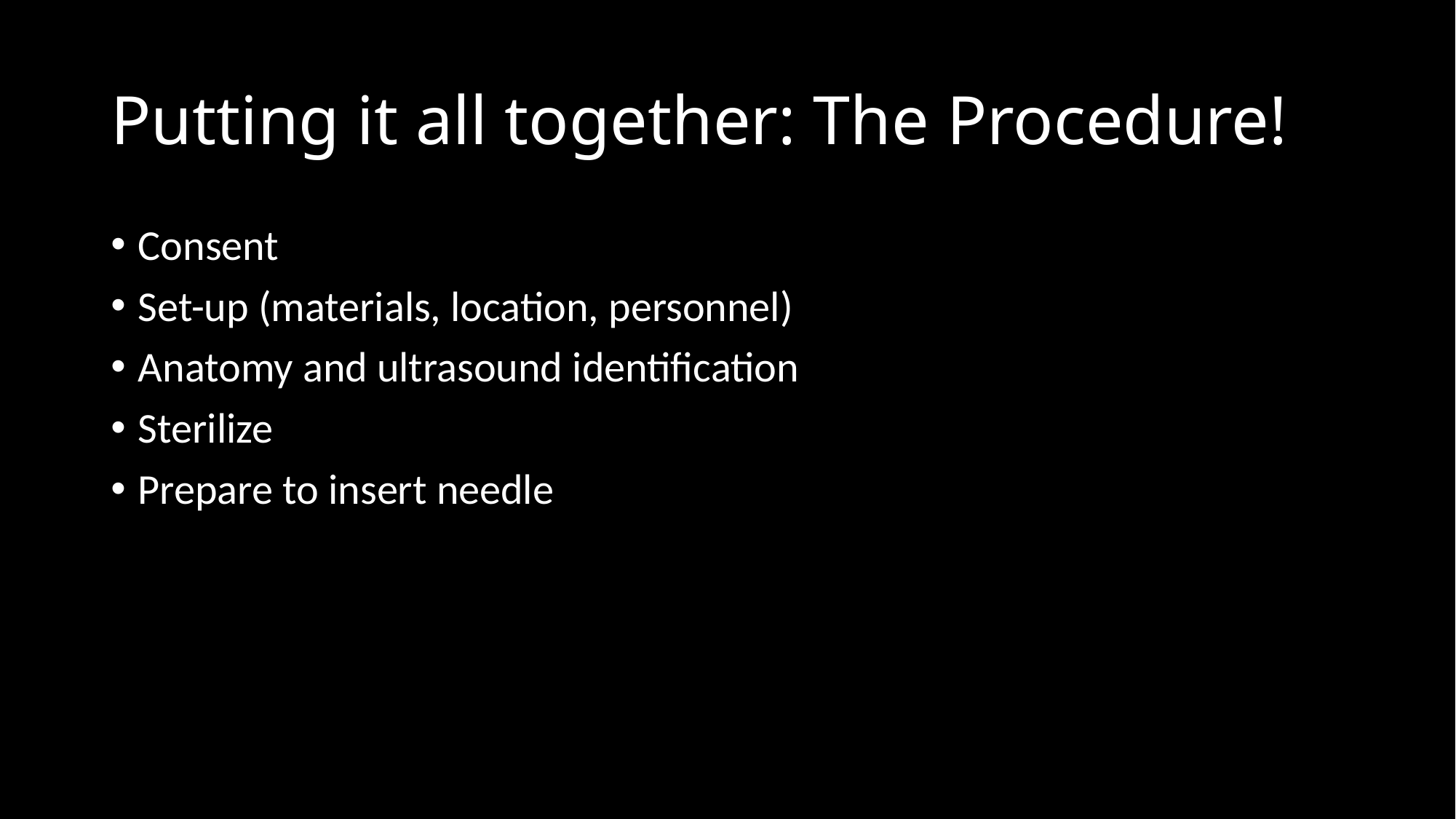

# Putting it all together: The Procedure!
Consent
Set-up (materials, location, personnel)
Anatomy and ultrasound identification
Sterilize
Prepare to insert needle

## Slide 40
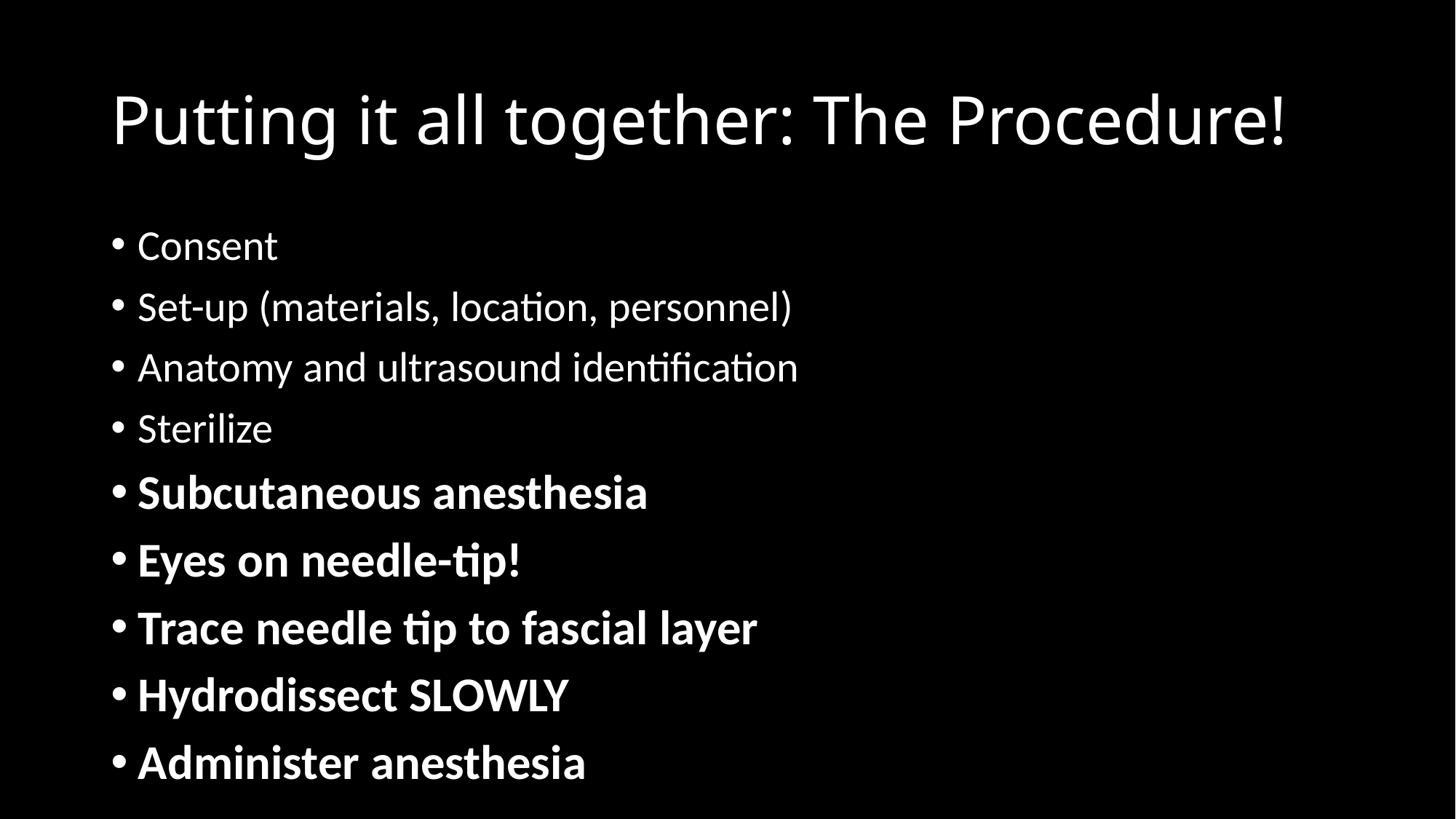

# Putting it all together: The Procedure!
Consent
Set-up (materials, location, personnel)
Anatomy and ultrasound identification
Sterilize
Subcutaneous anesthesia
Eyes on needle-tip!
Trace needle tip to fascial layer
Hydrodissect SLOWLY
Administer anesthesia

## Slide 41
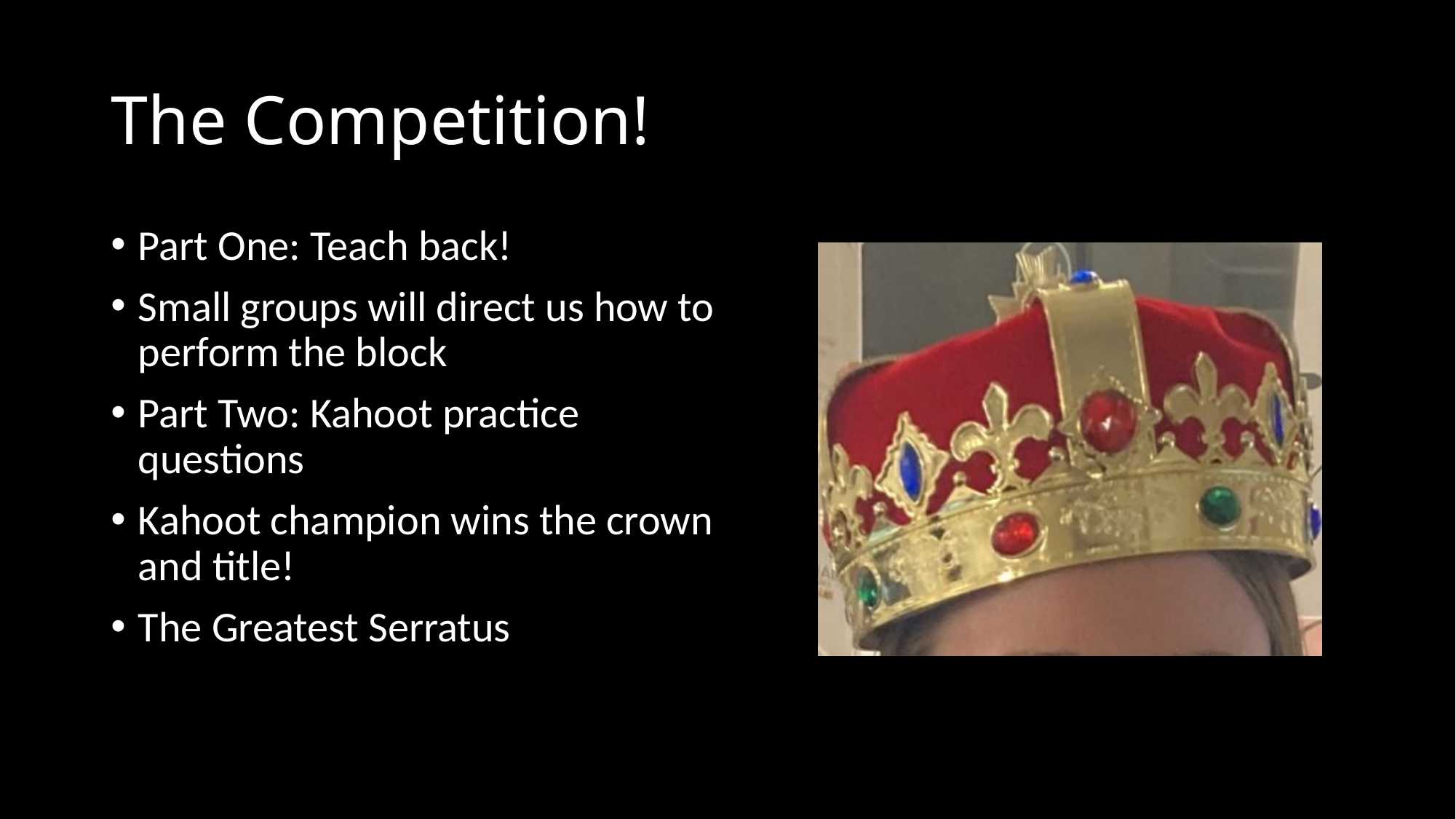

# The Competition!
Part One: Teach back!
Small groups will direct us how to perform the block
Part Two: Kahoot practice questions
Kahoot champion wins the crown and title!
The Greatest Serratus

## Slide 42
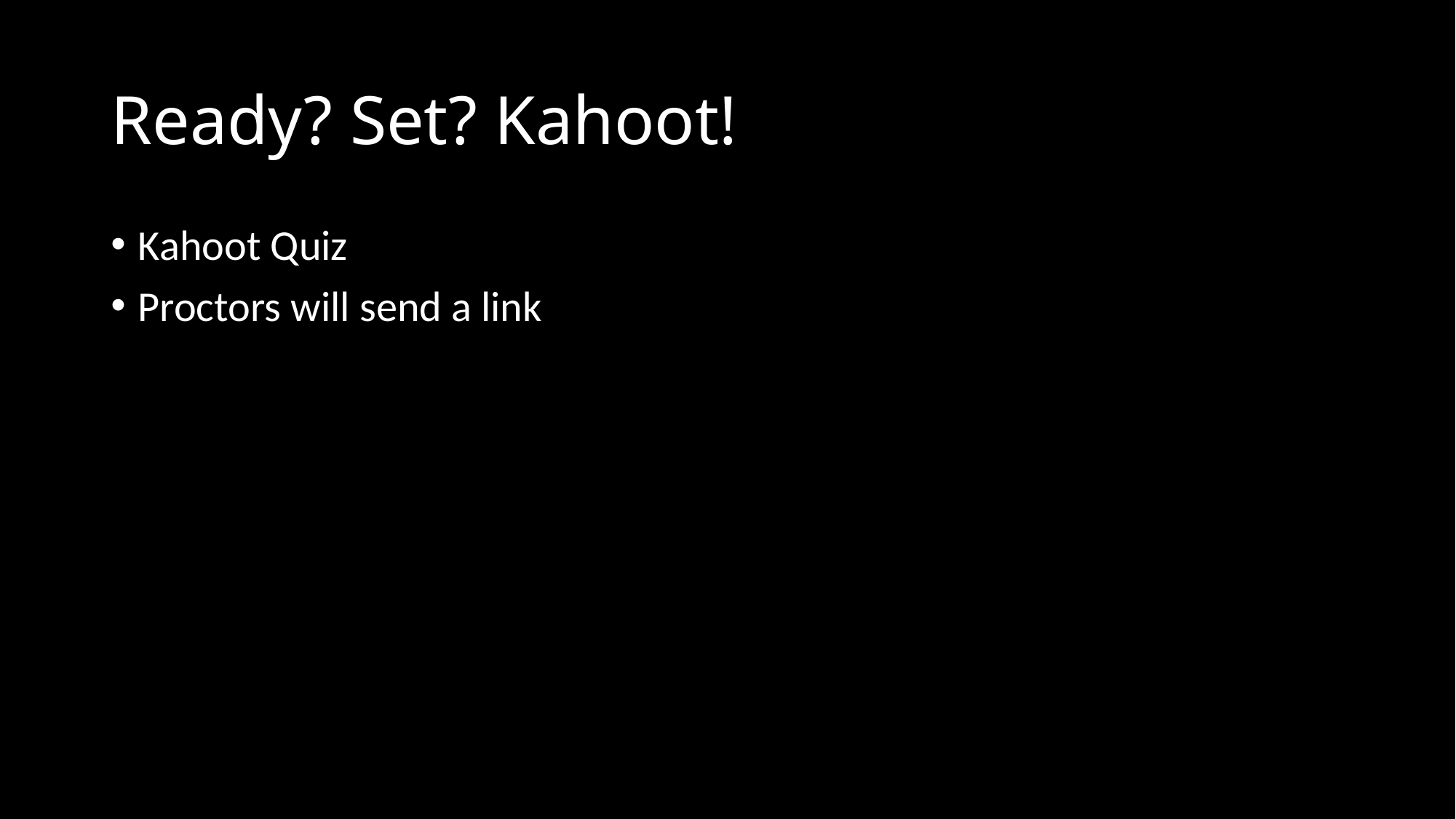

# Ready? Set? Kahoot!
Kahoot Quiz
Proctors will send a link

## Slide 43
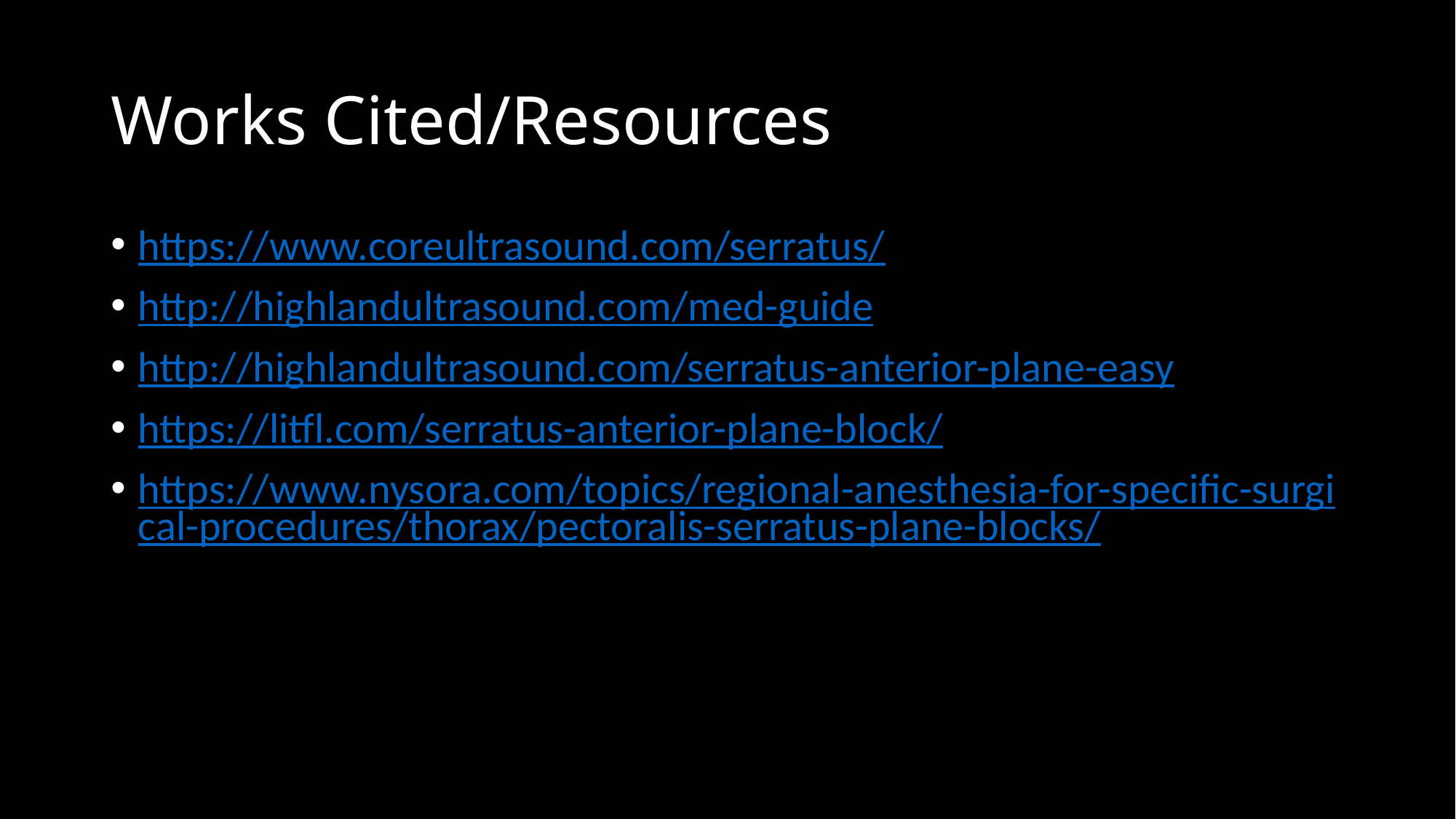

# Works Cited/Resources
https://www.coreultrasound.com/serratus/
http://highlandultrasound.com/med-guide
http://highlandultrasound.com/serratus-anterior-plane-easy
https://litfl.com/serratus-anterior-plane-block/
https://www.nysora.com/topics/regional-anesthesia-for-specific-surgical-procedures/thorax/pectoralis-serratus-plane-blocks/

## Slide 44
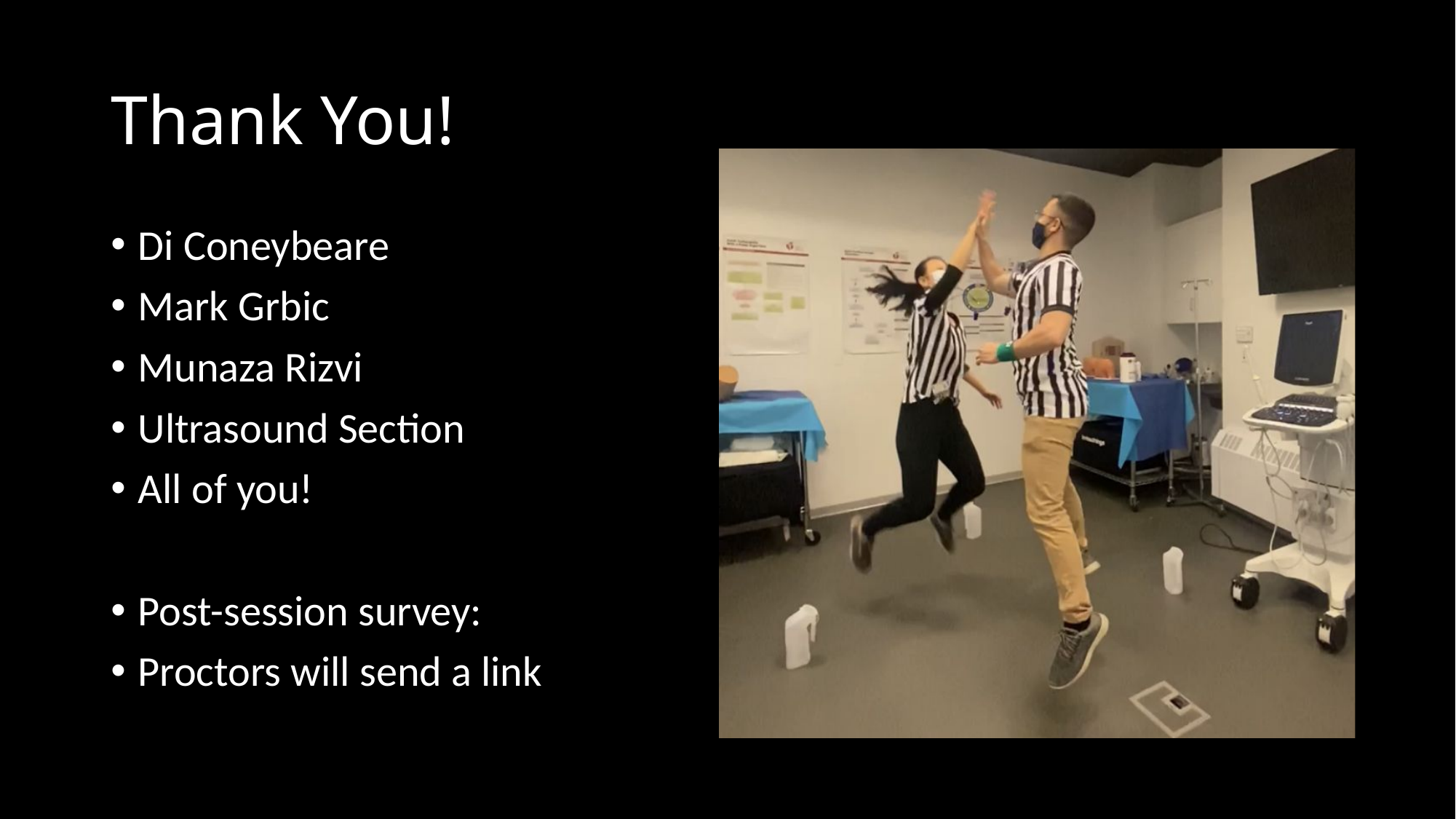

# Thank You!
Di Coneybeare
Mark Grbic
Munaza Rizvi
Ultrasound Section
All of you!
Post-session survey:
Proctors will send a link
