# Supplementary material for: Serratus Anterior Plane Block Remote Learning Curriculum
Source: MedEdPORTAL. 2024 Oct 25;20:11454. doi: 10.15766/mep_2374-8265.11454 (PMC11502517; doi:10.15766/mep_2374-8265.11454)
Supplement: Supplementary file 1 — Serratus Anterior Block Presentation.pptxSAPB Kahoot Quiz.pptxSAPB Proctor Instructions.docxQualtrics Presession Survey.docxQualtrics Postsession Survey.docx [file mep_2374-8265.11454-s001.zip › B. SAPB Kahoot Quiz.pptx]

## Slide 1
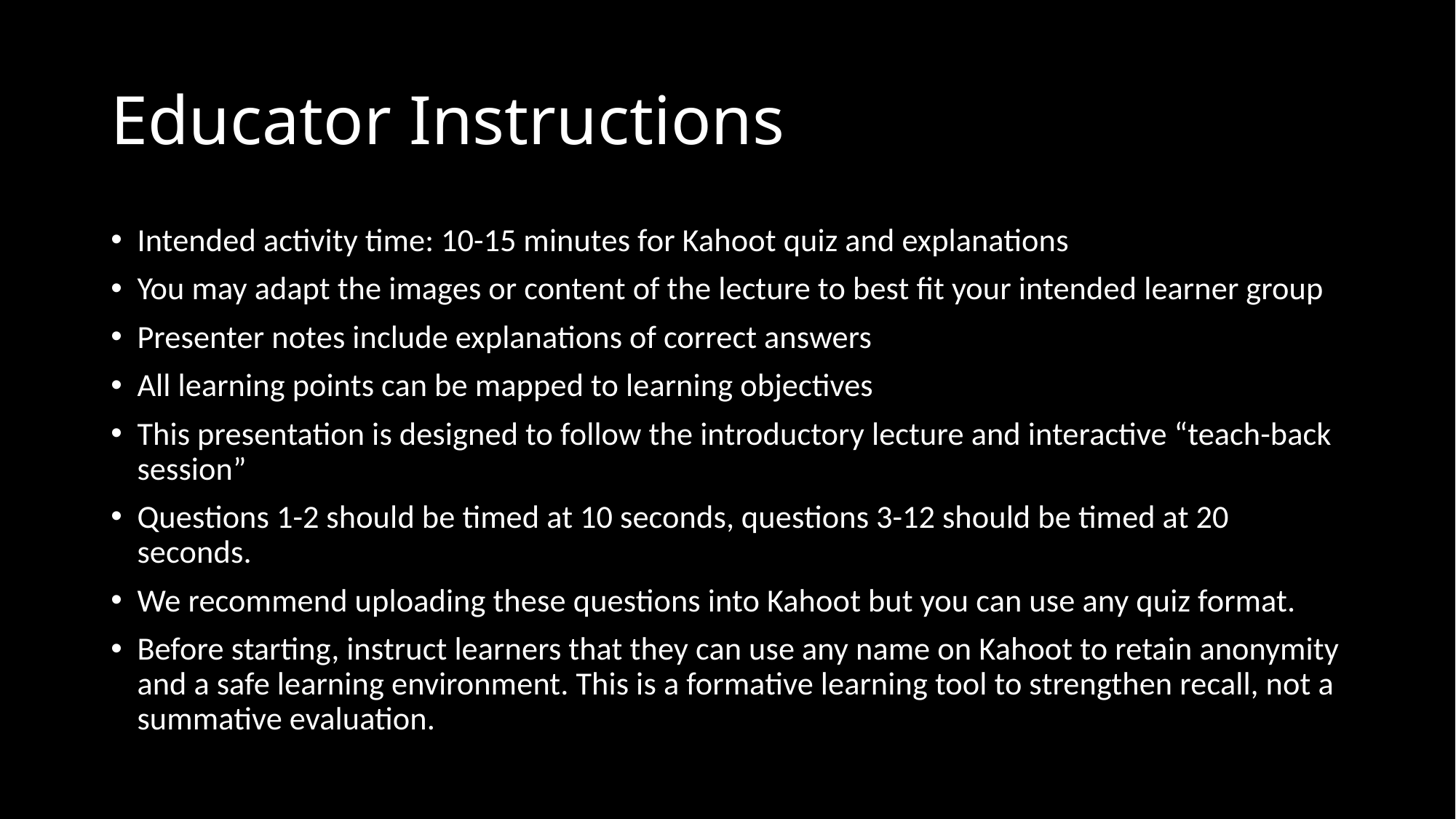

# Educator Instructions
Intended activity time: 10-15 minutes for Kahoot quiz and explanations
You may adapt the images or content of the lecture to best fit your intended learner group
Presenter notes include explanations of correct answers
All learning points can be mapped to learning objectives
This presentation is designed to follow the introductory lecture and interactive “teach-back session”
Questions 1-2 should be timed at 10 seconds, questions 3-12 should be timed at 20 seconds.
We recommend uploading these questions into Kahoot but you can use any quiz format.
Before starting, instruct learners that they can use any name on Kahoot to retain anonymity and a safe learning environment. This is a formative learning tool to strengthen recall, not a summative evaluation.

## Slide 2
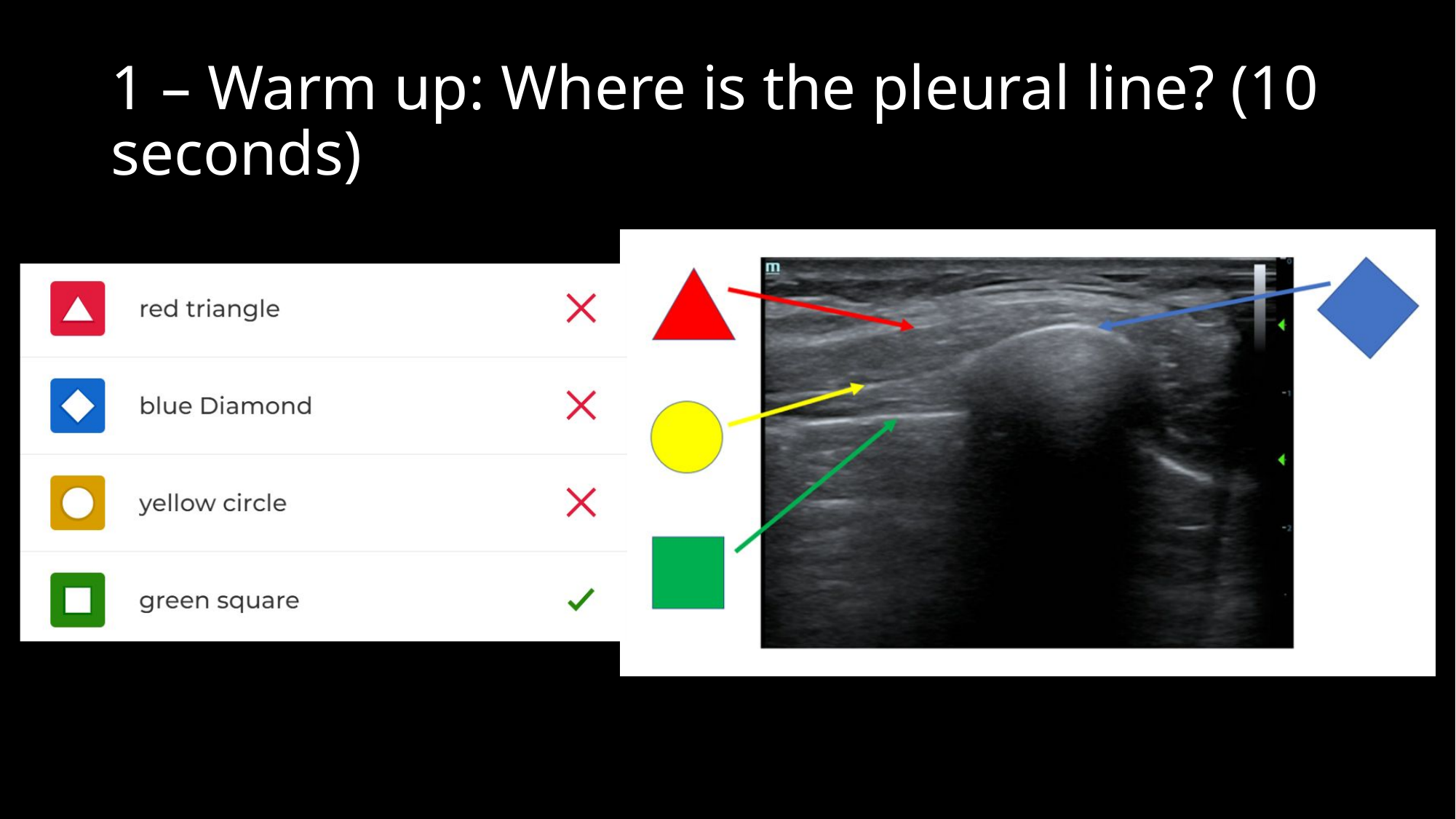

# 1 – Warm up: Where is the pleural line? (10 seconds)

## Slide 3
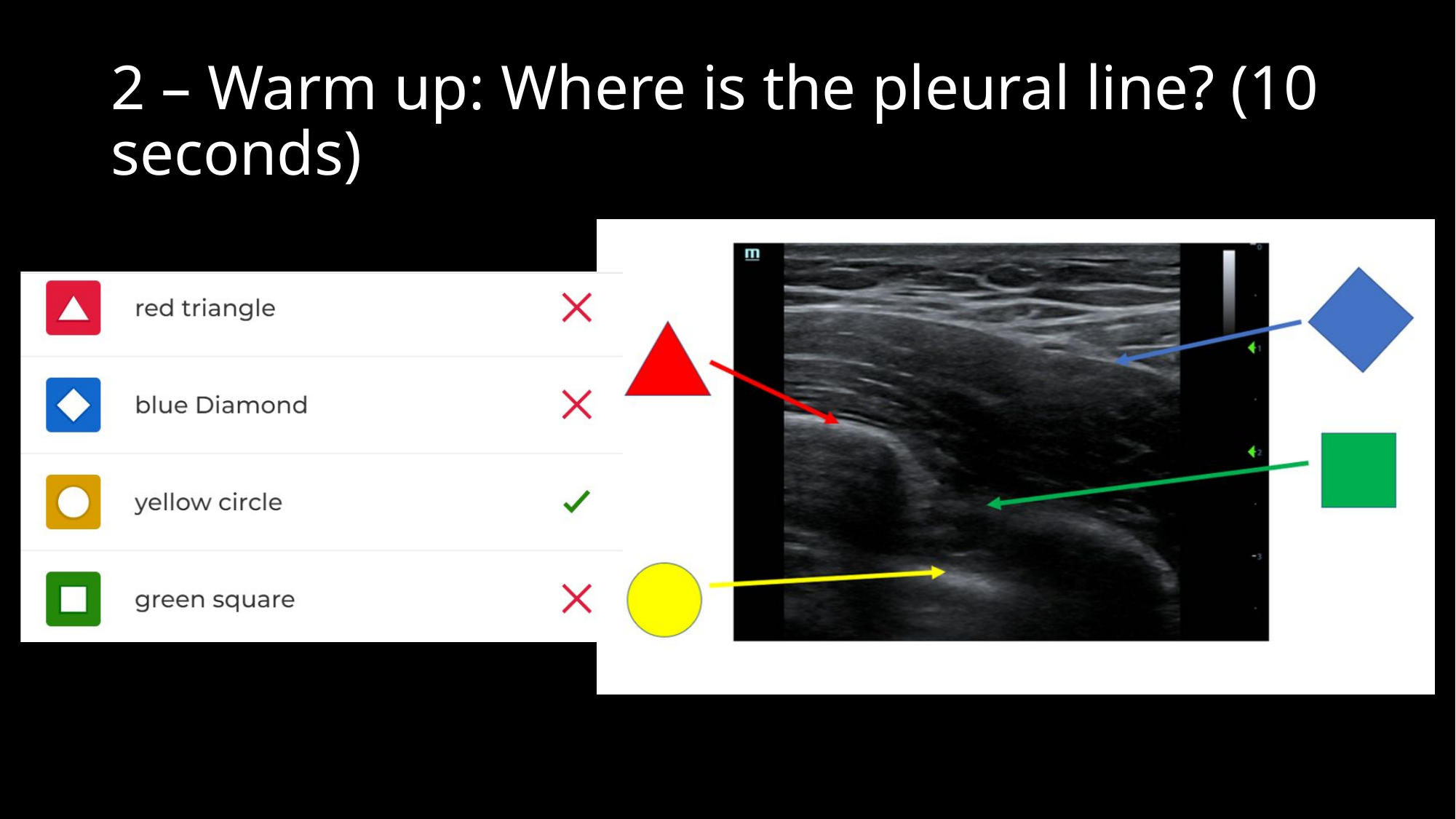

# 2 – Warm up: Where is the pleural line? (10 seconds)

## Slide 4
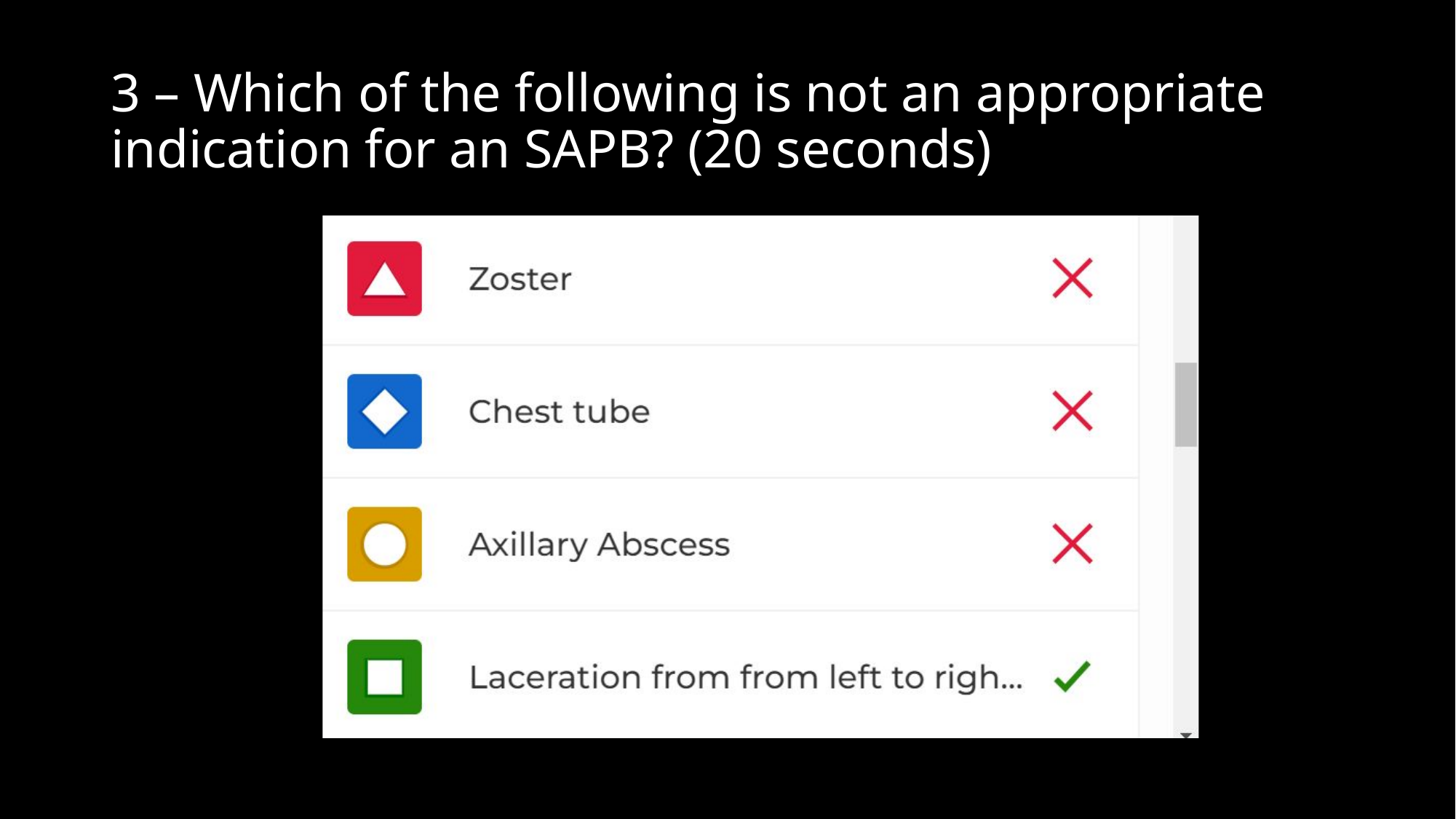

# 3 – Which of the following is not an appropriate indication for an SAPB? (20 seconds)

## Slide 5
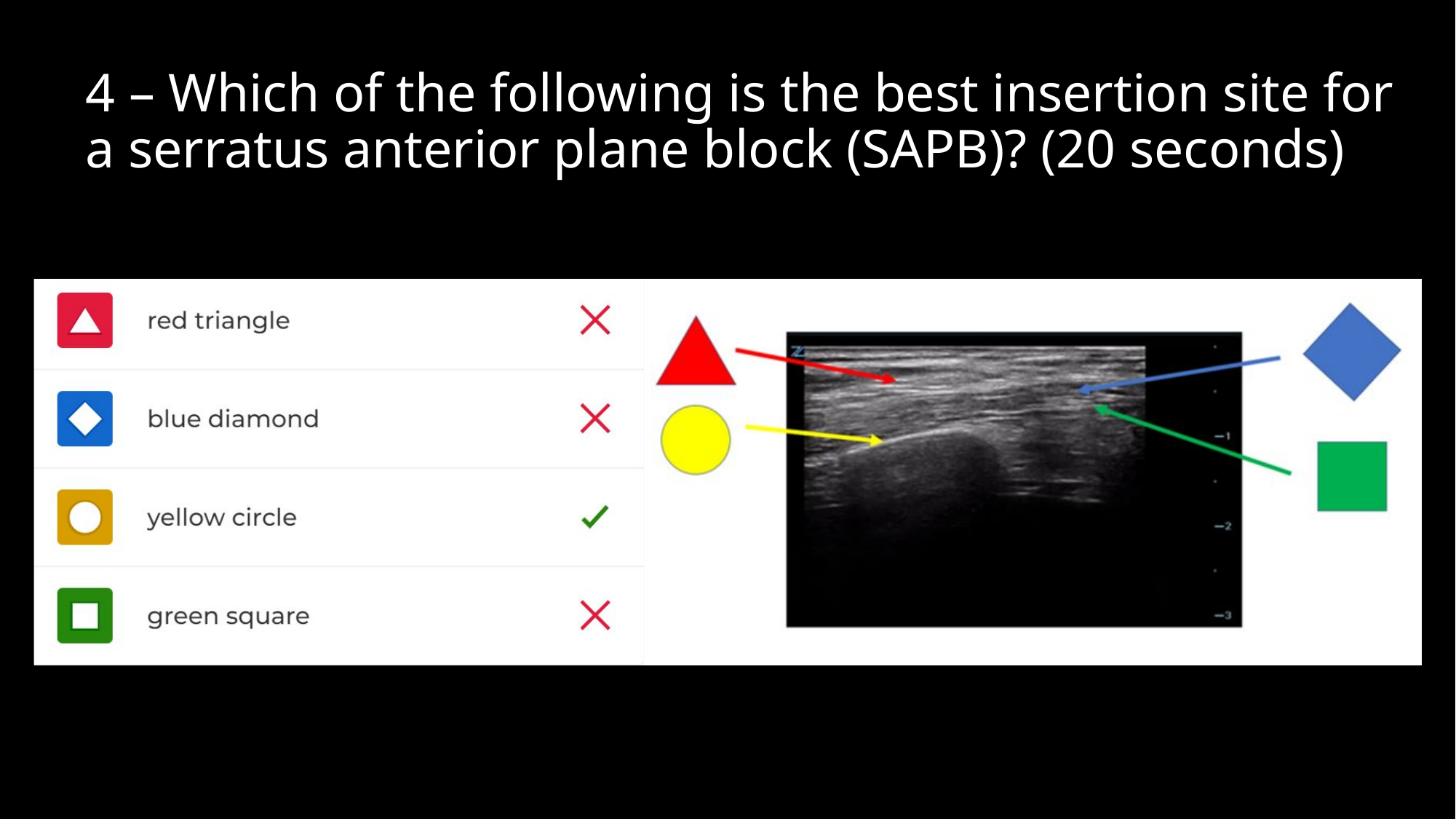

# 4 – Which of the following is the best insertion site for a serratus anterior plane block (SAPB)? (20 seconds)

## Slide 6
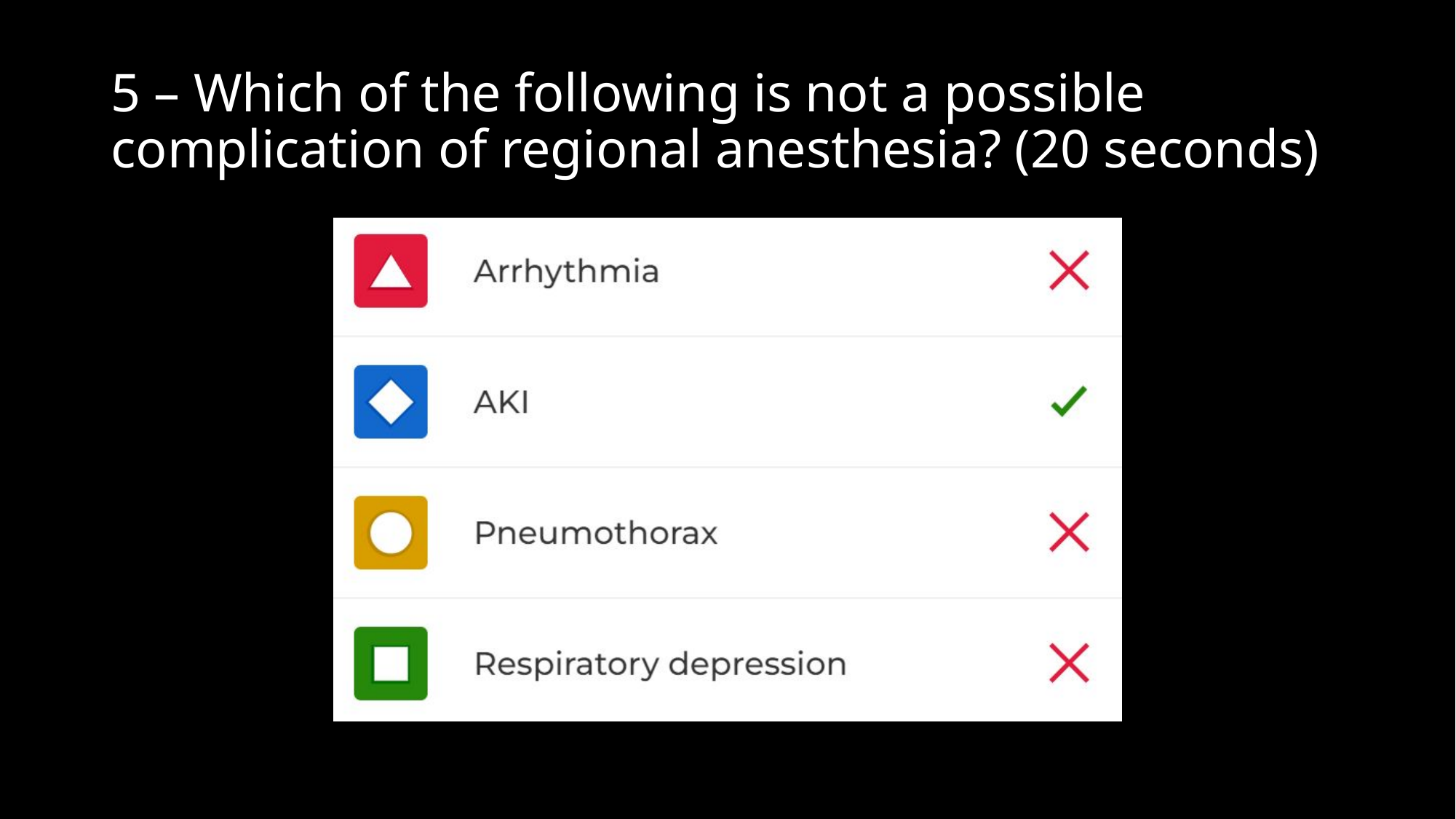

# 5 – Which of the following is not a possible complication of regional anesthesia? (20 seconds)

## Slide 7
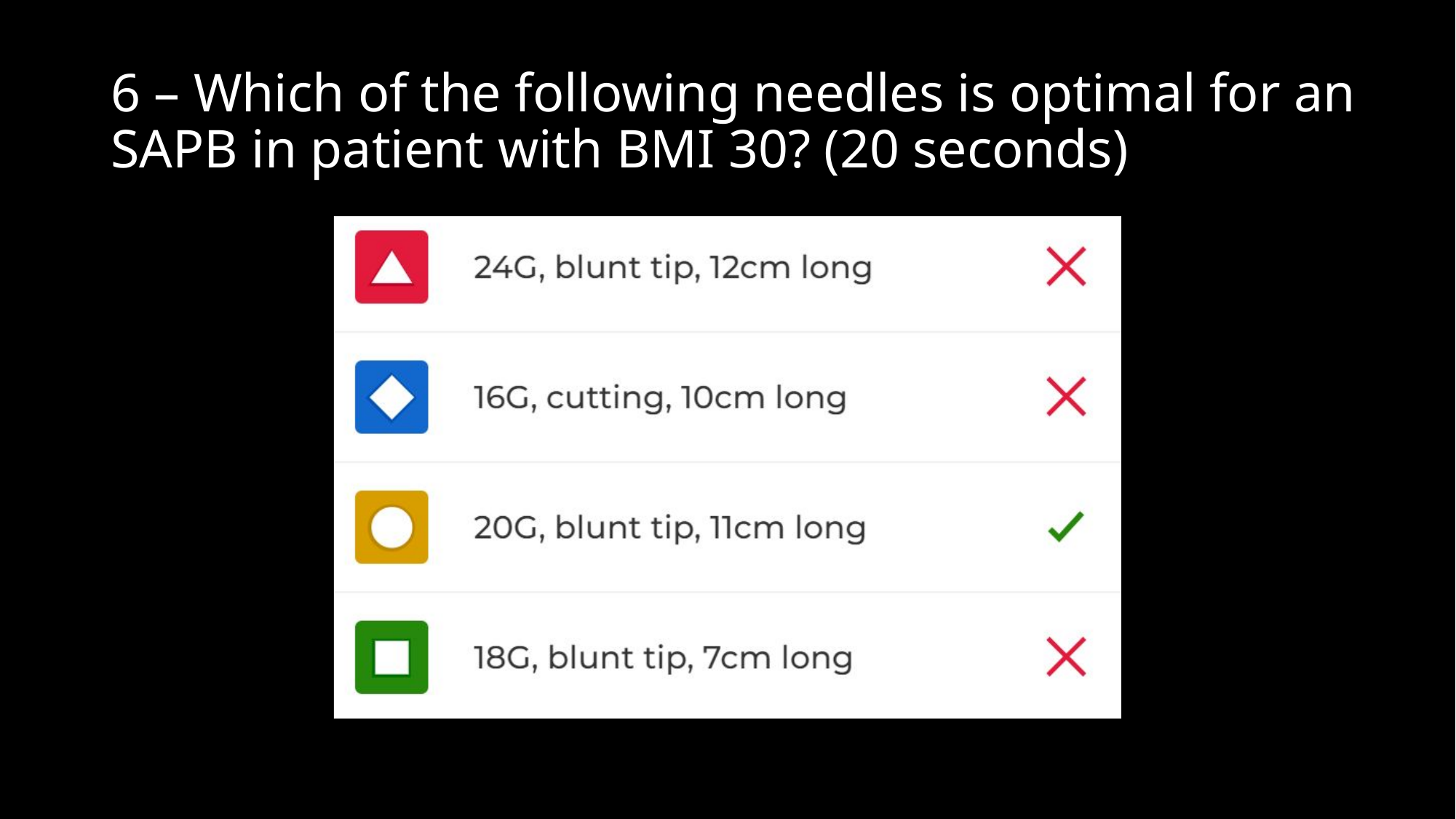

# 6 – Which of the following needles is optimal for an SAPB in patient with BMI 30? (20 seconds)

## Slide 8
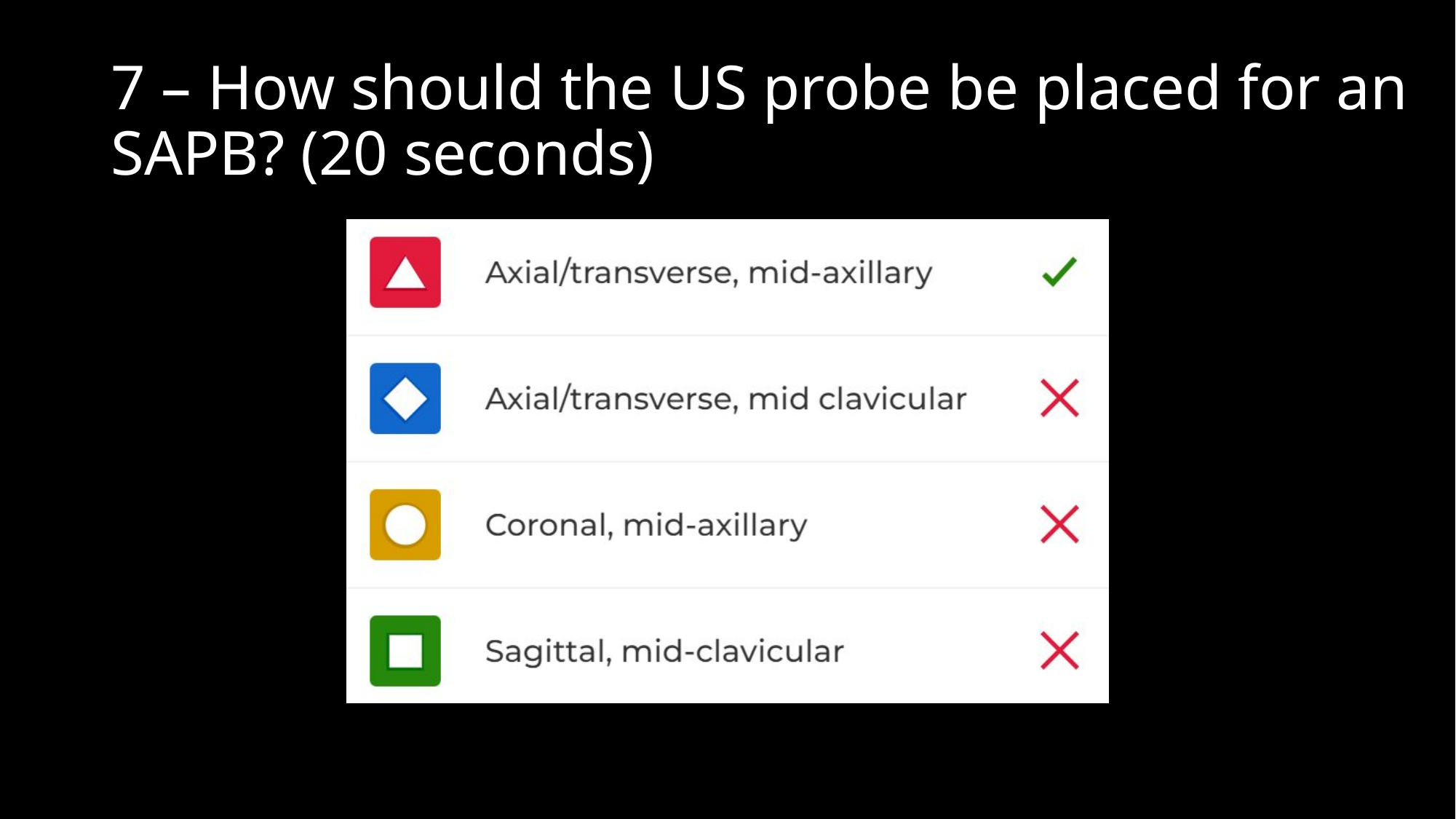

# 7 – How should the US probe be placed for an SAPB? (20 seconds)

## Slide 9
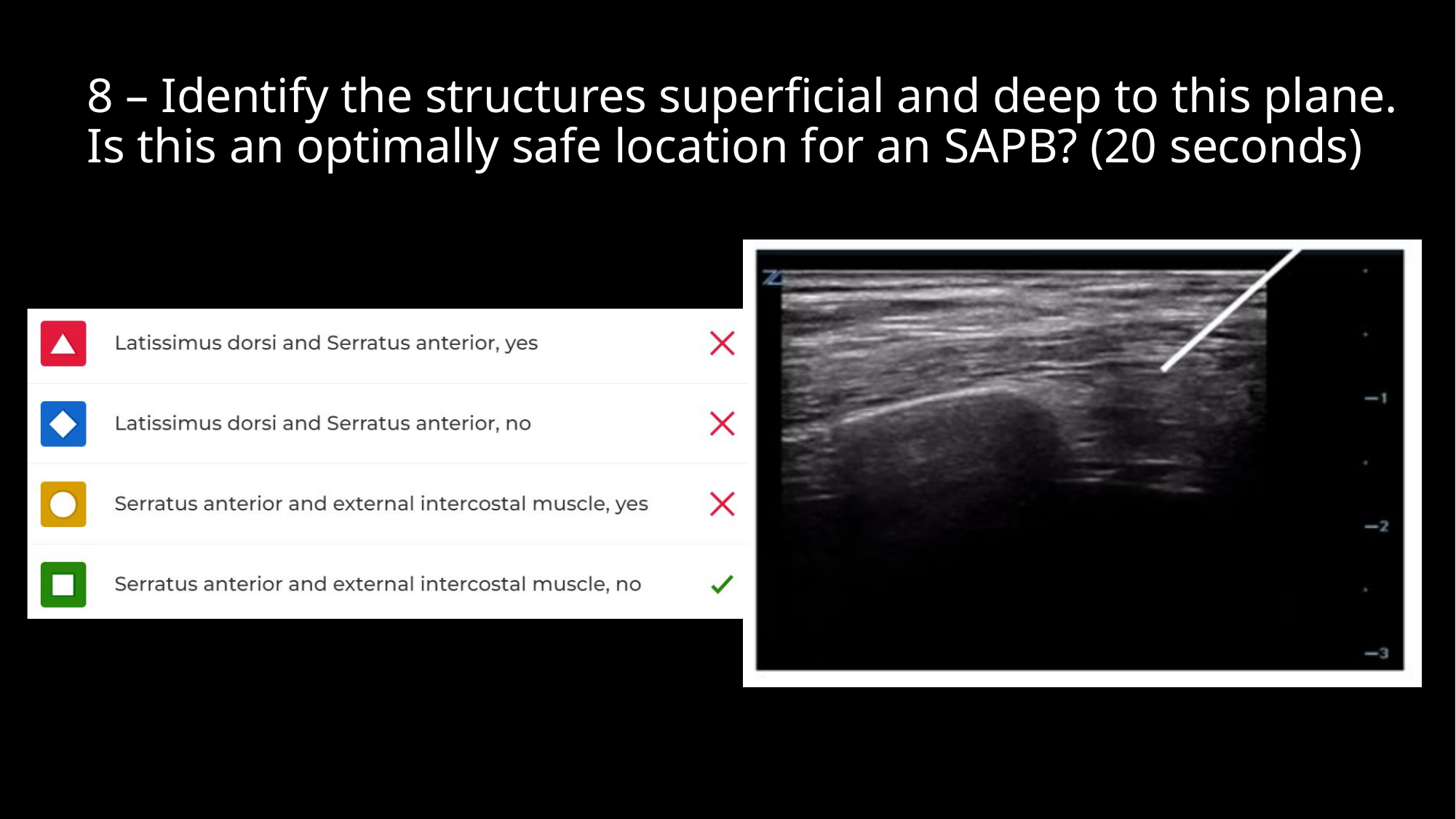

# 8 – Identify the structures superficial and deep to this plane. Is this an optimally safe location for an SAPB? (20 seconds)

## Slide 10
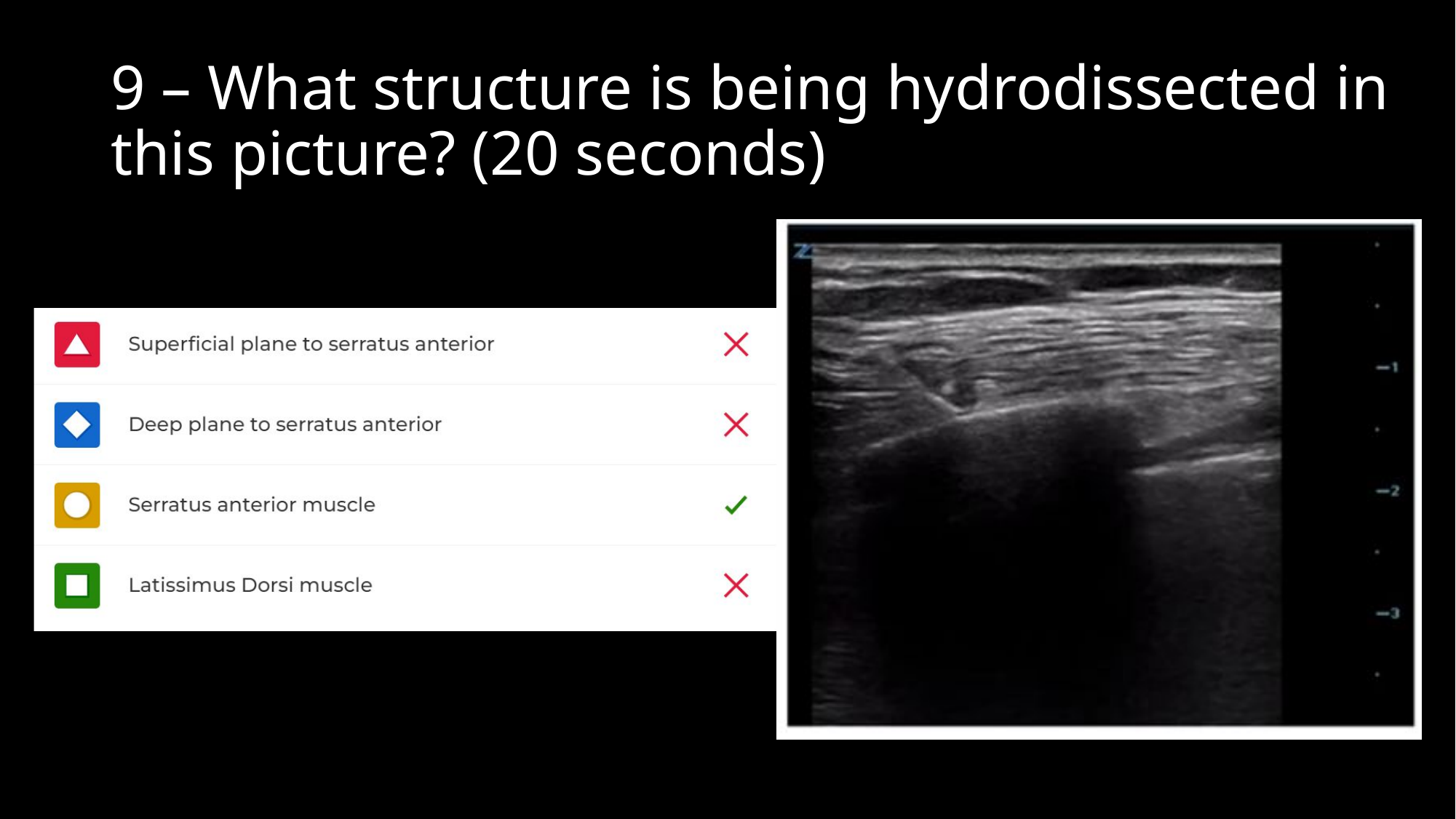

# 9 – What structure is being hydrodissected in this picture? (20 seconds)

## Slide 11
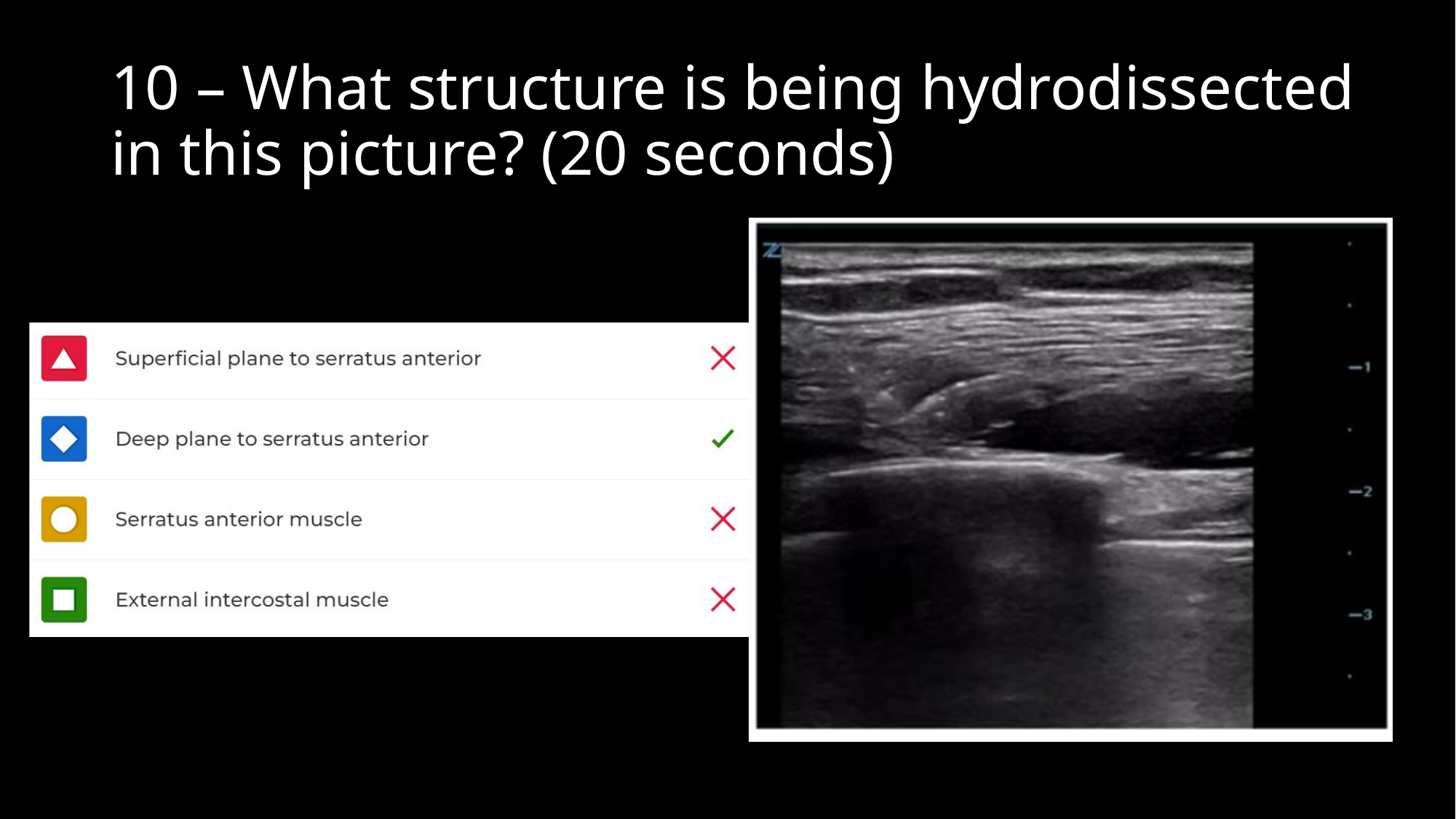

# 10 – What structure is being hydrodissected in this picture? (20 seconds)

## Slide 12
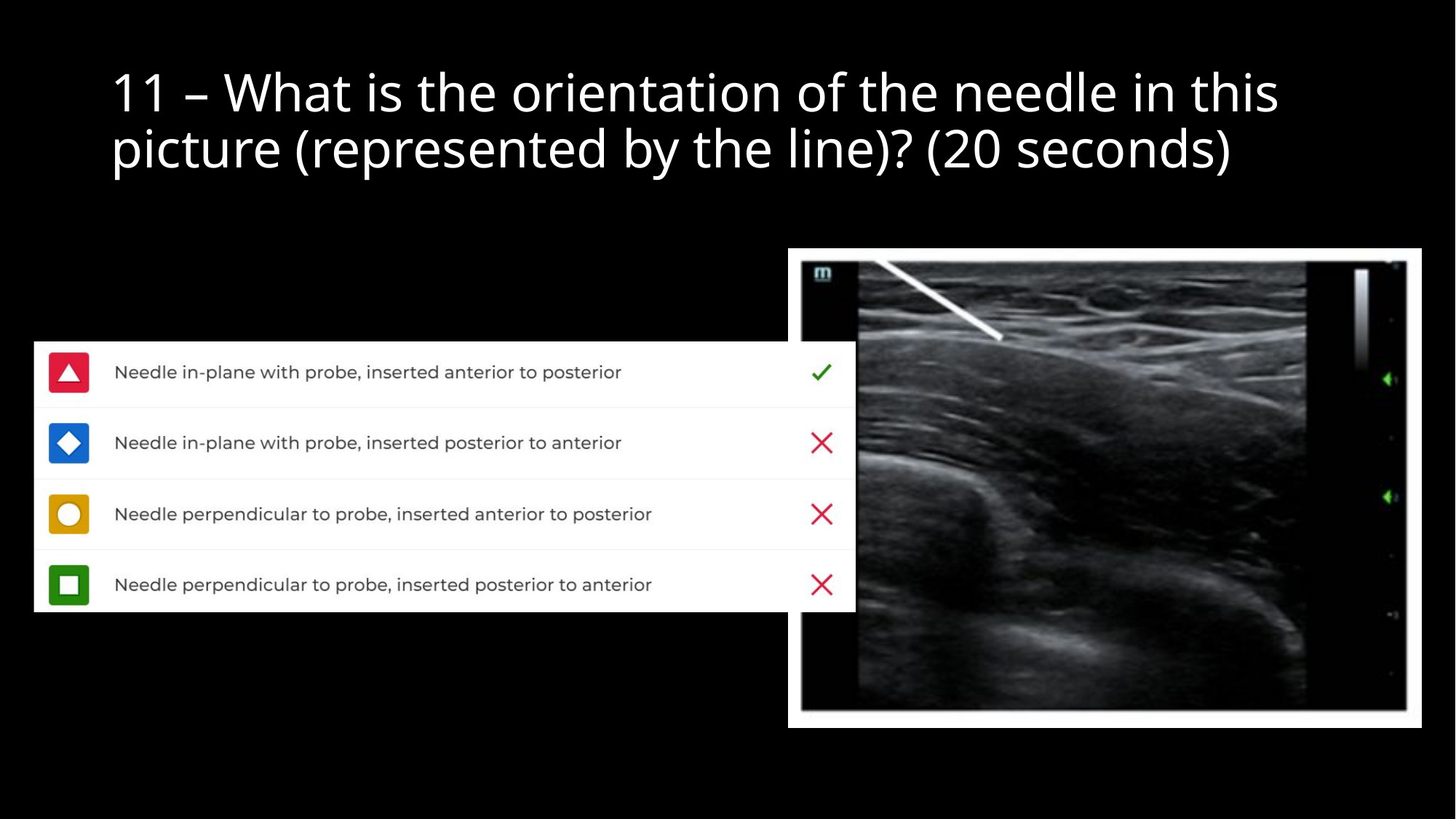

# 11 – What is the orientation of the needle in this picture (represented by the line)? (20 seconds)

## Slide 13
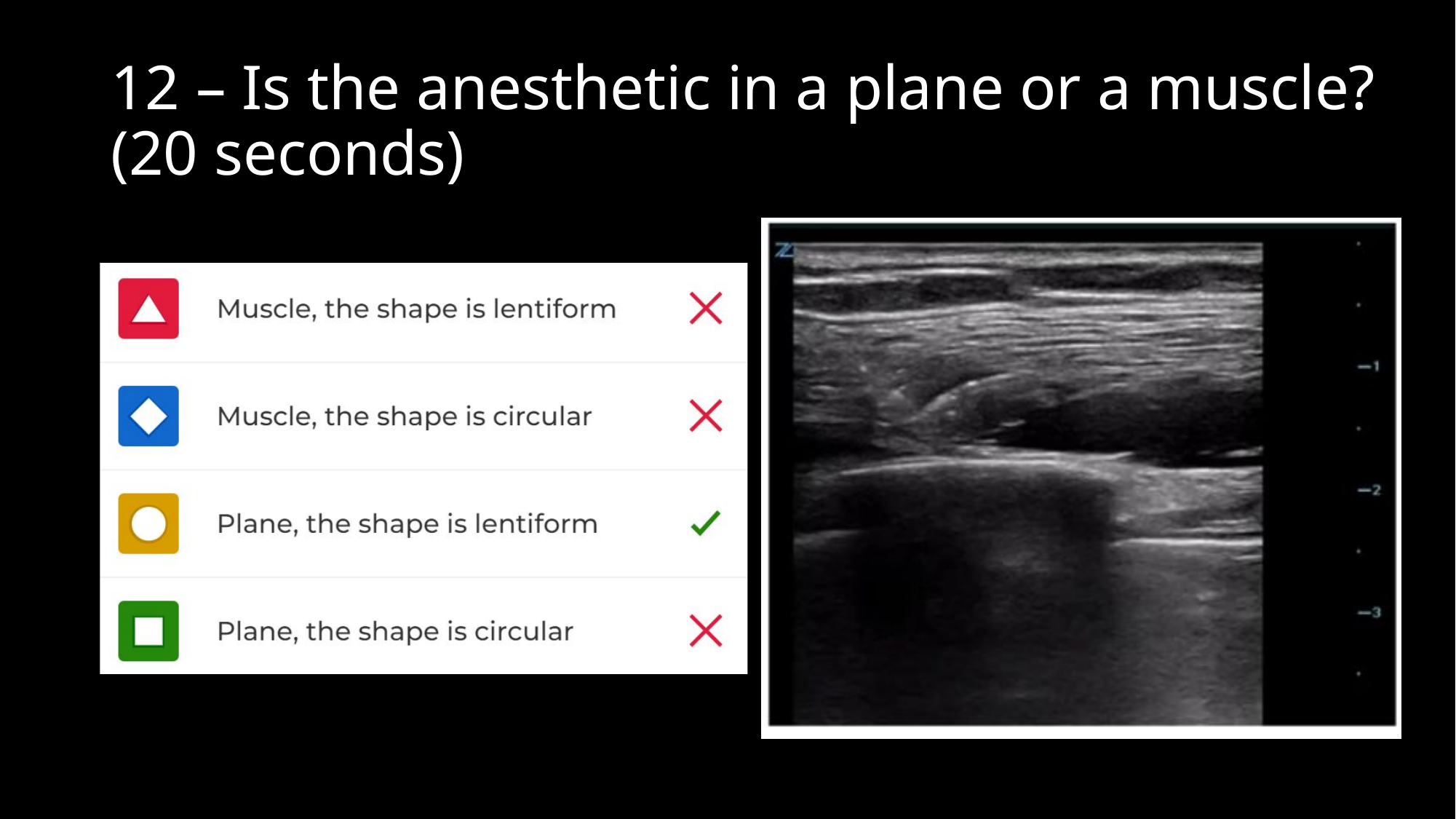

# 12 – Is the anesthetic in a plane or a muscle? (20 seconds)
