# Supplementary material for: Serratus Anterior Plane Block Remote Learning Curriculum
Source: MedEdPORTAL. 2024 Oct 25;20:11454. doi: 10.15766/mep_2374-8265.11454 (PMC11502517; doi:10.15766/mep_2374-8265.11454)
Supplement: Supplementary file 1 — Serratus Anterior Block Presentation.pptxSAPB Kahoot Quiz.pptxSAPB Proctor Instructions.docxQualtrics Presession Survey.docxQualtrics Postsession Survey.docx [file mep_2374-8265.11454-s001.zip › _Educational Summary Report_11454.pdf]

MedEdPORTAL® | The AAMC Journal of Teaching and Learning Resources

Original Publication Open Access

Serratus Anterior Plane Block Remote Learning Curriculum

Gabriel Weingart, MD*, Di Coneybeare, MD, MHPE

*Corresponding author: gabriel.weingart@gmail.com

Abstract

Introduction: Regional anesthesia aids in management of acute pain in the emergency department, but many emergency physicians remain inadequately trained. Further complicating medical education, our academic center continues to use remote learning as the primary setting for residency didactics. This project aims to create a remote conference session on ultrasound-guided serratus anterior plane blocks (USG-SAPB). **Methods:** We used the ADDIE (analyze, design, develop, implement, evaluate) model for curricular design, with emergency medicine residents as our intended learners. For the analyze element, we examined clinical need and resident program evaluation feedback. For design, we utilized best practices for remote learning, principles of mental rehearsal, and multimodal instructional theory. For develop, we completed recruitment of faculty leads, materials, and beta testing of each component. We implemented our 50-minute session on the videoconferencing platform Zoom. For evaluate, we created a program evaluation survey based on Kirkpatrick's evaluation model. **Results:** Seventeen learners completed the evaluation. For Kirkpatrick level 1, 94% reported being very or extremely satisfied. For Kirkpatrick level 2, 91% ranked their presession confidence level in performing USG-SAPB as not at all or slightly confident, compared to the postsession, where 88% ranked themselves as moderately, very, or extremely confident. For Kirkpatrick level 3, 94% reported that they would incorporate at least a moderate amount of content into clinical practice. **Discussion:** This remote learning session on USG-SAPB demonstrated high levels of learner satisfaction, improvement in learner confidence, and potential to impact learners' clinical practice. Future studies can elucidate patient outcomes related to educational sessions.

Keywords
ADDIE Model, Nerve Block, Regional Anesthesia, Remote Learning, Serratus Anterior Plane Block, Clinical/Procedural Skills Training, Emergency Medicine, Online/Distance Learning, Pain Medicine, Ultrasound Skills

Educational Objectives

By the end of this activity, learners will be able to:

1. Evaluate clinical applications of serratus anterior plane block (SAPB).
2. Identify the sonographic anatomy relevant to SAPB (latissimus dorsi, serratus anterior, rib, pleural line, intercostals).
3. Apply the procedural steps of ultrasound-guided SAPB.
4. Analyze and interpret sonographic anatomy and procedural visualization for an SAPB.

Citation:
Weingart G, Coneybeare D. Serratus anterior plane block remote learning curriculum. *MedEdPORTAL*. 2024;20:11454.
https://doi.org/10.15766/mep_2374-8265.11454

Introduction

Patient pain management is an integral aspect of emergency medicine (EM). As more evidence emerged about opioid addiction,[1,2] EM sought alternatives to opioids. One of the most promising opioid alternatives is regional anesthesia, which has been utilized for many decades in the field of anesthesia as well as in EM.[2-5] However, the use of regional anesthesia including nerve and plane blocks has not yet become ubiquitous in every emergency department. Furthermore, emergency physicians face a variety of barriers that deter the practice of regional anesthesia, continuing the reliance on opioids for orthopedic or other regional injuries and pathology.[6-8] In addition to time, space, and material constraints, many emergency physicians have not had formal training in these procedures during residency, and many residents feel inadequately trained.[9-11]

Procedural training has always been an educational challenge within residencies due to unpredictability of relevant patient presentations. Consequently, many residency programs supplement resident procedural education with simulated

Copyright © 2024 Weingart and Coneybeare This is an open-access publication distributed under the terms of the Creative Commons Attribution license.

practice in conference. The COVID-19 pandemic added an additional layer of complexity, relocating conferences to remote settings.[12,13] Many residencies have not yet returned fully to in-person conferences, and many programs have not implemented long-term solutions that allow for adequate procedural education tailored to a remote setting. While there are many available learning sessions and curricula for regional anesthesia procedural education, we found no available publications detailing implementation of remote procedural education. Furthermore, while there are several nerve block publications in *MedEdPORTAL*, none have described curricula on serratus anterior planar block (SAPB).

In this educational endeavor, we developed a remote learning session based on the ADDIE (analyze, design, develop, implement, evaluate) model[14] utilizing best practices of remote learning and principles of multimodal learning theory as well as mental rehearsal theory.

Methods

We created this learning session using the ADDIE model.[14] This educational project was deemed exempt by our institutional review board.

Analyze

Residents recurrently requested an increase in regional anesthesia education in their program evaluations. However, during the COVID pandemic, resident conferences had been moved to remote platforms and have not yet returned to in-person learning. This limitation challenged procedural education since the learners were prevented from performing the mechanical parts of the procedure during sessions. Consequently, the goal of this module was to develop a clinically relevant regional anesthesia procedural module that could be taught to residents in a remote setting. Since our emergency department was not a designated trauma center, some of the most common traumatic injuries included rib fractures, which caused pain difficult to control with oral analgesia. To make this learning module applicable to the clinical care, SAPB was selected as the focus. While many different forms of regional anesthesia could assist with analgesia for rib fractures, given our novice intended learner population, we deferred blocks with more complicated anatomy, such as erector spinae block, for future sessions.

Design

To maximize engagement and active learning, we evaluated best practices for remote learning, including small-group learning, interactivity with technology by the learner, and transformation of technology by the teacher.[12,13] We also aimed to employ multimodal education theory with a variety of learning mediums for the same objectives.[15-18] The Educational Objectives above were then designed.[19] Given that learners would have limited capacity to practice the mechanical elements during a remote conference, we employed the theories of mental rehearsal and imagery learning as a surrogate for direct mechanical skills practice.[20-24] Utilizing these concepts and bearing in mind learners' cognitive load limitations,[25-27] we designed an interactive conference session on ultrasound-guided SAPB in the remote setting. The 50-minute session was divided into three sections: didactic lecture, interactive small group, and a competitive game to facilitate recall.

Didactic lecture: A 20-minute lecture using PowerPoint was shared on the remote conference platform Zoom. The lecture (Appendix A) was delivered by the ultrasound fellow and divided into four sections correlated with the Educational Objectives. Each section of the lecture started with a question to the learners to prime them on the Educational Objective. Learners were prompted to place their answers in the chat function of Zoom to maintain engagement. Concurrently, as the presenter was going through the didactic, another faculty member monitored the chat to answer any learner questions.

Interactive small group: This interactive, 15-minute, small-group session was designed based on the principle of mental rehearsal, allowing the learners to practice the steps of the procedure by proxy.[12,13] Learners were divided into small groups of no more than eight.[12,13] Each learner was tasked with contributing at least one concrete step in instructing the faculty member on the process of performing the SAPB. Each small group was led by an ultrasound faculty member who operated a Butterfly iQ transducer. The patients were clear-plastic-wrapped animal ribs with easily identifiable anatomy on ultrasound. The faculty role-played an ultrasound novice who followed explicit instructions from the learners; each learner had to, one by one, verbalize a step in succession of the SAPB procedure and instruct the faculty member to perform the block starting from supply gathering. A video camera was aimed at the faculty's hands while the screen of the ultrasound was available via the share function of Zoom (Figure 1). Learners could see both what the faculty was doing with their hands and how it corresponded to ultrasound imaging and helped the faculty troubleshoot and direct the mechanics of performing the procedure.

Competitive quiz game: The final portion of the session involved the learners answering multiple-choice questions in competition with each other to reinforce key takeaways

Copyright © 2024 Weingart and Coneybeare This is an open-access publication distributed under the terms of the Creative Commons Attribution license.

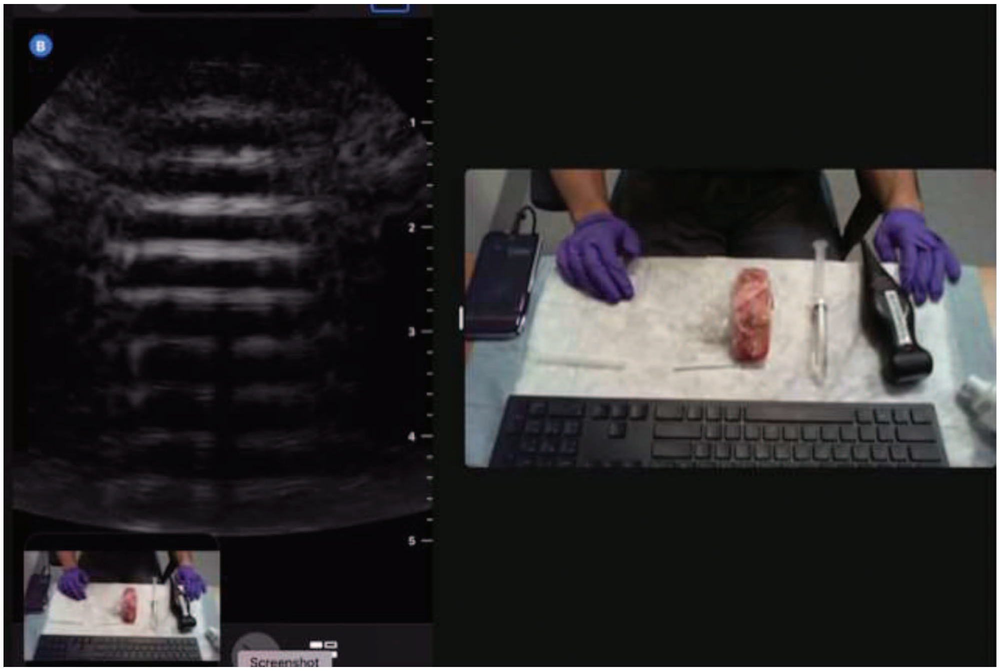

Figure 1. Small-group teach-back session setup. The left side of the screen is the ultrasound image shared on Zoom from the mobile phone. The right side is the visualization of the procedure from the computer's video camera.

points as well as to facilitate recall. The 15-minute quiz was administered through Kahoot (Appendix B). Learners submitted their names anonymously and were encouraged to use creative pseudonyms such as "ultrasound-fan." While the small group emphasized procedural collaboration, the game supported learner engagement and individual recall of information while eliminating any element of public shaming if someone got questions wrong. The highest scorer won a prize; the prize was a commercially purchased crown engraved the Greatest Serratus. The quiz questions were mapped to each Educational Objective in new contexts to emphasize analysis in addition to understanding of content. Specifically, questions 3 and 5 asked the learner to evaluate the clinical applications of SAPB; questions 1, 2, 4, and 8 to identify the sonographic anatomy relevant to SAPB; questions 6 and 7 to apply the procedural steps of ultrasound-guided SAPB; and questions 9-12 to analyze and interpret sonographic anatomy and procedural visualization for an SAPB.

Develop

We organized the logistical details of the session. The didactic session was developed to align with the Educational Objectives and provide visual correlates for the content. We obtained original images of SAPB and used the deidentified images for the presentation. We also obtained images of the procedural materials and setup. We purchased animal ribs (either beef or pork, whichever was available at the time) from local grocery stores, and using Butterfly iQ transducers, we confirmed that they had easily identifiable anatomic markers analogous to the ultrasonographic anatomy of a patient. We distributed all relevant materials for setup, including the animal ribs, spinal needles, a Butterfly iQ transducer, and charger, to each faculty member who would be proctoring a small group. Each faculty was given an ordered checklist of steps for setting up and performing the procedure that the learners had to explicitly verbalize in the small-group session (Appendix C) and was trained on implementation of this checklist to ensure consistency. Each small-group faculty member piloted the SAPB with the ribs using their iPad, connecting to Zoom, and confirming adequate visualization of both the ultrasound image and their hands performing the procedure. Switching between the didactic, interactive small group, and quiz competition was tested to ensure smooth transitions for learners. We contacted the education chief residents prior to conference day to obtain estimates for conference attendance and recruited them to aid in Zoom transitions and breakout rooms.

Weekly EM resident conference attendance was variable and included learners at multiple skill levels (medical students, physician assistants, physician assistant fellows, residents from other departments on their EM rotation, faculty, and fellows interested in the content), but the majority of learners were EM

Copyright © 2024 Weingart and Coneybeare This is an open-access publication distributed under the terms of the Creative Commons Attribution license.

residents ranging from PGY 1 to PGY 4. All fellows and faculty had completed at least the foundational ultrasound rotation during their residency as delineated by the Accreditation Council for Graduate Medical Education. Most PGY 1 and all PGY 2-PGY 4 residents had completed a formal 1-month ultrasound rotation as part of their core training. Some medical students had prior ultrasound training if they had completed an ultrasound elective. However, regional anesthesia was not part of the foundational ultrasound training that residents received in EM residency at the time, though the concepts and techniques of needle tracing in ultrasound-guided procedures were similar to those required in ultrasound-guided regional anesthesia. We also pilot-tested the quiz to confirm that learners would have appropriate time and easily visualized figures.

Implement
On the day of the module's implementation, three faculty instructors were present: one emergency ultrasound fellow and two emergency ultrasound fellowship trained faculty. Facilitators met before the day of the session to beta test the virtual instructional space, the model itself, and the steps required for learners to verbalize. One faculty gave the didactic while another monitored the chat to answer questions and post the links to initial and postsession surveys, as well as to monitor timing. For the second part, each of the faculty monitored a small group of approximately eight learners and performed the procedure on an animal rib. Three separate small groups were led by each of the faculty. The education chief residents divided the learners into the breakout rooms. For the quiz, one faculty shared the questions and explanations for the audience, while another faculty assisted with questions in the chat.

Evaluation
Learners were presented with initial and postsession surveys. The initial survey (Appendix D) aimed to assess learner education/experience and procedural/ultrasound comfort level prior to the session. The final survey (Appendix E) was developed based on Kirkpatrick's evaluation model.[28] We evaluated the first three levels of the model for satisfaction, self-reported learning, and self-reported future behavior change. Surveys were distributed as a link placed in the chat function of Zoom.

Results

Of the learners present at the session, 22 filled out the presession survey, and 17 filled out the postsession survey (Table). The presession survey also assessed how often learners performed had SAPB procedures and barriers that they had encountered to performing these procedures (Figure 2). Notably, all but one learner ($n = 21$, 95%) reported performing this procedure less than once a year, and the one learner (5%) reported performing it fewer than 10 times per year.

Twenty-four learners participated in the Kahoot quiz mapped out to the Educational Objectives. For the first objective ("Evaluate clinical applications of SAPB"), learners responded on average 88% correctly to the questions. For the second ("Identify the sonographic anatomy relevant to SAPB [latissimus dorsi, serratus anterior, rib, pleural line, intercostals]"), learners responded on average 30% correctly to the questions. For the third ("Apply the procedural steps of ultrasound-guided SAPB"), learners responded on average 65% correctly to the questions. For the fourth ("Analyze and interpret sonographic anatomy and procedural visualization for an SAPB"), learners responded on average 42% correctly to the questions.

For program evaluation, to evaluate Kirkpatrick level 1: learner reactions, we assessed learner satisfaction with the session (Table). All but one responder ($n = 16$, 94%) reported that they were very or extremely satisfied with the session. Sixteen (94%) also reported that they would be interested in future sessions, with one "maybe" (Table).

We also assessed presession and postsession learner confidence in performing the procedure as a marker of Kirkpatrick level 2: learning. Initially, 77% of learners ($n = 17$) were not at all confident, 14% ($n = 3$) were slightly confident, and 9% ($n = 2$) were moderately confident, with no responders expressing that they were very or extremely confident. After the session, no learners were not at all confident, while 12% ($n = 2$) were slightly confident, 59% ($n = 10$) moderately confident, 24% ($n = 4$) very confident, and 6% ($n = 1$) extremely confident (Figure 3).

All responders believed that the SAPB session was very ($n = 6$, 35%) or extremely ($n = 11$, 65%) relevant to clinical practice, with no learners replying that it was not at all, slightly, or moderately relevant (Table). As a marker for Kirkpatrick level 3: behavior change, we asked for learners' plans to incorporate the learning points into clinical practice. No learners planned to incorporate none of the session, while 6% ($n = 1$) planned to incorporate a little, 35% ($n = 6$) a moderate amount, 29% ($n = 5$) a lot, and 29% ($n = 5$) a great deal (Table).

Discussion

Remote learning presents a challenge to procedure teaching and learning. Here, we offer an SAPB remote learning session. Although learners did not have the opportunity to directly

Copyright © 2024 Weingart and Coneybeare This is an open-access publication distributed under the terms of the Creative Commons Attribution license.

Table. Pre- and Postsession Survey Reponses

Item	Presession	Postsession	Postsession Responses
Level of medical training			
Medical student	1	0	
EM1	6	4	
EM2	2	3	
EM3	2	3	
EM4	3	3	
Non-EM resident	0	0	
Attending	5	1	
PA/NP	0	0	
PA fellow/resident	3	3	
Other	0	0	
Total	22	17	
Learner satisfaction with session[a]			
Not at all			0
Slightly			0
Moderately			1
Very			6
Extremely			10
Interest in similar future sessions			
Yes			16
No			0
Maybe			1
Relevant to clinical practice[a]			
Not at all			0
Slightly			0
Moderately			0
Very			6
Extremely			11
How much will you incorporate?[a]			
None			0
A little			1
A moderate amount			6
A lot			5
A great deal			5

Abbreviations: EM, emergency medicine; NP, nurse practitioner; PA, physician assistant.
[a]Rated on a 5-point scale (response options are listed in the table).

practice the mechanics of SAPB with their own hands, they still benefited from the deliberate mental rehearsal. Learners reported overall satisfaction with the session and noted that it was quite relevant to clinical practice. The pre- and postsession procedural comfort graph (Figure 3) demonstrates a rightward shift and significantly improved procedural confidence. However, when tested on recall of information from the module during the session, learners performed poorly to moderately well. This is consistent with our learners' baseline unfamiliarity with regional anesthesia as most of the learners indicated no prior experience with these procedures.

In the context of limited in-person education, educators can implement remote sessions for procedural education analogous to this curriculum to offer programming beyond a lecture. These lesson designs not only function within the constraints of a pandemic but can also apply to any program that requires distance learning. A recent meta-analysis on remote learning in postgraduate medical education found that learners had improved engagement and retention when small-group learning was incorporated into the curriculum.[13] However, our quiz results show that a single session is inadequate to prepare learners for real-life practice. This supports existing studies on the deliberate practice and spaced repetition needed to reinforce concepts and skills towards competency. Despite the less optimal quiz results, learners indicated nearly unanimously that they would be interested in similar learning sessions. Continued engagement of learners and their motivation to reinforce learning will be key for future sessions and longitudinal commitment.

We also utilized the theories of multimodal learning theory and mental rehearsal to maximize engagement and intake of procedural information. Multimodal learning theory states that by engaging multiple sensory learning pathways, training time can be shortened and learning outcomes can be improved without increasing strain on cognitive load.[15,17] Therefore, we

Copyright © 2024 Weingart and Coneybeare This is an open-access publication distributed under the terms of the Creative Commons Attribution license.

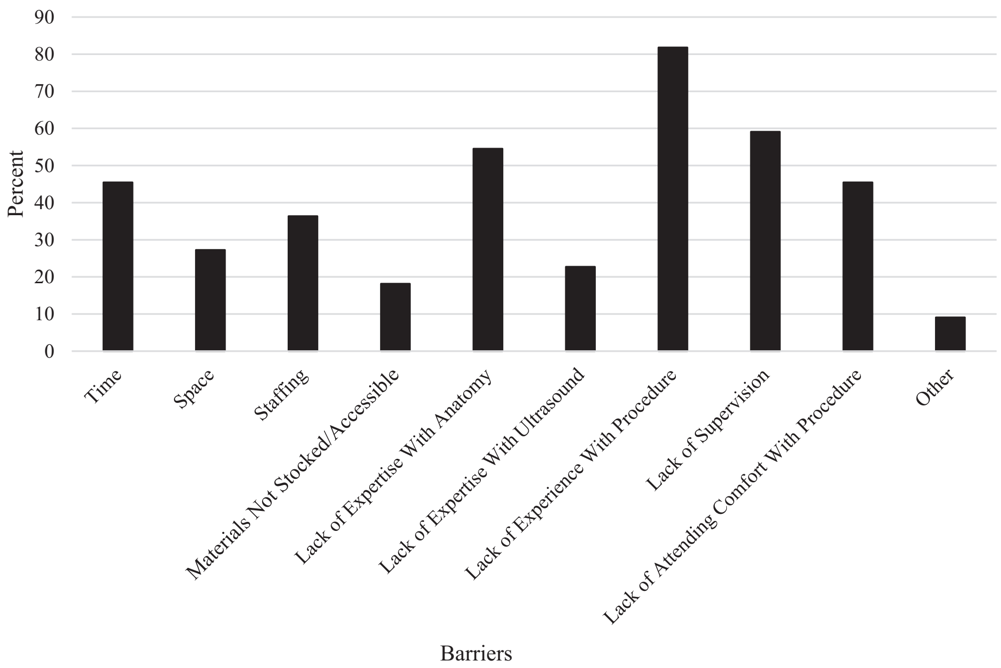

Figure 2. Barriers to performing serratus anterior plane blocks in the emergency department.

designed our session with multiple mediums to reinforce the Educational Objectives. In order to circumvent the exorbitant cost of resources on handheld ultrasounds for every learner, we utilized the principles of mental rehearsal to craft a session of proxy practice. Numerous studies stemming from competitive sports demonstrate the efficacy of mental rehearsal, foremost determining that utilization of imagery in practicing free throws in basketball results in an amount of improvement comparable to physically practicing free throws.[21] The medical literature has attempted to replicate these results and obtained similar findings, especially when evaluating learners' improvement in rudimentary surgical skills.[20,22,23]

We surveyed self-reported measures of learning and behavioral change, which may not be the most reflective of actual change. We utilized the Kirkpatrick model to evaluate the perceived effectiveness of the session and help guide creation of future sessions. To better assess Kirkpatrick level 3, future studies

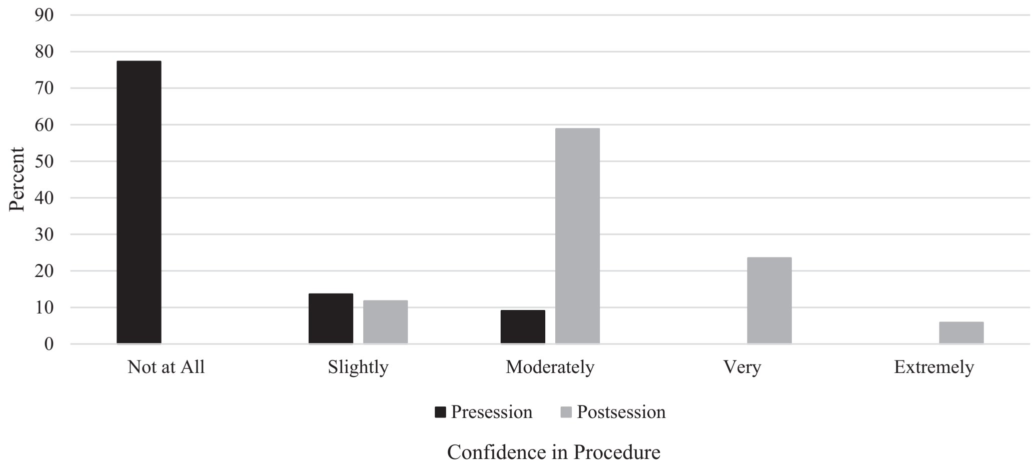

Figure 3. Pre- and postsession learner confidence in performing serratus anterior plane blocks.

Copyright © 2024 Weingart and Coneybeare This is an open-access publication distributed under the terms of the Creative Commons Attribution license.

could longitudinally follow learners before and after the session to assess change in behavior and clinical utilization of regional anesthesia with SAPBs. Future sessions could implement spaced repetition to refresh and strengthen existing knowledge. Future research could also evaluate Kirkpatrick level 4 with longitudinal changes in patient pain scores, length of stay, and disposition to determine the more widespread clinical effects of these educational interventions.

The implementation of this session had multiple limitations. The data collected were derived from a convenience sample of learners of varying levels of skill who happened to be present at the EM resident conference that morning. In addition, the data collected differed between the presurvey and postsurvey depending on which learners completed the forms. We recognize that this session operated within a resource-rich environment where we could rely on multiple expert small-group leaders as well as the use of technology (e.g., Butterfly iQ) not readily available to all programs. Although the module began to address the challenges of remote procedural education, a single session could not provide the longitudinal learning and deliberate practice required to achieve proficiency. Spaced repetition and longitudinal learning were outside the scope of this singular conference session. Furthermore, the module did not address many of the systemic barriers that deter the practice of regional anesthesia. Lastly, while the pre- and postsurveys were created based on Kirkpatrick's framework, we did not seek to collect validity evidence on the quiz questions or the pre- and postsurveys. Future studies could perform psychometric measures to ensure reliability and internal consistency.

In the setting of remote education of procedure learning, we adopted best practices of remote learning and incorporated established learning theories of multimodal learning and mental rehearsal. This remote learning session on ultrasound-guided SAPB demonstrated high levels of learner satisfaction, as well as improvement in learner confidence, and can potentially contribute to learners' clinical practice. Future studies can further elucidate patient outcomes related to educational sessions.

Appendices

A. Serratus Anterior Block Presentation.pptx

B. SAPB Kahoot Quiz.pptx

C. SAPB Proctor Instructions.docx

D. Qualtrics Presession Survey.docx

E. Qualtrics Postsession Survey.docx

All appendices are peer reviewed as integral parts of the Original Publication.

Gabriel Weingart, MD: Assistant Professor, Department of Emergency Medicine, Columbia University Irving Medical Center; ORCID: https://orcid.org/0009-0002-2774-4993

Di Coneybeare, MD, MHPE: Assistant Professor, Department of Emergency Medicine, and Clinical Ultrasound Fellowship Director, Columbia University Irving Medical Center; ORCID: https://orcid.org/0000-0002-9027-435X

Disclosures
None to report.

Funding/Support
None to report.

Ethical Approval
The Columbia Institutional Review Board deemed further review of this project not necessary.

References

1. Reider B. Opioid epidemic. *Am J Sports Med*. 2019;47(5): 1039-1042. https://doi.org/10.1177/0363546519836727
2. Lavonas EJ, Dezfulian C. Impact of the opioid epidemic. *Crit Care Clin*. 2020;36(4):753-769. https://doi.org/10.1016/j.ccc.2020.07.006
3. Bedene A, Dahan A, Rosendaal FR, van Dorp ELA. Opioid epidemic: lessons learned and updated recommendations for misuse involving prescription versus non-prescription opioids. *Expert Rev Clin Pharmacol*. 2022;15(9):1081-1094. https://doi.org/10.1080/17512433.2022.2114898
4. Nicholas TA IV, Robinson R. Multimodal analgesia in the era of the opioid epidemic. *Surg Clin North Am*. 2022;102(1):105-115. https://doi.org/10.1016/j.suc.2021.09.003
5. Prabhakar A, Lambert T, Kaye RJ, et al. Adjuvants in clinical regional anesthesia practice: a comprehensive review. *Best Pract Res Clin Anaesthesiol*. 2019;33(4):415-423. https://doi.org/10.1016/j.bpa.2019.06.001
6. Amini R, Kartchner JZ, Nagdev A, Adhikari S. Ultrasound-guided nerve blocks in emergency medicine practice. *J Ultrasound Med*. 2016;35(4):731-736. https://doi.org/10.7863/ultra.15.05095
7. Lee HK, Kang BS, Kim CS, Choi HJ. Ultrasound-guided regional anesthesia for the pain management of elderly patients with hip

Copyright © 2024 Weingart and Coneybeare This is an open-access publication distributed under the terms of the Creative Commons Attribution license.

fractures in the emergency department. *Clin Exp Emerg Med*. 2014;1(1):49-55. https://doi.org/10.15441/ceem.14.008

8. Stone A, Goldsmith AJ, Pozner CN, Vlassakov K. Ultrasound-guided regional anesthesia in the emergency department: an argument for multidisciplinary collaboration to increase access while maintaining quality and standards. *Reg Anesth Pain Med*. 2021;46(9):820-821. https://doi.org/10.1136/rapm-2020-102416
9. Tucker RV, Peterson WJ, Mink JT, et al. Defining an ultrasound-guided regional anesthesia curriculum for emergency medicine. *AEM Educ Train*. 2021;5(3):e10557. https://doi.org/10.1002/aet2.10557
10. Wiercigroch D, Ben-Yakov M, Porplycia D, Friedman SM. Regional anesthesia in Canadian emergency departments: emergency physician practices, perspectives, and barriers to use. *CJEM*. 2020;22(4):499-503. https://doi.org/10.1017/cem.2020.51
11. Wilson CL, Chung K, Fong T. Challenges and variations in emergency medicine residency training of ultrasound-guided regional anesthesia techniques. *AEM Educ Train*. 2017;1(2):158-164. https://doi.org/10.1002/aet2.10014
12. Daniel M, Gordon M, Patricio M, et al. An update on developments in medical education in response to the COVID-19 pandemic: a BEME scoping review: BEME Guide no. 64. *Med Teach*. 2021;43(3):253-271. https://doi.org/10.1080/0142159X.2020.1864310
13. Khamees D, Peterson W, Patricio M, et al. Remote learning developments in postgraduate medical education in response to the COVID-19 pandemic—a BEME systematic review: BEME Guide no. 71. *Med Teach*. 2022;44(5):466-485. https://doi.org/10.1080/0142159X.2022.2040732
14. Branch RM. *Instructional Design: The ADDIE Approach*. Springer; 2009.
15. Barton G, Ryan M. Multimodal approaches to reflective teaching and assessment in higher education. *High Educ Res Dev*. 2014; 33(3):409-424. https://doi.org/10.1080/07294360.2013.841650
16. Bloomfield JG, Cornish JC, Parry AM, Pegram A, Moore JS. Clinical skills education for graduate-entry nursing students: enhancing learning using a multimodal approach. *Nurse Educ Today*. 2013;33(3):247-252. https://doi.org/10.1016/j.nedt.2011.11.009
17. Gellevij M, Van Der Meij H, De Jong T, Pieters J. Multimodal versus unimodal instruction in a complex learning context. *J Exp Educ*. 2002;70(3):215-239. https://doi.org/10.1080/00220970209599507
18. Ruthberg JS, Quereshy HA, Ahmadmehrabi S, et al. A multimodal multi-institutional solution to remote medical student education for otolaryngology during COVID-19. *Otolaryngol Head Neck Surg*. 2020;163(4):707-709. https://doi.org/10.1177/0194599820933599
19. Adams NE. Bloom's taxonomy of cognitive learning objectives. *J Med Libr Assoc*. 2015;103(3):152-153. https://doi.org/10.3163/1536-5050.103.3.010
20. Arora S, Aggarwal R, Sirimanna P, et al. Mental practice enhances surgical technical skills: a randomized controlled study. *Ann Surg*. 2011;253(2):265-270. https://doi.org/10.1097/SLA.0b013e318207a789
21. Lerner BS, Ostrow AC, Yura MT, Etzel EF. The effects of goal-setting and imagery training programs on the free-throw performance of female collegiate basketball players. *Sport Psychol*. 1996;10(4):382-397. https://doi.org/10.1123/tsp.10.4.382
22. Sanders CW, Sadoski M, Bramson R, Wiprud R, Van Walsum K. Comparing the effects of physical practice and mental imagery rehearsal on learning basic surgical skills by medical students. *Am J Obstet Gynecol*. 2004;191(5):1811-1814. https://doi.org/10.1016/j.ajog.2004.07.075
23. Sanders CW, Sadoski M, van Walsum R, Bramson R, Wiprud R, Fossum TW. Learning basic surgical skills with mental imagery: using the simulation centre in the mind. *Med Educ*. 2008;42(6): 607-612. https://doi.org/10.1111/j.1365-2923.2007.02964.x
24. Rao A, Tait I, Alijani A. Systematic review and meta-analysis of the role of mental training in the acquisition of technical skills in surgery. *Am J Surg*. 2015;210(3):545-553. https://doi.org/10.1016/j.amjsurg.2015.01.028
25. de Jong T. Cognitive load theory, educational research, and instructional design: some food for thought. *Instr Sci*. 2010;38(2): 105-134. https://doi.org/10.1007/s11251-009-9110-0
26. Paas F, van Gog T, Sweller J. Cognitive load theory: new conceptualizations, specifications, and integrated research perspectives. *Educ Psychol Rev*. 2010;22(2):115-121. https://doi.org/10.1007/s10648-010-9133-8
27. Sweller J. Cognitive load theory and educational technology. *Educ Technol Res Dev*. 2020;68(1):1-16. https://doi.org/10.1007/s11423-019-09701-3
28. Praslova L. Adaptation of Kirkpatrick's four level model of training criteria to assessment of learning outcomes and program evaluation in higher education. *Educ Assess Eval Account*. 2010;22(3):215-225. https://doi.org/10.1007/s11092-010-9098-7

Received: December 28, 2023
Accepted: June 14, 2024
Published: October 25, 2024

Copyright © 2024 Weingart and Coneybeare This is an open-access publication distributed under the terms of the Creative Commons Attribution license.
